# Extracellular Sortilin Proteopathy Relative to β-Amyloid and Tau in Aged and Alzheimer’s Disease Human Brains

**DOI:** 10.3389/fnagi.2020.00093

**Published:** 2020-05-12

**Authors:** Tian Tu, Juan Jiang, Qi-Lei Zhang, Lily Wan, Ya-Nan Li, Aihua Pan, Jim Manavis, Xiao-Xin Yan

**Affiliations:** ^1^Department of Anatomy and Neurobiology, Xiangya School of Medicine, Central South University, Changsha, China; ^2^Center for Morphological Sciences, Xiangya School of Medicine, Central South University, Changsha, China; ^3^Faculty of Health and Medical Sciences, The University of Adelaide, Adelaide, SA, Australia

**Keywords:** ABC score, brain banking, dementia, neurodegeneration, neuritic plaque, Vps10p

## Abstract

Amyloid plaques and neurofibrillary tangles (NFTs) are hallmark lesions of Alzheimer’s disease (AD) related to β-amyloid (Aβ) deposition and intraneuronal phosphorylated tau (pTau) accumulation. *Sor*tilin C-terminal *fra*gments (shortened as “sorfra”) can deposit as senile plaque-like lesions within AD brains. The course and pattern of sorfra plaque formation relative to Aβ and pTau pathogenesis remain unknown. In the present study, cerebral and subcortical sections in postmortem human brains (*n* = 46) from aged and AD subjects were stained using multiple markers (6E10, β-secretase 1, pTau, and sortilin antibodies, as well as Bielschowsky silver stain). The course and pattern of sorfra plaque formation relative to Thal Aβ and Braak NFT pathogenic stages were determined. Sorfra plaques occurred in the temporal, inferior frontal and occipital neocortices in cases with Thal 1 and Braak III stages. They were also found additionally in the hippocampal formation, amygdala, and associative neocortex in cases with Thal 2–4 and Braak IV–V. Lastly, they were also found in the primary motor, somatosensory, and visual cortices in cases with Thal 4–5 and Braak VI. Unlike Aβ and pTau pathologies, sorfra plaques did not occur in subcortical structures in cases with Aβ/pTau lesions in Thal 3–5/Braak IV–VI stages. We establish here that sorfra plaques are essentially a cerebral proteopathy. We believe that the development of sorfra plaques in both cortical and hippocampal regions proceeds in a typical spatiotemporal pattern, and the stages of cerebral sorfra plaque formation partially overlap with that of Aβ and pTau pathologies.

## Introduction

Senile plaque deposition and neurofibrillary tangle (NFT) formation are considered as the two primary neuropathological features in Alzheimer’s disease (AD); both were initially identified in human brains using classic histological preparations (i.e., silver and tinctorial amyloid stains) (Critchley, 1929; Oifa, 1973; García-Marín et al., 2007; Ohry and Buda, 2015). The β-amyloid peptide (Aβ) and phosphorylated tau (pTau) were identified as the major constituents of senile/neuritic plaques and intraneuronal tangles in the 1980s (Glenner and Wong, 1984; Masters et al., 1985). Soon after, Aβ and pTau antibodies were developed for the detection of these lesions and have since served as key histopathological markers for the diagnosis AD. Besides typical senile/neuritic plaque formation, as shown by the traditional silver preparations, Aβ antibodies also help to visualize amyloid deposition that appears as diffuse plaques, as well as cerebral amyloid angiopathy (CAA) and subpial amyloidosis (Allsop et al., 1986; Yamaguchi et al., 1988; Braak and Braak, 1991; Jellinger and Bancher, 1998; Braak et al., 2006). The spatiotemporal patterns of amyloid and tangle formation have been established through the examination of postmortem brains from cognitively normal and demented human subjects and adapted into the National Institutes of Health (NIH) guidelines for AD diagnosis, and which also serve as a foundation for antemortem clinicopathological investigations with novel Aβ and pTau imaging markers (Montine et al., 2012; Boluda et al., 2015; Johnson et al., 2016; Buckley et al., 2017; Grothe et al., 2017; Jack et al., 2018; Schwarz et al., 2018; Long and Holtzman, 2019; Mattsson et al., 2019).

Thal staging divides Aβ pathogenesis into five phases: (1) occurring in isocortex; (2) spreading into allocortex (entorhinal cortex, hippocampal formation, amygdala, and insular/cingulate cortices); (3) spreading into subcortical structures (striatum, basal forebrain, thalamus, and hypothalamus) and white matter; (4) involving brainstem [red nucleus, substantia nigra, reticular formation (RF), and superior and inferior colliculi]; and (5) involving pons [RF, raphe nuclei, locus coeruleus (LC)] and cerebellum (Thal et al., 2002). The above spatiotemporal order may be not consistently predictable (Serrano-Pozo et al., 2016). Therefore, the progression of β-amyloidosis has been simplified into three stages; that is, isocortical, allocortical/limbic, and subcortical (Serrano-Pozo et al., 2011). Braak NFT staging consists of six stages: (I) occurring in the transentorhinal area; (II) spreading into the hippocampal CA1 sector; (III) to the subiculum; (IV) to the amygdala, thalamus, and claustrum; (V) to the associated isocortical areas; and (VI) to the primary sensory, motor and visual areas (Braak and Braak, 1991; Braak and Del Tredici, 2014). A simplified version is also proposed, which combines the above into three stages, that is, entorhinal (including Braak stages I and II), limbic (III and IV), and isocortical (V and VI) stages (Serrano-Pozo et al., 2011, 2016).

Recently, we reported extracellular deposition of sortilin-derived C-terminal fragments in aged and AD human cerebrum (Hu et al., 2017; Xu et al., 2018). Given that they can form microscopically prominent extracellular proteopathy, we here name these *sor*tilin *fra*gment*s* as “sorfra(s).” Our initial characterization suggests that this extracellular proteopathy is associated with neuritic-like Aβ plaques, but not with diffuse plaques or subpial and vascular amyloidosis. Notably, sorfra plaques are not detectable in some commonly used transgenic mouse models of AD (i.e., 2 × FAD, 3 × FAD, and 3 × Tg-AD) or in aged non-human primates (Zhou et al., 2018). To further understand the significance of this lesion in AD pathogenesis, it is important to determine whether sorfra deposition develops with its own characteristic spatiotemporal pattern relative to that of Aβ and pTau pathologies. Therefore, in this study, we assessed Aβ, pTau, and sorfra accumulations in postmortem human brains exhibiting various (early to late) Thal Aβ phases and Braak NFT stages of neuropathology. We suggest here that sorfra deposition is primarily a cerebral plaque lesion that spreads stereotypically from the associative neocortex, to the limbic and allocortical structures, and finally to the primary neocortical areas. This pattern of pathogenic progression in cerebral functional regions appears to be consistent with the advance of clinical symptoms of AD manifested by early cognitive and late neurological dysfunction.

## Materials and Methods

### Human Brain Samples

Postmortem human brains were collected through the willed body donation program at Xiangya School of Medicine (Yan et al., 2015), with some of the donors being clinically diagnosed as demented before or at the time of hospitalization (Table 1). A total of 46 brains were assessed according to the Standard Brain Banking Protocol set by China Brain Bank Consortium (Qiu et al., 2019). Each brain was scored with a Thal phase of amyloid pathology and a Braak NFT stage of tauopathy according to the distribution pattern of Aβ and pTau immunolabeling in cerebral and subcortical structures (Braak and Braak, 1991; Thal et al., 2002; Braak et al., 2006). Following the initial pathological profiling, sections from various cerebral and subcortical regions were obtained from appropriate cases and underwent immunohistochemical and histological analysis.

**TABLE 1:** Demographic, clinical, and pathological profiles of studied subjects.

**Case no.**	**Age, y**	**Sex**	**Clinical diagnosis and cause of death**	**Postmortem delay (h)**	**Braak NFT stage**	**Thal Aβ phase**	**Sorfra plaque stage**
1	62	F	Lung cancer	16	0	0	0
2	65	M	Heart failure	30.5	0	0	0
3	65	M	Lung cancer	6	II	0	0
4	67	M	Multisystem failure	6	II	0	0
5	68	F	Astrocytoma	4.5	II	0	0
6	68	F	Coronary heart disease	6	I	0	0
7	70	M	Cardiac stroke	5.3	II	0	0
8	70	F	Pneumonia	18	II	0	0
9	70	M	Respiratory failure	8	III	1	A
10	71	M	Cerebral stroke	8	II	0	0
11	72	M	Pneumonia	26.5	I	0	0
12	72	M	Multisystem failure, demented	4.5	V	3	B
13	74	M	Multisystem failure	5	V	4	C
14	75	M	Prostate cancer	5.3	0	0	0
15	75	M	Cardiac stroke	5	0	0	0
16	75	M	Cerebral stroke, demented	12	III	1	A
17	76	M	Cardiac stroke	17	III	1	A
18	76	M	Pneumonia	20	IV	4	B
19	77	M	Multisystem failure	12	II	0	0
20	78	F	Ovary cancer	4.5	II	0	0
21	78	M	Prostate cancer	16.5	II	0	0
22	78	M	Multisystem failure, demented	18	0	0	0
23	79	M	Cerebral stroke	2	III	3	B
24	79	M	Chronic heart failure	48	III	3	B
25	80	M	Multisystem failure	6	III	4	C
26	80	M	Chronic heart failure	10	III	2	B
27	80	F	AD	22	VI	5	C
28	80	F	AD	5.5	VI	5	C
29	81	F	AD	5.5	VI	5	C
30	82	M	AD	9.5	III	4	C
31	83	M	AD	36	IV	3	B
32	85	M	Pneumonia, demented	4.5	VI	4	C
33	85	M	Astrocytoma	12	IV	4	C
34	86	M	Hypertension	6	III	2	B
35	87	M	Brain tumor	54	III–IV	2	A
36	87	M	Cerebral stroke	6	III	1	A
37	88	M	Heart failure	6	IV	4	C
38	88	F	Heart failure	9	V	4	C
39	89	M	AD	8	V	4	C
40	91	M	AD	12	VI	5	C
41	92	M	Respiratory failure, demented	5.5	V	4	C
42	95	M	Internal bleeding, demented	5	VI	5	C
43	97	M	Multisystem failure	25	III	2	A
44	99	F	Cerebral stroke	6.3	IV	5	C
45	100	F	Hypertension, demented	3	VI	4	C
46	101	M	Multisystem failure, demented	4.5	V	4	C

### Tissue Preparation

After removal from the skull, postmortem brains were bisected, with one hemibrain cut into 2-cm-thick coronal slices and frozen at −70°C (for future biochemical studies), whereas the other hemibrain was fixed in formalin for 2 weeks. Blocks (∼0.5 cm thick) from the fixed hemibrain were then prepared from the temporal lobe at the level of midhippocampus, the frontal lobe at the level of the anterior horn of the lateral ventricle (LV) and including the precentral gyrus, and the occipital lobe at the level of the posterior horn of LV. Blocks were also taken from subcortical regions including the striatum, the diencephalon with insular cortex, the amygdala complex and basal forebrain nucleus, the midbrain at the level of the substantia nigra, the pons at its middle level, the medulla at the middle level of the olive, and finally a cerebellar slice containing cortex and dentate nucleus. These blocks were then transferred into 30% sucrose in 0.1 M phosphate buffer, embedded, and then sectioned at 40 μm using a cryostat. Sections from each block were collected and placed into the wells of culture plates filled with phosphate-buffered saline (PBS, 0.01 M, pH 7.2). Each well contained four sections at an equal distance (i.e., 24 × 40 μm or ∼1000 μm). The sections were rinsed with PBS to remove the embedding medium and then stored in a cryoprotectant at −20°C before staining.

### Immunohistochemical and Histological Preparation

All sections to be stained were removed from the culture plates stored in the −20°C freezer and rinsed with PBS to remove the cryoprotective chemicals. Relevant sections were immunolabeled with the following antibodies. A monoclonal mouse anti-Aβ 6E10 antibody (1:5000, #39320; Signet Laboratories Inc., Dedham, MA, United States), a rabbit anti-pTau antibody (1:5000, T6819, Sigma–Aldrich, St. Louis, MO, United States), a rabbit antisortilin antibody targeting the intracellular C-terminal domain (1:1000, ab16640, immunogenic peptide corresponding to a.a. 800–831 of human sortilin, ab16686; Abcam Trading Shanghai Company Ltd., Shanghai, China) (Hu et al., 2017), and a rabbit anti-β-secretase 1 (BACE1) antibody (1:1000) (Zhang et al., 2009; Cai et al., 2010). For 6E10 immunolabeling, the sections were pretreated with formic acid (90%) for 1 h at room temperature. For BACE1 immunolabeling, the sections were pretreated with 50% formamide and 50% 2 × saline-sodium citrate (SSC) at 65°C for 1 h (Zhang et al., 2009).

Other than the above antigen retrieval pretreatments, all sections were treated with 5% H_2_O_2_ in PBS for 30 min and 5% normal horse serum in PBS with 0.3% Triton X-100 for 1 h. Sections were then incubated with the primary antibodies according to the above dilutions at 4°C overnight on a laboratory rotator. The next day, sections were incubated with a pan-specific biotinylated horse anti-mouse, rabbit, and goat immunoglobulin G at 1:400 for 1 h. All sections were then rinsed in PBS and then incubated with the tertiary ABC reagent at 1:400 (Vector Laboratories, Burlingame, CA, United States) for 1 h. The final immunoreactive product was visualized in 0.003% H_2_O_2_ and 0.05% 3,3’-diaminobenzidine. An additional set of sections from all the cases was stained with the modified Bielschowsky silver stain to visualize both neuritic plaques and NFTs (Mirra et al., 1991). Briefly, sections were brought into deionized water and then sensitized in 20% silver nitrate for 20 min. This was followed by impregnating the sections in the dark with freshly prepared ammoniacal silver solution for 15 min. A developer (20% citric acid, 40% formalin, and drop of nitric acid in tap water) was then added dropwise and incubated for approximately 5 min. Finally, the sections were fixed with sodium thiosulfate. Following the immunohistochemical and silver staining, sections were air dried, dehydrated with ethanol, cleared with xylene, and then coverslippered. One set of immunolabeled sections from each staining batch was counterstained with toluidine blue, to facilitate the recognition of cytoarchitecture during microscopic examination.

### Imaging and Pathological Assessments

Immunolabeled and silver-stained sections were examined using an Olympus BX51 microscope (CellSens Standard; Olympus Corporation, Shinjuku-ku, Tokyo, Japan) for the initial assessment of immunolabeling pattern. All sections were then imaged using the 20 × objective on a Motic-Olympus microscope equipped with an automated stage and imaging system (Motic China Group Co., Ltd., Wuhan, Hubei, China), which could yield a final autofocused, montaged, and magnification-adjustable image covering the entire area of a glass slide. Motic scanned images from adjacent sections with different labelings were examined comparatively from low to high magnification. Specifically, the presence, distribution, and burden of amyloid plaque pathology were cross-validated in 6E10- and BACE1-stained sections, with the latter displaying dystrophic presynaptic terminals typically arranged as rosette-like clusters in neuritic plaques (Cai et al., 2010; Yan et al., 2014). Similarly, NFT pathology was compared between both the pTau-labeled sections and silver-stained sections to assess the presence and distribution of tangles and pretangles, as well as neuritic pathology. Particular attention was paid to the anatomical regions where the difference or change in the labeling pattern is related to Thal Aβ and Braak NFT staging. Regions included isocortical, allocortical, and hippocampal subregions in the temporal lobe section; the inferior to superior regions in the frontal lobe; the primary motor and visual cortices; and various subcortical structures. The assessment was carried out on all the selected anatomical regions. This included the presence of staining, the regional and laminar distribution of staining, and the burden of staining.

### Quantitative and Statistical Analysis

We carried out quantitative analysis on the amount of sorfra plaques in selected cerebral regions to assess the spatiotemporal progression of sorfra plaques relative to Thal Aβ and Braak NFT stages. This quantification was carried out in representative limbic regions, as well as associative and primary cortical areas, including the hippocampal CA1, subiculum (Sub), inferior temporal gyrus (ITG), superior temporal gyrus (STG) in the temporal lobe; the lateral orbit gyrus [OrG(l)] and superior frontal gyrus (SFG) in the frontal lobe; and the lateral occipitotemporal gyrus [OTG(l)], area 18 and area 17 in the occipital lobe. Images from the above areas were extracted from the original Motic files at 4 × magnification and saved as TIFF documents. In each extracted microscopic field, the area occupied (%) by sorfra plaques and the total regional area (gray matter) were obtained by using the OptiQuant software (Packard Instruments, Meriden, CT, United States), as described previously (Hu et al., 2017; Xu et al., 2019). Quantitative data from individual brains were graphed according to a three-stage staging method proposed for sorfra plaque development in the present study. Statistical analyses of means were conducted using one-way analysis of variance with Bonferroni’s multiple-comparisons test, with the minimal level of significant difference set at *p* < 0.05 (GraphPad Prism 5.1, San Diego, CA, United States). The stages of sorfra plaque formation were also compared with the Thal Aβ and Braak NFT stages assessed microscopically for individual and groups of brains.

### Figure Preparation

Figures were prepared from representative cases using the Motic-scanned images from various brain regions. Thus, a global view image at 2 × (plaque immunolabeling) or 4 × (pTau immunolabeling and silver stain) magnification was prepared for a given brain region, with high-power images from representative subregions provided as additional panels to illustrate the details of labeled profiles. Figures shown as main documents are summarizations of labelings in representative sections covering multiple neuroanatomical structures, emphasizing the differences between, and the evolution of, the neuropathologies. Supplemental figures are also provided to illustrate the global view (using an atlas format), as well as enlarged views of labelings in selected regions. Schematic figures were prepared to illustrate the spatiotemporal progression of sorfra, Aβ, and NFT lesions, based on the observational and morphometric data obtained from the current study, and in reference to the original documentation of Thal Aβ and Braak NFT staging (Braak and Braak, 1991; Thal et al., 2002; Braak et al., 2006).

## Results

### Lack of Sorfra Plaques in Brains With Primary Age-Related Tauopathy

Primary age-related tauopathy (PART) refers to the occurrence of tauopathy in the absence of Aβ deposition in the human brain, which has been well documented in literature (Crary et al., 2014; Tsartsalis et al., 2018; Kim et al., 2019). In this condition, the extent of tauopathy is generally limited to early Braak NFT stages (i.e., rarely to stages V/VI). Among the brains obtained from non-demented elderly in our brain bank during the past several years, a substantial number of cases were pathologically characteristic of PART (Table 1), therefore allowing us to investigate whether sorfra plaques could develop in the brains with tauopathy alone.

**FIGURE 1:**
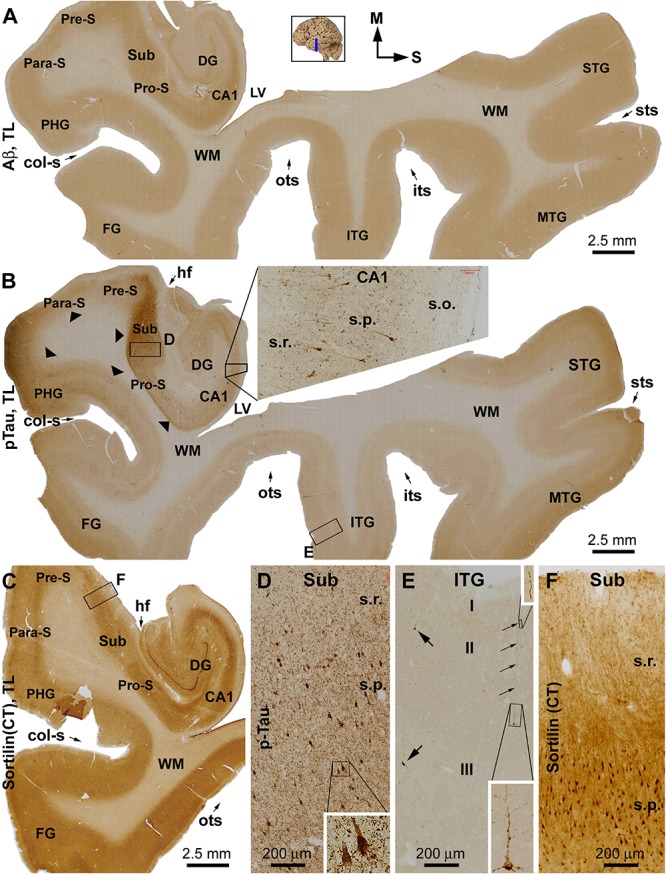
Immunohistochemical characterizations illustrating the lack of extracellular sortilin neuropathology in the human brain exhibiting primary age-related tauopathy (PART). The small whole brain map in **(A)** indicates that the sections were from the temporal lobe (TL) segment marked with a blue line (the same applies to other figures). Section orientation is indicated next to this map, with “M” toward medial and “S” toward superior in reference to anatomical position. Figure panel arrangement, neuroanatomical structures, cortical lamination, and scale bars are as indicated. Shown are TL sections immunolabeled for β-amyloid (Aβ) with the 6E10 monoclonal antibody **(A)**, phosphorylated tau (pTau) with a polyclonal rabbit antibody **(B)**, and sortilin with a rabbit antibody against the C-terminal (CT) domain **(C)**. Enlarged views from the low-magnification images **(A–C)** are shown as inserts and panels as indicated **(B,D–F)**. No extracellular Aβ deposition exists in the cortical regions and subregions of the hippocampal formation **(A)**. Intensive pTau immunoreactivity (IR) is seen in hippocampus and transentorhinal areas, including CA1, prosubiculum (Pro-S), subiculum (Sub), presubiculum (Pre-S), and parasubiculum (Pare-S), whereas the IR is reduced as moving from the parahippocampal gyrus to the neocortical gyri **(B)**. At high magnifications, pTau IR distinctly visualizes the somata and dendritic processes of a subset of pyramidal neurons in CA1 (insert in **B**) and Sub **(D)**, whereas numerous fine neuronal processes are present in the background **(D)**. In the neocortex, individual pyramidal-like neurons are visualized, with the IR filling up the somata and dendritic tree **(E)**. Sortilin IR is exclusively cellular in the cortex and hippocampal formation **(C)**, localizing to neuronal somata and dendritic processes **(F)**. The regional distribution of pTau IR is consistent with a Braak stage III scoring of neurofibrillary tangle (NFT) pathology. Abbreviations: STG, MTG, and ITG: superior, middle, and inferior temporal gyri; FG: fusiform gyrus; DG: dentate gyrus; sts, its, and ots: superior, inferior, and occipitotemporal sulci; col-s: collateral sulcus; hf: hippocampal fissure; LV: lateral ventricle; WM: white matter; s.o.: strata oriens; s.p.: strata pyramidale; s.r.: strata radiatum; s.l.m.: stratum lacunosum-moleculare.

Overall, sorfra plaques were not found in aged human brains with PART in this present study. Figure 1 shows images of adjacent temporal lobe sections immunolabeled with 6E10, pTau, and the C-terminal sortilin antibodies. 6E10 immunoreactivity appeared as diffuse background labeling without any localized extracellular Aβ deposition across the entire section covering the temporal neocortex, entorhinal cortex, and the hippocampal formation (Figure 1A). The lack of Aβ plaque pathology was confirmed by the absence of BACE1-labeled dystrophic neurites across the cortical and hippocampal subregions, whereas the distinct BACE1 expression normally present at the mossy fiber terminals was clearly seen in CA3 and the hilus of dentate gyrus (DG) (Supplementary Figure S1). Consistent with a Braak stage III scoring, there was significant pTau immunoreactivity in the subiculum and extended from the prosubiculum (Pro-S) to the entire CA1 sector and also from the presubiculum (Pre-S) to the entire parahippocampal gyrus (PHG). Less pTau-labeled neurons existed in the fusiform gyrus (FG), whereas only individual neurons (with distinctly visualized somata and dendritic processes) were detectable in more superior (or dorsal) temporal neocortical areas (Figures 1B,D,E). The C-terminal sortilin antibody visualized cellular labeling in all the temporal lobe structures, with the somata and dendrites of cortical and hippocampal pyramidal neurons, and dentate granule cells clearly displayed (Figures 1C,F). Thus, this exclusive neuronal labeling was consistent with the normal expression pattern of the full-length sortilin protein as characterized in the adult human brain (Hu et al., 2017; Xu et al., 2019).

### Early Occurrence of Sorfra Plaques in the Basal Cerebral Neocortex

**FIGURE 2:**
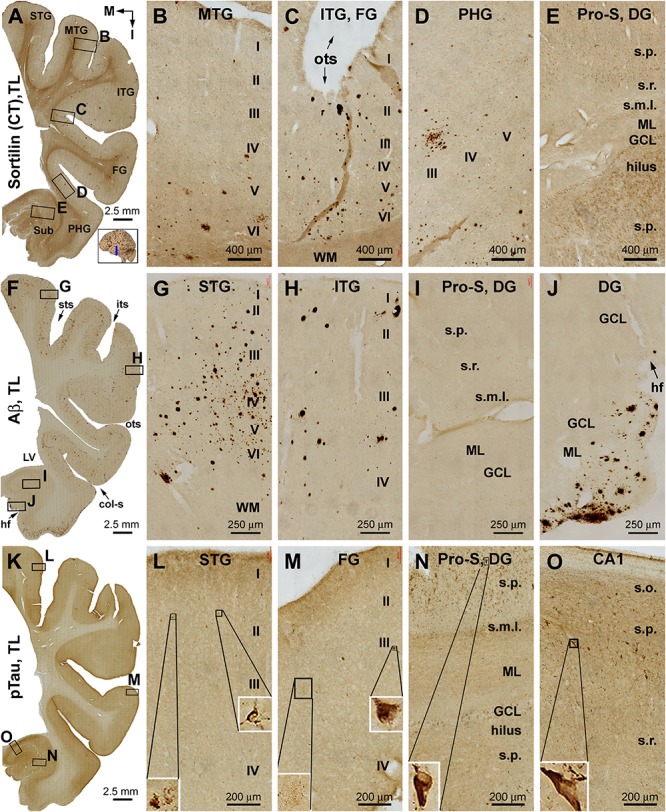
Distribution of extracellular plaques enriched of sortilin C-terminal fragments (shortened as “sorfra plaques”) in the temporal lobe structures from a brain with Aβ pathology scored at Thal phase 1 and tauopathy scored at Braak stage III. Section orientation (in **A**, with “M” points to medial and “I” points to inferior relative to anatomical position), figure panel arrangement, neuroanatomical structures, cortical lamination, and scale bars are as indicated. **(A–E)** Low- and high-power views of plaque-like and cellular labeling visualized by the C-terminal antibody. Sorfra plaques are present with an overall low density across the neocortical regions from the superior temporal gyrus (STG) to the parahippocampal gyrus (PHG) **(A–D)**. Sorfra plaques are reduced in number and then disappeared as moving from the neocortical to entorhinal parts of the PHG **(A,D)**. As such, no sorfra plaques are seen in the subicular subregions, hippocampus proper and dentate gyrus (DG) **(A,E)**. Labeled cellular profiles are present in the cortex and hippocampal formation, including the hilar mossy cells **(E)**. **(F–J)** Low- and high-power views of Aβ immunolabeling in an adjacent section. Aβ labeling appearing as compact and diffuse plaques occurs with comparable density over the superior, middle, and inferior temporal and fusiform gyri **(F–H)**. The amount of plaques is reduced in the PHG as moving into the entorhinal cortex **(F,I)**. However, a fairly large amount of diffuse Aβ deposition is present in the parasubiculum **(F)**. In addition, leptomeningeal β-amyloidosis occurs in the molecular layer (ML) along the ventricular edge of the DG **(F,J)**. **(K–O)** pTau labeling in another adjacent section. A few individually labeled neurons and neuritic clusters are recognizable in the neocortical regions from the STG to FG at high magnifications **(L,M)**. In comparison, a greater amount of pTau-immunoreactive neuronal somata and neuritic profiles are present over the entorhinal subregions and hippocampal CA1 sector **(K,N,O)**. Some pTau-labeled neurons are tangled, as featured by an uneven labeling in the somata and/or truncation of the dendritic tree (inserts in **L–O**). Abbreviations are as defined in Figure 1.

**FIGURE 3:**
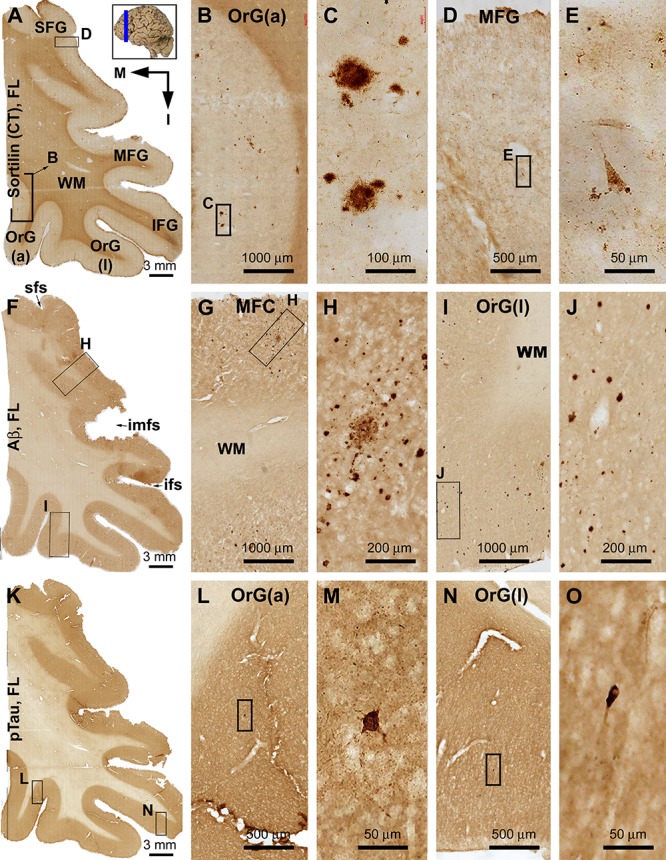
Differential distribution of sorfra plaques, β-amyloid deposition, and tauopathy in the frontal lobe from a brain with Thal phase 1 and Braak stage III neuropathologies. Section orientation (in **A**, M: medial, I: inferior), figure panel arrangement, neuroanatomical structures, and scale bars are as indicated. **(A–E)** Low- and high-power views of plaque-like and cellular labeling visualized by the C-terminal antibody. A small amount of sorfra plaques are observed in the basal cortical areas including the anterior orbit gyrus [OrG(a)] and lateral orbit gyrus [OrG(l)] **(A–C)**. In other parts of the frontal lobe including the inferior, middle, and superior frontal gyri (IFG, MFG, SFG), no sorfra plaques are recognizable, whereas labeled neuronal somata are present **(A,D,F)**. **(F–J)** Low- and high-power views of Aβ immunolabeling in an adjacent section. Compact and diffuse plaques are present in all cortical gyri with a comparable density (fairly low) **(G–J)**. **(K–O)** pTau immunolabeling in other neighboring sections. Only a few individual immunoreactive neurons are identifiable by close examination of the image at high magnification, which are located in the basal part of the frontal lobe **(L–O)**. Abbreviations: sfs, superior frontal sulcus; imfs, intermediate frontal sulcus; ifs, inferior frontal sulcus; WM, white matter.

**FIGURE 4:**
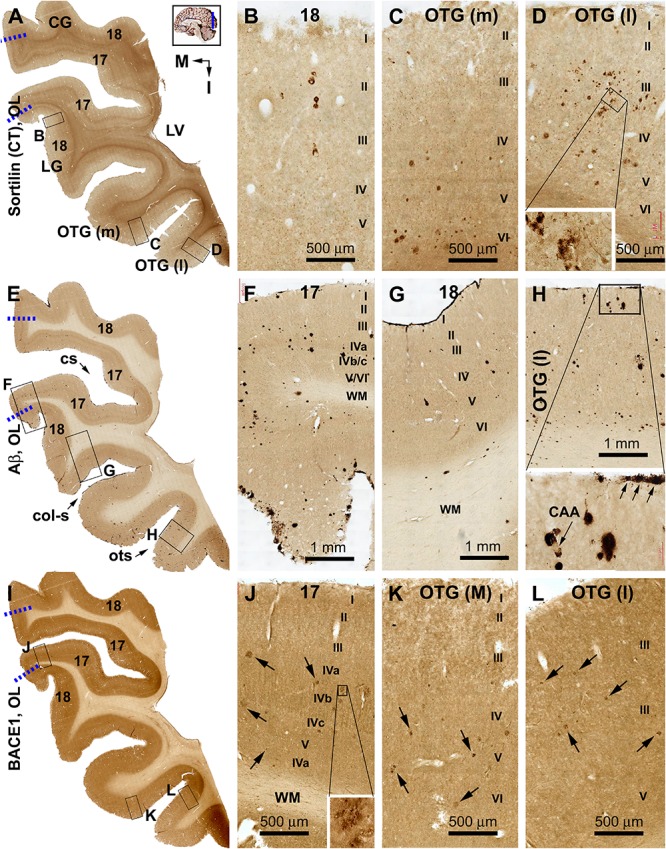
Distribution of sorfra plaques, β-amyloid deposition, and β-secretase 1 (BACE1)-labeled dystrophic neurites in frontal sections of the occipital lobe (OL) in a brain with Thal phase 1 and Braak stage III neuropathologies. Section orientation (in **A**, M: medial, I: inferior), figure panel arrangement, neuroanatomical structures, and scale bars are as indicated. The broken blue lines mark the border between areas 17 and 18. **(A–D)** Low- and high-power views of sorfra plaques. Sorfra plaques are mostly localized to the basal cortical areas including the lateral and medial occipitotemporal gyri [OTG(l), OTG(m)] and occasionally found in area 18 **(A–D)**. Only several plaques could be identified across the entire area 17 by scanning the image at high magnification. Labeled neuronal somata are seen at closer examination (**D**, insert). **(E–H)** Low- and high-power views of Aβ immunolabeling in an adjacent section. Compact and diffuse plaques are present in all cortical gyri with a comparable density **(G–J)**. Cerebral amyloid angiopathy (CAA) and meningeal Aβ deposition are found at some locations (H and enlarged insert). **(I–L)** The distribution and morphology of BACE1 immunoreactive dystrophic neurites, which are all found in the cortex of all gyri with comparable density. At high magnifications, these dystrophic neurites occur as isolated spherical profiles or rosette-like clusters (**J–L**, examples are pointed by arrows). Abbreviations: CG, cuneate gyrus; LG, lingual gyrus; LV, lateral ventricle; WM, white matter; cs, calcarine sulcus; col-s, collateral sulcus; ots, occipitotemporal sulcus; I–VI, cortical layers.

To assess the early development of sorfra plaques and the pattern of distribution, we examined sections from aged brains with early stages of Aβ and pTau pathology. Overall, less sorfra plaques were found relative to Aβ deposition in selected cerebral cortical areas in the cases with Thal phase 1 pathology, in which the NFT pathology ranged from Braak stage I–III; that is, involving the entorhinal to limbic regions as assessed with pTau immunolabeling and silver stain. The regional pattern and extent of pathology in the temporal, frontal, and occipital lobe structures from a representative case are described below with detailed illustrations (Figures 2–4) and supplemental figures (Supplementary Figures S2–S4).

#### Temporal Lobe

The C-terminal sortilin antibody visualized a small amount of extracellular lesion in the temporal neocortical areas (Figures 2A–E and Supplementary Figure S2.1). Sorfra plaques were distributed in the cortex of the STG, middle temporal gyrus (MTG), ITG, and FG. Sorfra plaques became reduced in number in the FG and essentially disappeared in the PHG. No sorfra plaques were found in the subicular subregions and the entire hippocampal formation. While scanning across the temporal neocortical regions, it appeared that sorfra plaques were distributed more frequently in the deep than upper cortical layers (Figures 2A–E and Supplementary Figures S2.1A–I).

In comparison, Aβ labeling was present across the neocortical regions from the STG, MTG, and ITG to FG (Figures 2F–J and Supplementary Figure S2.2). β-Amyloid deposition in these areas appeared largely as compact-like plaques, while lightly stained diffuse lesions also existed, as were occasionally seen vascular deposition (Figures 2G,H). β-Amyloid deposition was present in the parasubiculum (Para-S) and Pre-S over layer II. However, there was no Aβ deposition in the Sub to hippocampal CA sectors and the DG. Notably, subpial Aβ deposition occurred at the ventricular edge of the molecular layer (ML) of the DG (Figures 2F,J and Supplementary Figures S2.2A–I). pTau-labeled neurons and neuritic processes were sparsely seen in the STG to FG (Figures 2K–M and Supplementary Figure S2.3). An increased amount of pTau-positive neurons and neurites occurred from the Sub, Pro-S, to CA1, with some dentate granule cells and hilar cells also lightly labeled (Figures 2K,N,O). A subset of pTau-labeled neurons in the entorhinal cortex and hippocampal formation appeared tangled, featured by uneven and reduced somal labeling, deformed somal shape, and a truncated appearance of the dendritic arbor (Figures 2L–O, inserts and Supplementary Figures S2.3B–J). BACE1 immunolabeling was used to cross-validate the differential distribution of Aβ deposition in the temporal lobe structures (Supplementary Figure S2.4). Consistent with the regional distribution of the compact-like Aβ plaques, BACE1-labeled dystrophic neurites, arranged as clusters and occurred as isolated swollen sphericles and processes, were present in the temporal neocortical regions (Supplementary Figures S2.4A–G). However, these neuritic pathologies were not present in the subicular subregions and the hippocampal formation (Supplementary Figures S2.4A,H,I).

#### Frontal Lobe

Sorfra plaques were observed in the basal or inferior frontal neocortical gyri, with a higher density in the anterior [OrG(a)] than in lateral [OrG(l)] orbit gyri (Figures 3A–C and Supplementary Figures S3.1A,B). No plaque profiles were detectable in the inferior frontal gyrus (IFG), middle frontal gyrus (MFG), and SFG (Figures 3A,D,E and Supplementary Figure S3.1A). Labeled neuronal somata were seen in all regions at high magnifications (Figures 3D,E and Supplementary Figure S3.1C).

In comparison, Aβ plaques occurred with an overall low density across the basal as well as the lateral and superior frontal gyri, appearing largely as compact plaques localized to the middle layers of the cortex (Figures 3F–J and Supplementary Figures S3.2A–E). However, at high magnification, Aβ deposition also occurred as miniplaques and diffuse plaques, and as segmental subpial lesion. There was no apparent difference in the overall amount of Aβ deposition between the different aforementioned frontal gyri. In adjacent sections immunolabeled for pTau, only a few labeled neurons were encountered while scanning over the entire region at high magnification (Figures 3K–O). Specifically, they were found in the OrG(l), OrG(a), and IFG, but not in the MFG and SFG. Among some of these individually labeled neurons, pTau reactivity displayed the somata, as well as the apical dendritic arbor to its fine branches (Supplementary Figures S3.3A,D).

#### Occipital Lobe

Sorfra plaques occurred with an overall low density across the entire occipital cortical subregions, but localized primarily to the basal or temporal cortical regions (Figures 4A–D and Supplementary Figures S4.1A–J). Thus, small- and large-sized sorfra plaques were seen with a high to low gradient in numerical density from the ITG to the OTG(l) and further to the medial occipitotemporal gyrus [OTG(m)]. A few plaques were observed in the secondary visual cortex (i.e., Brodmann area 18), whereas virtually none were seen in the primary visual cortex (area 17) while scanning across these areas at high magnification (Figures 4A–D and Supplementary Figures S4.1A–F).

In comparison, Aβ labeling appearing predominantly as compact plaques was present in low density over the basally located occipitotemporal gyri, as well as medially and superiorly located visual cortical areas (Figures 4E–H and Supplementary Figures S4.2A–I). CAA and meningeal and diffuse Aβ labeling in the white matter were also found in the occipital lobe. The overall amount of Aβ deposition appeared to be slightly denser in the medial OTG(m) and OTG(l) in comparison with visual cortical areas. In addition, the density of Aβ plaques was noticeably higher in area 17 than in area 18 (Supplementary Figure S4.2A). Consistent with the regional distribution of Aβ plaques, BACE1-labeled dystrophic neurites arranged as rosette-like clusters and isolated profiles were present in low density in the temporal as well as visual subareas including area 17 (Figures 4I–L and Supplementary Figures S4.3A–J). In contrast to the above markers, no pTau-labeled perikaryal or neuritic profiles were found across the occipitotemporal and visual cortical regions (Supplementary Figures S4.4A–I).

### Expansion of Sorfra Plaques Over the Limbic Structures and Associative Neocortex

To explore the regional progression of sorfra plaques beyond the above early stage, cerebral and subcortical sections from brains with Thal Aβ (2–4) and Braak NFT (III–V) stages were examined. The distribution and amount of sorfra, Aβ, and pTau pathologies were compared using the Motic scanned images from adjacent sections of the temporal, frontal, and occipital lobes. Again, for the purpose of cross-validation, BACE1 and silver stain in neighboring sections were examined. The results described below were obtained from the brain of a demented patient, with Aβ and pTau pathologies at Thal phase 4 and Braak stage IV, respectively.

#### Temporal Lobe

**FIGURE 5:**
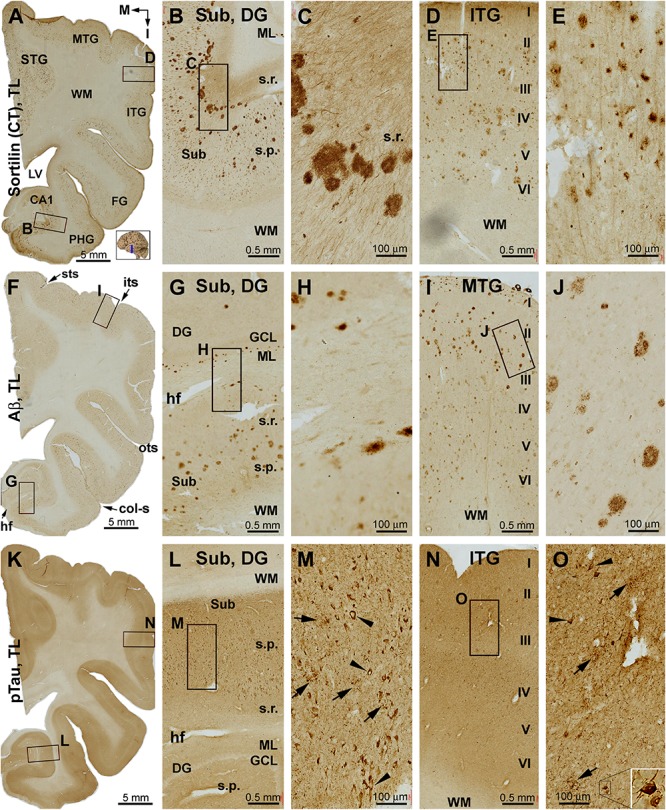
Distribution of sorfra plaques, β-amyloidosis, and tauopathy in the temporal lobe structures in a brain with Thal phase 4 and Braak stage IV neuropathologies. Section orientation, figure panel arrangement, neuroanatomical structures, and scale bars are as indicated. **(A–E)** Low- and high-power views of sorfra plaques. They are found frequently in the neocortical, entorhinal, and hippocampal subregions. The density of these plaques is comparable among the temporal neocortical gyri. Heavily stained plaques are present from the Pre-S, Sub, and Pro-S, to CA1. Sorfra plaques are also seen in the DG. They arrange as a row in the stratum radiatum (s.r.) of the Sub and the molecular layer (ML) of the DG **(A–C)**. Labeled neuronal somata and dendritic processes are seen at high magnifications **(D,E)**. **(F–J)** Aβ immunolabeling in a neighboring section. Note that the distribution and density of β-amyloid plaques appear comparable to that of sorfra plaques. However, diffuse parenchymal, vascular, and meningeal amyloidosis are visualized in Aβ labeling. **(K–O)** pTau immunolabeling in another neighboring section. Phosphorylated tau-positive neuronal somata are densely packed in CA1 and subicular subregions and frequently observed in the neocortical areas **(L–O)**. Most of these neurons appear tangled (**M,O**, examples are pointed by arrowheads and shown with enlarged insert). Abbreviations are as defined in Figure 1.

A large number of sorfra plaques existed across the temporal neocortical regions and also over the entorhinal cortical areas and all subregions of the hippocampal formation (Figures 5A–E and Supplementary Figures S5.1A–D). Thus, the overall amount of sorfra plaques increased relative to the aforementioned initial stage, together with a remarkable regional expansion. The overall density and laminar distribution of sorfra plaques appeared comparable from the STG, MTG, and ITG to FG and to the neocortical part of the FG. In comparison, sorfra plaques were reduced in number in the entorhinal part of the FG, particularly in the Para-S and Pre-S. As moving medially, they increased in the Sub, Pro-S, and CA1, wherein the plaques occurred in the strata pyramidale (s.p.) and along the strata radiatum (s.r.) in a row. Sorfra plaques were also present in the DG, with those in the ML arranged in a row (Figures 5B,C and Supplementary Figures S5.1A,D). In general, sorfra plaques were localized to the gray matter, but not to the white matter, and showed a large extent of variability in shape, size, and labeling intensity (Figures 5C,E and Supplementary Figures S5.1B–D).

Large amounts of Aβ labeling were also present across the neocortical and entorhinal cortical areas, and the hippocampal formation (Figures 5F–J and Supplementary Figure S5.2). The overall amount and distribution pattern of Aβ plaques appeared to be comparable to those of the aforementioned sorfra plaques, including the presence of plaques in the entorhinal subregions and hippocampal formation. However, Aβ labeling additionally occurred in layer II of the entorhinal cortex as typical diffuse deposition, in the subpial area along layer I, around the ventricular edge of the DG, and in the white matter as diffuse lesion or at vascular walls as CAA (Figures 5F–J and Supplementary Figures S5.2A–D). pTau immunolabeling was also abundant in all anatomical structures of the temporal lobe (Figures 5K–O and Supplementary Figures S5.3A–D). pTau-labeled neuronal and neuritic profiles were present in the temporal neocortical regions as well as the entorhinal cortical areas. Specifically, pTau reactivity was increased in the entorhinal regions extending into the hippocampal formation, with labeled neurons in the hilar part of CA3 (Figures 5K,L,N and Supplementary Figure S5.3A). At higher magnification, many pTau-labeled neurons appeared as tangles as seen from the reduced immunoreactivity and presence of thread-like elements in the somata, and truncated or dysmorphic dendritic morphology (Figures 5M,O and Supplementary Figures S5.3B–D, inserts). In agreement with the overall regional distribution of Aβ labeling, BACE1-labeled dystrophic neurites were present in the temporal neocortical areas (STG, MTG, ITG, FG, and superior half of the PHG; Supplementary Figure S5.4). The amounts of neuritic profiles were reduced in the Para-S and Pre-S, but were increased from the Pre-S toward CA1. Neuritic clusters, spherites, or processes with distinct BACE1 labeling were clearly present in the subicular lamina (s.p., s.r.) and the ML of DG (Supplementary Figures S5.4A,D). In support of the NFT pathology seen in pTau immunolabeling, tangled neurons and neuritic plaques were visualized in Bielschowsky silver sections of temporal lobe (Supplementary Figures S5.5A–D). Overall, the distribution pattern of silver-stained neuritic plaques over the cortical and hippocampal structures (Supplementary Figure S5.5A) was comparable to that of the sorfra plaques (Supplementary Figure S5.1A), Aβ plaques (Supplementary Figure S5.2A), and BACE1 dystrophic neurites (Supplementary Figure S5.4A), when examined across the Motic-scanned images of the temporal lobe region by region.

#### Frontal Lobe

**FIGURE 6:**
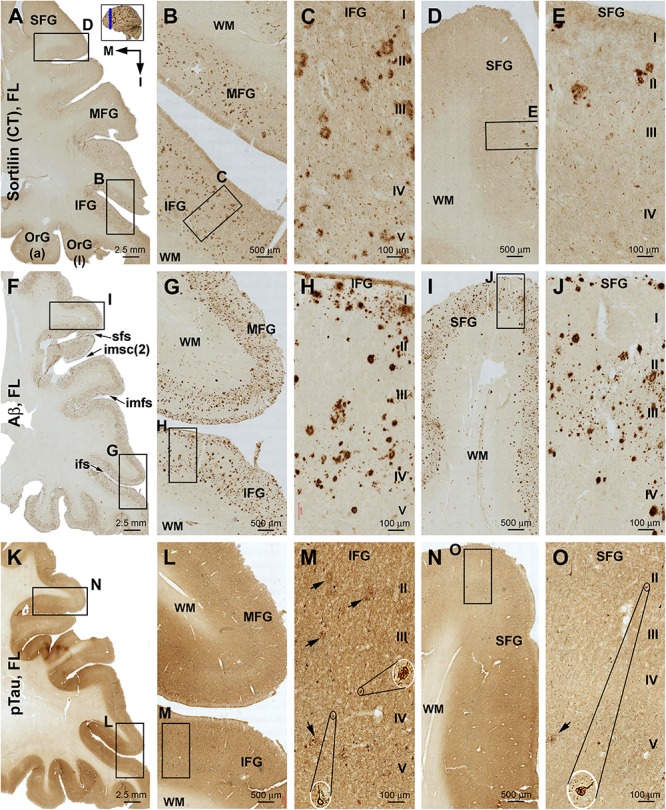
Comparison of sorfra plaques, β-amyloidosis, and tauopathy in the frontal lobe regions in a brain with Thal phase 4 and Braak stage IV neuropathologies. Section orientation, figure panel arrangement, neuroanatomical structures, and scale bars are as indicated. **(A–E)** Low- and high-power views of the distribution and morphology of sorfra plaques as oriented. There is a high-to-low gradient in the amount or density of sorfra plaques as moving from the basal/inferior to lateral/superior frontal lobe gyri **(A,B,D)**. At high magnification, sorfra plaques as well as sortilin-expressing neuronal profiles are present in the cortical gray matter **(C,E)**. **(F–J)** Aβ immunolabeling in a neighboring section. The cortex is packed with β-amyloid plaques that appear to be comparable in numerical density over the basal and lateral frontal gyri. β-Amyloid deposition also occurs in some areas of the white matter as diffuse lesions, along the pia mater and layer I especially the low portion, and also at some vascular sites **(G,H)**. **(K–O)** pTau immunolabeling in another neighboring section. Similar to the distribution pattern of sorfra plaques and different from that of Aβ deposition, pTau-positive neuronal somata and neurites are more densely distributed in the orbital gyri and the inferior frontal gyrus than in the middle and superior frontal gyri **(L–O)**. At high magnification, most pTau-immunoreactive neurons appear tangled (**M,O**, examples are shown with enlarged insert). Phosphorylated tau-immunolabeled dystrophic neurites can also be densely packed as clusters (**M,O**, pointed by arrows). Abbreviations are as defined in Figure 3.

Sorfra plaques were present across the laterally located SFG, MFG, and IFG and the basally located OrG(l) and OrG(a) (Figures 6A–E and Supplementary Figure S6.1). However, there existed a clear low to high gradient in the overall amount or numerical density of the plaques going from the SFG and MFG to IFG and OrG. Thus, compared to the amount and distribution of sorfra plaques in the frontal lobe described above at an earlier pathological stage, there existed a basal/inferior to lateral/superior expansion in regard to the distribution map (Supplementary Figures S3.1A, S6.1A).

In contrast to sorfra plaques, Aβ labeling occurred in the lateral and basal gyri of the frontal lobe with comparable density, whereas the overall amount of deposition was dramatically increased relative to the aforementioned early stage of lesion in the frontal lobe (Figures 6F–J and Supplementary Figure S6.2). Morphologically, Aβ deposition presented as a mixture of compact and diffuse parenchymal plaques and subpial and vascular amyloidosis, with diffuse deposition in the white matter (Figures 6G–J and Supplementary Figures S6.2B–J). pTau immunoreactivity was visible in all frontal cortical gyri at this stage (Figures 6K–O and Supplementary Figures S6.3A–F). However, different from the pattern of Aβ deposition but similar to that of sorfra plaques, there existed a low to high gradient in pTau labeling from the superior/lateral to inferior/medial cortical regions, along with an overall increase in the amount of labeled profiles. Thus, pTau-labeled neuronal somata and neuritic processes occurred with increasing densities as moving from the SFG and MFG to the IFG and OrG (Supplementary Figures S6.3A–H). In BACE1 immunolabeling (Supplementary Figure S6.4) and silver stain (Supplementary Figure S6.5), clusters of dystrophic neurites and neuritic plaques showed a non-differential regional distribution over the lateral and basal frontal cortical regions. The overall amounts of BACE1-labeled dystrophic neurites and silver-stained plaques were less than those of the Aβ labeling, because there existed large amounts of diffuse plaques and meningeal and vascular amyloidosis in the region in Aβ immunolabeling. Tangled neurons were found in all neocortical and limbic regions in silver stain at high magnifications (Figures 6M,O and Supplementary Figures S6.5B,G).

#### Occipital Lobe

**FIGURE 7:**
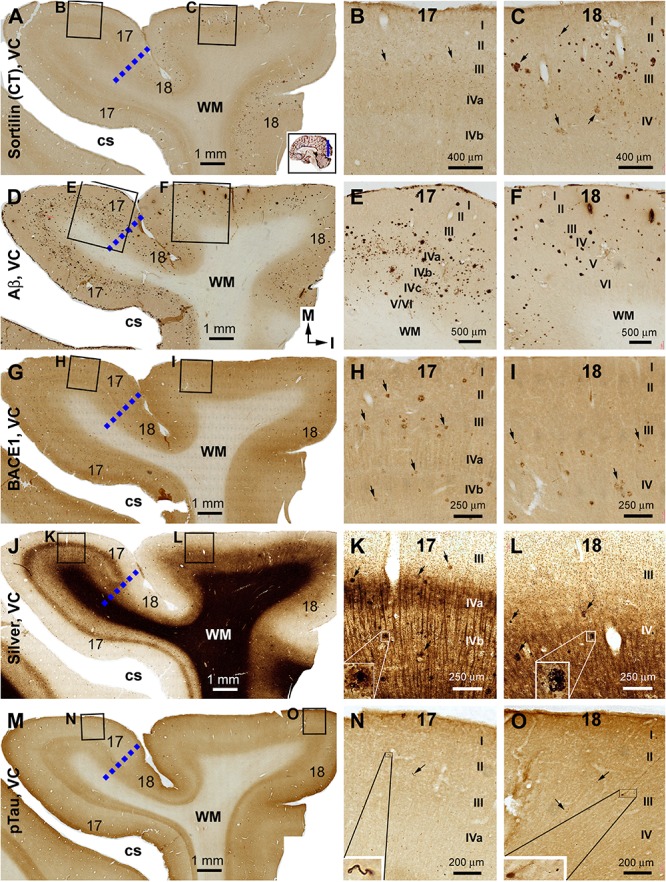
Correlative assessment of plaque and tangle pathologies using multiple markers in the visual cortex (VC) in a brain with Thal phase 4 and Braak stage IV neuropathologies. Section orientation, figure panel arrangement, neuroanatomical structures, and scale bars are as indicated. The border of areas 17 and 18 is marked by a broken blue line for each type of labeling **(A,D,G,J,M)**. **(A–C)** Low- and high-power views of the distribution and morphology of sorfra plaques in areas 17 and 18. Sorfra plaques are more densely distributed but more heavily labeled in area 18 than in area 17 (**B,C**, examples are indicated by arrows). **(D–F)** Aβ immunolabeling in a neighboring section of the VC. The amount of β-amyloid plaques appears to be greater in area 17 than in area 18. In the striate cortex, β-amyloid deposition appearing as compact and diffuse-like plaques over the sublayers of IV, whereas the lesions in area 18 occur largely as compact-like plaques **(E,F)**. **(G–I)** Low- and high-magnification images of BACE1 immunolabeling, with the pathological profiles representing dystrophic neurites arranged as clusters or isolated swollen neurites. At high magnification, the neuritic clusters are largely distributed in the deeper layers of the cortex (i.e., III–VI) in both areas 17 and 18, including the sublayers of IV in the former (**H,I**, as pointed by arrows). **(J–L)** Low- and high-magnification views of silver-stained neuritic plaques in the primary and secondary visual cortical areas. At high magnification, the neuritic clusters are largely distributed in the deeper layers of the cortex (i.e., III–VI) in both areas 17 and 18, as pointed by arrows. Silver-stained neuronal somata are rarely observed in both visual centers. **(M–O)** pTau immunolabeling in a neighboring section. There exist a few pTau-labeled neuronal somata in area 18, whereas pTau-positive neuronal processes are occasionally encountered in areas 17 and 18 by close examination of the entire section (**N,O**, inserts). Abbreviations are as defined in Figure 4.

Sorfra plaques were present in a substantial amount in area 18, whereas much fewer lesions occurred in area 17 (Figures 7A–C and Supplementary Figure S7.1). The plaques in area 18 varied in shape, size, and labeling intensity from occasional to significant, whereas those in area 17 were generally weakly stained and relatively small in size (Figures 7B,C and Supplementary Figures S7.1B–E). In comparison, Aβ labeling appearing as compact and diffuse plaques was distributed in both areas, with even a greater staining density in area 17 as against area 18 (Figure 7D and Supplementary Figure S7.2A). There existed a significant amount of diffuse-like plaques over layers IVa–IVc in area 17, whereas those in area 18 appeared predominantly as compact-like lesions (Figures 7E,F and Supplementary Figures S7.2B–E). BACE1-labeled dystrophic neuritic profiles were also present in both areas 17 and 18, again with a greater overall density in the former (Figures 7G–I and Supplementary Figures S7.3A–I). BACE-labeled neuritic clusters occurred largely in the middle part of the cortex including the widened layer IV in area 17 (Figures 7H,I and Supplementary Figures S7.3B–J). In silver stain, neuritic plaques occurred in both visual cortices, also with a noticeably greater amount in area 17 than area 18 (Figures 7J–L and Supplementary Figure S7.4). The neuritic plaques over layers IVa–c were intermingled with vertically oriented neuronal processes (likely representing the invading axonal terminals from subcortical centers) forming a distinct tangential band (Figure 7J and Supplementary Figure S7.4A). At high magnifications, some neuritic plaques had a heavily stained core (inserts in Figures 7K,L; also see Supplementary Figures S7.4C,E,G,I). Swollen neurites were seen to connect with silver-stained fine processes outside the plaques (Supplementary Figures S7.4B–I). In pTau immunolabeling, only a few labeled neurons and neuritic profiles occurred in areas 18 and 17, which were detected by scanning over the entire section at high magnifications (Figures 7M–O and Supplementary Figures S7.5A–I). The pTau-immunoreactive neurons did not appear to be tangles (Figure 7O).

### Sorfra Plaques in the Primary Neocortical Functional Areas

According to the Braak NFT staging, tauopathy in the primary motor and sensory neocortical functional areas indicates that the pathogenesis has reached stage VI. In the NIH “ABC” scoring system, the occurrence of Aβ plaques, silver-stained neuritic plaques and NFTs in areas 17 and 18 mark stage C, or the end stage of AD neuropathology (Montine et al., 2012). In the present study, no or rare sorfra plaques were observed in the primary motor (areas 4, 6) and somatosensory (areas 3, 1, 2) neocortical regions in the cases described in the preceding two result sections (data not shown). On the other hand, we have documented previously the presence of abundant sorfra plaques in the temporal neocortex, entorhinal cortex, and hippocampal formation, as well as the primary somatosensory cortex in the brains with stage C AD pathology (Hu et al., 2017). To avoid redundancy with published observations, results obtained from the primary motor and visual cortices from a brain with Thal phase 5 and Braak stage VI are shown below as representative data.

#### Primary Motor Cortex

**FIGURE 8:**
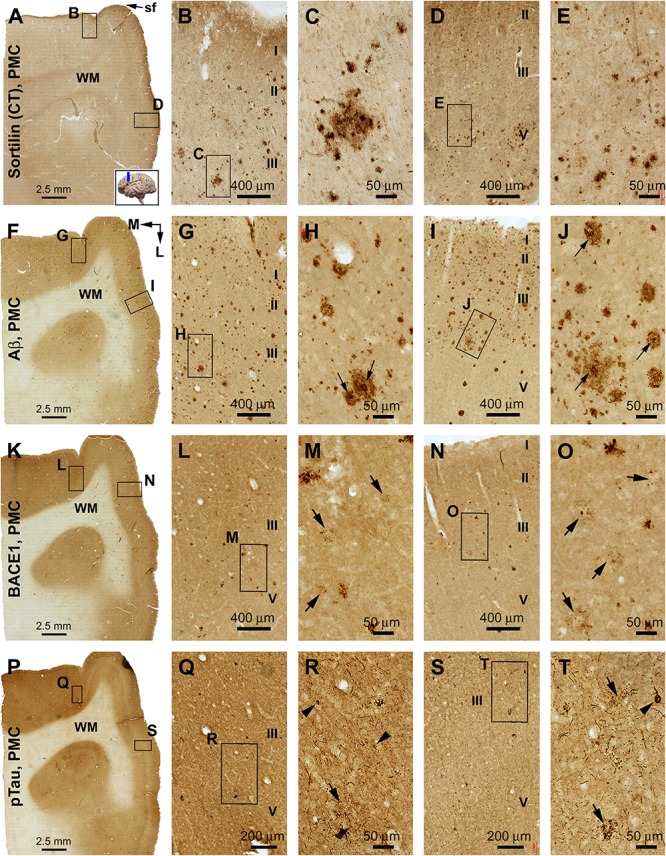
Comparison of sorfra plaques, β-amyloid, and pTau pathologies in adjacent sections of the primary motor cortex (PMC) in an AD brain with Thal phase 5 and Braak stage VI neuropathologies. Section orientation, figure panel arrangement, cortical lamination (I–VI), and scale bars are as indicated. For orientation, “sf” in **(A)** is the abbreviation of the cerebral sagittal fissure, with “M” and “L” in **(B)** indicating the medial and lateral directions relative to anatomical position. **(A–E)** Low- and high-power views of the distribution and morphology of sorfra plaques in area 4, including the medially located paracentral lobule **(A–C)** and the laterally located precentral gyrus **(A,D,E)**. Labeled extracellular plaques, as well as neuronal somata, are present in the cortical gray matter of both subregions **(B–E)**. **(F–J)** Aβ immunolabeling, which appears as densely packed compact and diffuse-like plaques over the superficial layers (I–III) of the cortex. Many compact plaques have an intensely labeled core (**H,J**, as pointed by arrows), whereas some large diffuse-like plaques also have core-like area with heavy immunolabeling (**J**, low-left). **(K–O)** BACE1-immunolabeled dystrophic neurites in both subregions of the PMC. At high magnification, some neuritic clusters consist of strongly labeled and densely packed dystrophic neurites, whereas other clusters contain loosely packed and lightly stained neuritic elements (**M,O**, as pointed by arrows). **(P–T)** The presence of pTau-immunoreactive neuronal somata and processes in both subregions of area 4. Phosphorylated tau-positive neurites are mostly present as fine processes, whereas some of them are apparently arranged as clusters (**Q–T**, as pointed by arrows). Phosphorylated tau-labeled neuronal somata often appear to be tangled by close examination (**R,T**, as pointed by arrowheads).

In both the lateral (precentral gyrus) and medial (paracentral lobule) parts of area 4, sorfra plaques occurred in a substantial amount over the cortical gray matter (Figures 8A–E). The plaque profiles showed variability in size, shape, and labeling intensity. Some large lesions consisted of regions with densely and loosely packed deposits. Labeled neuronal profiles were present between the plaques, with those in regions with no or lesser plaques (e.g., layers II/III) exhibiting more distinct somal and dendritic labeling (Figures 8B–E). In adjacent sections, Aβ immunolabeling was present in large amount and appeared as heavily stained compact and lightly stained diffuse plaques (Figures 8F–J). Typical cored plaques were seen among the compact-like plaques; however, some large diffuse-like plaques could also have a heavily stained central core (Figures 8H,J). In BACE1 immunolabeling, labeled profiles consisted of small and irregularly shaped elements at low magnification (Figures 8K–O). At high magnification, there were swollen neurites arranged as clusters occupying areas in the same range of that of compact plaques, while isolated neuritic sphericles also existed. Notably, there appeared to be a “burnout” effect among a large subpopulation of the BACE1-labeled neuritic clusters. Thus, in the same microscopic field, a small subset of clusters was composed of distinctly labeled and tightly packed swollen neurites, whereas most other clusters otherwise contained fewer, lightly or fuzzily stained, and loosely packed neuritic elements. Some severely “burnout” clusters could occupy an area (500–100 μm wide) as large as a compact plaque, whereas very few dot-like neuritic elements remained at the local site (Figures 8M,O, pointed by arrows). In another neighboring section, pTau immunolabeling was present in both the precentral gyrus and paracentral lobule of area 4 (Figures 8P–T). Thus, immunoreactive neuronal processes occurred heavily in the gray matter, while morphologically tangled neuron (arrowheads) and clusters of dystrophic neurites (arrow) were found among the labeled fine processes (Figures 8Q–T).

#### Primary Visual Cortex

**FIGURE 9:**
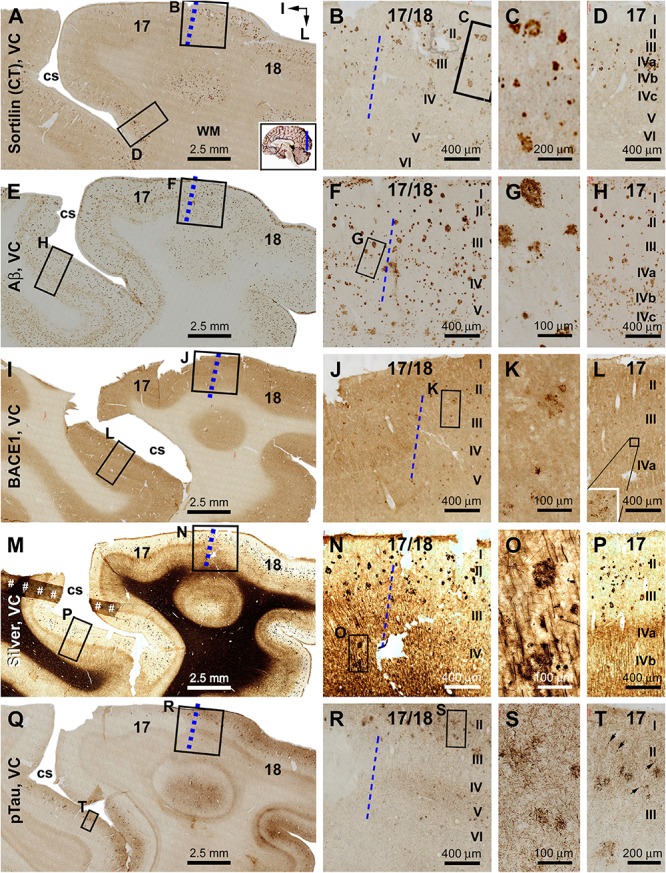
Plaque and tangle pathologies visualized by multiple labeling methods in adjacent sections of the visual cortex (VC) in an AD brain exhibited Thal phase 5 and Braak stage VI neuropathologies. Section orientation, figure panel arrangement, regional/laminar organization, and scale bars are as indicated. The blue broken lines mark the border between areas 17 and 18. Sorfra plaques are present in both areas 17 and 18, but appear more frequently in the latter **(A–C)**. They are largely located over layers II–IV, including the three sublayers of IV in area 17 **(B–D)**. **(E–H)** Aβ immunolabeling in a neighboring section, with compact and diffuse plaques densely packed in both visual cortical regions. In area 17, a large amount of β-amyloid deposition in layers IVa–IVc appear to be diffuse-like plaques, whereas the lesions in layers II–III, V, and VI in areas 17 and 18 appear to be large compact-like **(E–H)**. Leptomeningeal Aβ deposition is also evident in both regions **(A,F,H)**. **(I–L)** BACE1 immunolabeling at low and high magnifications, with the pathological profiles appearing as dystrophic neurites arranged as clusters or isolated swollen neurites located over layers II–VI **(J–L)**. **(M–P)** Low- and high-magnifications of silver-stained neuritic plaques in the primary and secondary visual cortical areas. At high magnification, the neuritic clusters are largely distributed in layers II–VI in both areas 17 and 18. In the deep cortical layers (IV–VI), silver-stained neuronal processes are present in both areas, most of which are oriented perpendicularly relative to the cortical surface **(N–P)**. **(Q–T)** pTau-labeled neuronal somata and neurites clearly present in both areas 17 and 18. The overall amount of labeled profiles appears to be greater in area 18 than in area 17, with layers II–III exhibiting a denser labeling relative to IV–VI. There is a noticeable variability in the amount of labeling across area 17, with the cortical part lying around the deeper part of the calcarine sulcus (cs) contained denser labeling relative to the part surrounding the superficial part of the cs **(Q)**. Note that this regional variability in pTau reactivity is also noticeable for the amount of sorfra plaques across area 17 **(A)**. At high magnification, pTau-labeled neuronal processes can arrange as densely packed neuritic clusters, with tangled neuronal somata distributed between the fine and swollen neuritic profiles (**T**, as pointed by arrows). Referring to Figure 4 for all abbreviations.

Relative to the distribution described in the preceding result section, sorfra plaques occurred apparently in area 17 in addition to area 18 (Figures 9A–D and Supplementary Figure S8.1A). Notably, the overall amount of sorfra plaques remained greater in area 18 than in area 17. There existed a regional variability in the amount of sorfra plaques in area 17 while examining across this cortex along the two banks of the calcarine sulcus (Figure 9A and Supplementary Figure S8.1A). Again, sorfra plaques varied greatly in size and labeling intensity, with large-sized profiles appearing circular but lacked a core structure (unlike the cored Aβ compact plaques) (Figure 9C and Supplementary Figures S8.1B–I). In Aβ labeling, the overall amount of plaques appeared to be greater in area 17 than in area 18, largely related to the presence of mostly the diffuse-like plaques over layers IVa–IVc in the former, which formed a tangential band along layer IVa (Figures 9E–H and Supplementary Figures S8.2A–I). Unlike sorfra plaques, there was no apparent regional variability in the amount of Aβ labeling as moving along the tangential direction of area 17 around the calcarine sulcus (Figure 9E and Supplementary Figure S8.2A). BACE1-labeled neuritic profiles occurred in large amount over areas 17 and 18 (Figures 9I–L and Supplementary Figures S8.3A–I). The labeled profiles were generally small in size, with a “burnout” effect seen among most of the BACE1-labeled neuritic clusters when examined at high magnifications (Figures 9K,L and Supplementary Figures S8.3C,E,G,I). In Bielschowsky silver stain, a large amount of neuritic plaques was also visualized in areas 17 and 18, with their density appearing relatively higher in area 18 than in area 17 especially in the superficial cortical layers (II–III) (Figures 9M–P and Supplementary Figures S8.4A–I). The plaques in these layers were heavily stained and often relatively large in size (Supplementary Figures S8.4B–I), whereas a “burnout” effect could be recognized based on a loss of silver stain in part of the area of a same plaque (Supplementary Figure S8.4C, arrow). A lesser amount of neuritic plaques, often relatively small in size, was localized to the deep layers (IV–VI) of the cortex wherein silver-stained bundles of neuronal processes oriented perpendicularly to the cortical surface (Figures 9N,O and Supplementary Figures S8.4E,I). pTau immunolabeling had occurred in both areas 17 and 18 at this Braak stage, with a greater density in the latter (Figure 9Q and Supplementary Figure S8.5A). Notably, the distribution of pTau labeling appeared fairly even as moving through the cortex in area 18. However, there was a regional variability in the amount of labeling in area 17 while moving through the cortex (Figure 9Q and Supplementary Figure S8.5A). Notably, the cortical regions with lesser pTau labeling matched spatially with those that exhibited fewer sorfra plaques, and vice versa (Figures 9A,Q and Supplementary Figures S8.1A, S8.5A). At high magnification, pTau-immunoreactive neurites arranged as fairly large (50–100 μm in width) clusters likely representing the sites of neuritic plaques. These neuritic clusters were mostly localized to layers II–III in both areas 17 and 18, whereas clusters in smaller size also occurred in deeper cortical layers (Figures 9Q–T and Supplementary Figures S8.5B–I). Labeled neuronal somata could be identified at high magnifications, some of which appeared tangled (Supplementary Figures S8.5B–I).

### Absence of Sorfra Plaques in Subcortical Structures

**FIGURE 10:**
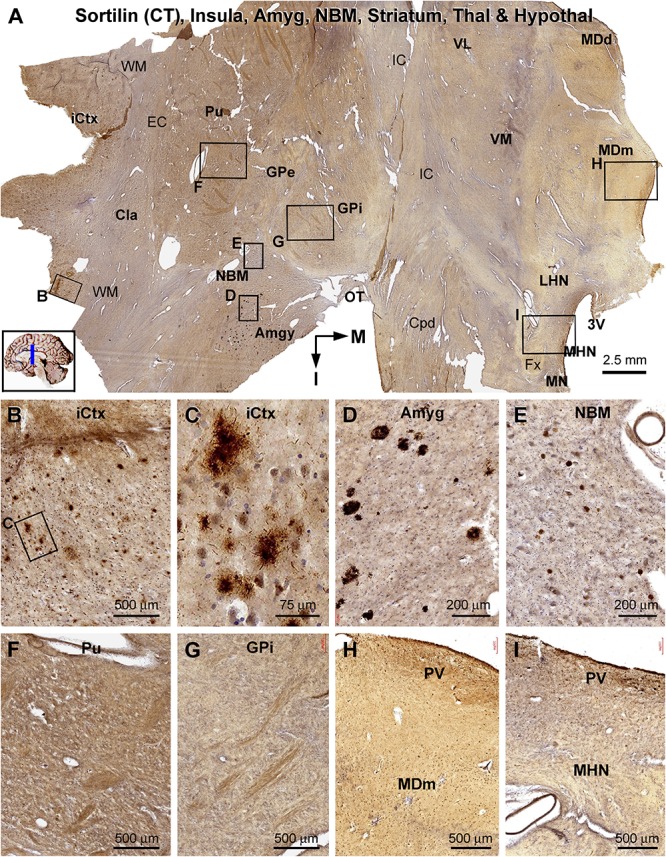
Lack of sorfra plaques in the striatum and diencephalon in an AD brain exhibited Thal phase 5 and Braak V stage neuropathologies. Section orientation, anatomical regions, figure panel arrangement, and scale bars are as indicated in individual panels. **(A)** A low-magnification image of the immunolabeled section (counterstained with toluidine blue) at the level of the mammillary nucleus (MN), displaying sortilin C-terminal (CT) antibody labeling in multiple telecephalic and diencephalic structures. Framed areas in **(A)** are enlarged and shown as **(B–I)**. Sorfra plaques are present in the insular cortex (iCtx) **(B,C)** and the amygdaloid complex (Amyg) **(D)**. Large neuronal somata are labeled in the nucleus basalis of Meynert (NBM), while no plaque profiles are seen in this basal forebrain nucleus **(E)**. There are also no sorfra plaques present in the claustrum (Cla), and all the striatal, thalamic, and hypothalamic subregions, such as the putamen (Pu) **(F)**, the external (GPe) and internal (GPi) divisions of the globus pallidus (GP) **(A,G)**, the dorsal (MDd) and medial (MDm) divisions of the mediodorsal nucleus (MD) of thalamus **(A,H)**, and the lateral (LHN) and medial (MHN) hypothalamic nucleus (HN) **(A,I)**. Abbreviations: EC, external capsule; Fx, fornix; IC, internal capsule; VL, ventrolateral thalamic nucleus; VM, ventromedial thalamic nucleus; Cpd, cerebral peduncle; OT, optic tract; 3V, third ventricle; PV, paraventricular nucleus; WM, white matter.

**FIGURE 11:**
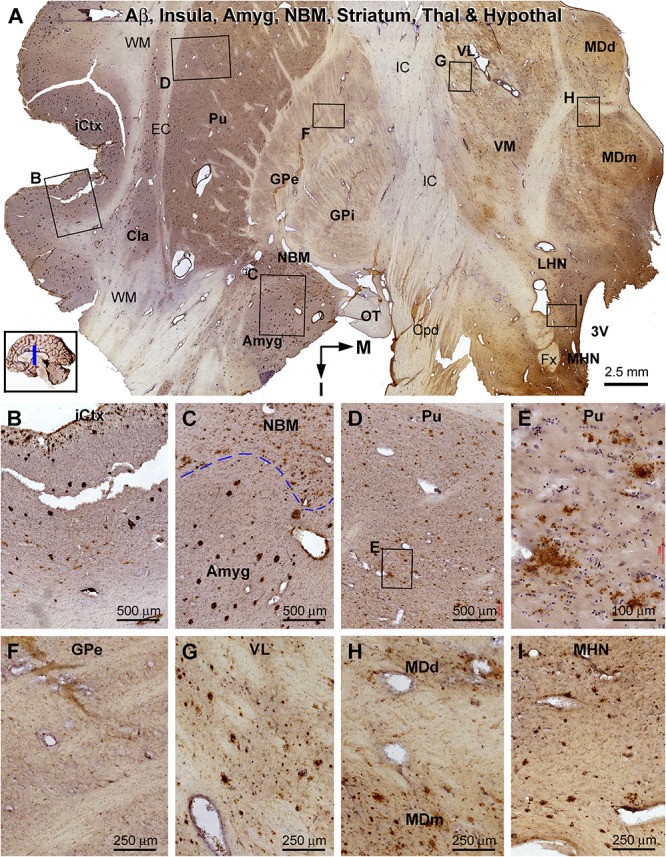
Distribution of β-amyloid deposition in the cerebral, striatal, and diencephalic subregions in an AD brain exhibited Thal phase 5 and Braak V stage neuropathologies. Section orientation, anatomical regions, figure panel arrangement, and scale bars are as indicated. **(A)** The low-magnification view of Aβ immunolabeling (with toluidine blue counterstain) over the entire section, with individual framed areas enlarged as **(B–I)**. β-Amyloid deposition in the insular cortex and claustrum (Cla) appears as both compact and diffuse-like plaques **(A,B)**. In the amygdala, Aβ plaques appear to be predominantly the compact type, whereas those in the neighboring NBM are manifested as the diffuse-type deposition **(C)**. In the striatum, large amounts of diffuse type Aβ plaques are present in the putamen (Pu) **(D,E)**, whereas little Aβ deposition exists in the globus pallidus (GP) **(A,F)**. Diffuse-like Aβ deposition exists in all thalamic and hypothalamic subdivisions, as shown in enlarged views of the ventrolateral thalamic nucleus **(A,G)**, mediodorsal thalamic nucleus **(A,H)**, and lateral and medial hypothalamic nuclei **(A,I)**. Abbreviations are as defined in Figure 10.

**FIGURE 12:**
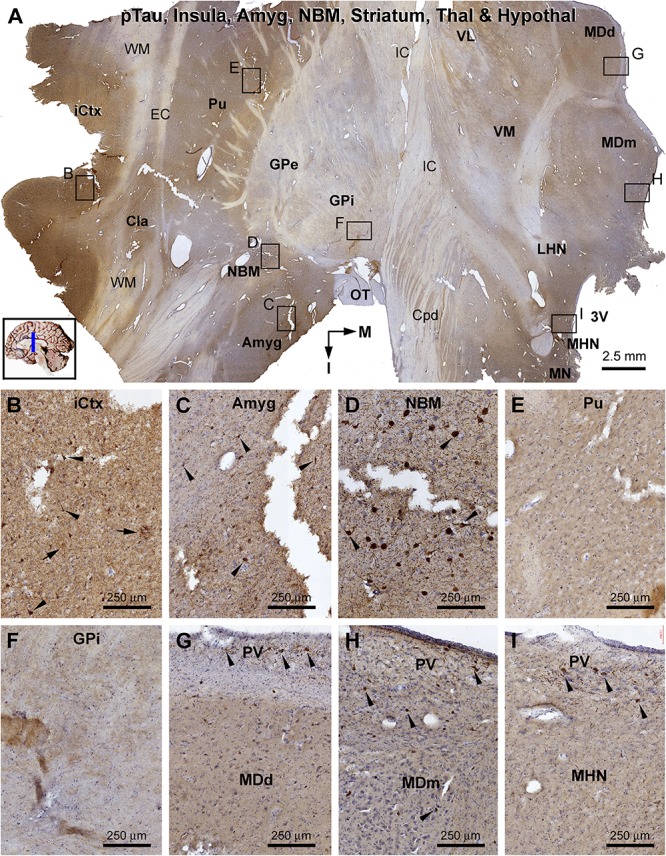
Distribution of pTau-immunoreactive neuronal profiles in the cerebral, striatal, and diencephalic subregions in an AD brain exhibited Thal phase 5 and Braak V stage neuropathologies. Section orientation, anatomical regions, figure panel arrangement, and scale bars are as indicated. **(A)** A whole section view of pTau immunolabeling (with toluidine blue counterstain) at low magnification, with the framed areas enlarged and shown as **(B–I)**. Phosphorylated tau-immunoreactive neuronal profiles and neuritic clusters are present in the insular cortex **(A,B)**, claustrum **(A)**, and amygdala **(A,C)**, with some labeled neurons appeared to be tangled (as pointed by arrows). In the NBM, large-sized putative cholinergic neurons exhibit strong IR (as pointed by arrows), whereas neuritic clusters are rarely found **(D)**. In both the putamen and GP, neither labeled neuronal nor neuritic profiles are visualized **(E,F)**. In the thalamus and hypothalamus, a small number of pTau-immunoreactive neurons (as pointed by arrows) are found, mostly localized to the paraventricular (PV) region **(G–I)**. Abbreviations are as defined in Figure 10.

Following examination of sections from multiple cases at various Thal Aβ and Braak NFT stages, we did not find a presence of sorfra plaques in subcortical structures. Results detailed below were obtained from a brain with Thal Aβ 5 and Braak V stage and showed a lack of sorfra plaques in the striatum and diencephalon with extensive Aβ and pTau pathologies (Figures 10–12). Other subcortical data will be briefly described, with figures provided as Supplementary Material (Supplementary Figures S9–S12).

In the frontal cerebral sections at the level of the mammary bodies, immunolabeled profiles over multiple telecephalic and diencephalic structures were visualized (Figures 10–12). Sorfra plaques were found in only two regions, the insular cortex and the amygdaloid complex (Figures 10A–I). The plaques in the insular cortex exhibited morphological variation in terms of size, shape, and labeling intensity (Figures 10B,C), whereas the plaques in the amygdala were mostly densely packed, round or oval in shape, and in varying size (but <100 μm in diameter) (Figure 10D). In the neighboring nucleus basalis of Meynert (NBM), there were no sorfra plaques, whereas large-sized neuronal perikarya representing cholinergic neurons were labeled (Figures 10A,E). No sorfra plaques were detected in the claustrum (Cla), and all the striatal, thalamic, and hypothalamic subregions (Figures 10A,F–I). White matter and neuroanatomic fiber tracts (e.g., external and internal capsules, cerebral peduncle, fornix, and optic tract) showed no immunolabeling (Figures 10A,F–I).

β-Amyloid deposition was broadly present in the cerebral and subcortical structures (Figure 11). In the insular cortex, Aβ labeling appeared as compact and diffuse plaques, along with meningeal and vascular lesions (Figures 11A,B). β-Amyloid deposition in the amygdaloid complex appeared predominantly as compact plaques, whereas that in the NBM manifested as diffuse deposition (Figures 11A,C). A large amount of diffuse Aβ plaques was present in the Cla, putamen (Pu), and all thalamic and hypothalamic nuclei, whereas fewer deposition existed in the globus pallidus (GP) especially the external subdivision (GPe) (Figures 11A,D–I). In pTau immunolabeling, neuronal profiles and neuritic clusters were observed in the insular cortex and amygdala, with some labeled neurons appearing to be tangled (Figures 12A–C). In the NBM, large-sized neurons together with their dendrites were labeled, whereas no clusters of swollen neurites were found (Figures 12A,D). No labeled neuronal or neuritic profiles were seen in the putamen and GP (Figures 12A,E,F). A small number of neurons in the thalamic and hypothalamic nuclei exhibited strong pTau reactivity, mostly localized to the paraventricular region (Figures 12A,G–I). BACE1-labeled dystrophic neurites were distinct in the insular cortex and amygdala (Supplementary Figures S9.1A–C), but were rarely detected in remaining regions mentioned above including the NBM (Supplementary Figures S9.1A,D–F). BACE1 immunoreactive normal-looking neuronal processes were found in the amygdala, both the external and internal GP, with neuropil-like reactivity in the putamen and thalamic nuclei (Supplementary Figures S9.1A,F). In silver preparation, neuritic plaques were found in the insular cortex and amygdala, while only labeled neuronal somata were present in the NBM (Supplementary Figures S9.2A–I). In other regions such as the mediodorsal thalamic nucleus, a small number of plaque-like lesions could be identified, but they lacked dystrophic neurites (Supplementary Figures S9.2E,I).

Briefly summarizing the findings from the midbrain, pons, and cerebellar sections (Supplementary Figures S10–12), Aβ labeling appearing as diffuse plaques occurred over the areas dorsal and ventrolateral to the cerebral aqueduct (periaqueductal gray, superior colliculi, and RF), pars compact substantia nigra (SNc), LC, RF, dorsal raphe (DR), and the cerebellar ML. The C-terminal sortilin antibody visualized cellular but not plaque-like profiles in the midbrain, pons, and cerebellar sections. pTau-labeled neurons were found in the SNc, LC, RF, and DR, but not in other regions. In silver stain, faintly stained small plaques could be recognized in the gray and white matter areas, but they manifested as amorphous silver staining not associated with dystrophic neurites. BACE1 immunolabeling appeared as neuropil-like labeling over the areas of gray matter regions and RF in the midbrain and pons, and in the cerebellar ML. Specifically, BACE1-labeled clusters of dystrophic neurites were rarely detected in the midbrain, pons, and cerebellum.

### Quantification of Sorfra Plaques in Selected Cerebral Subregions

**FIGURE 13:**
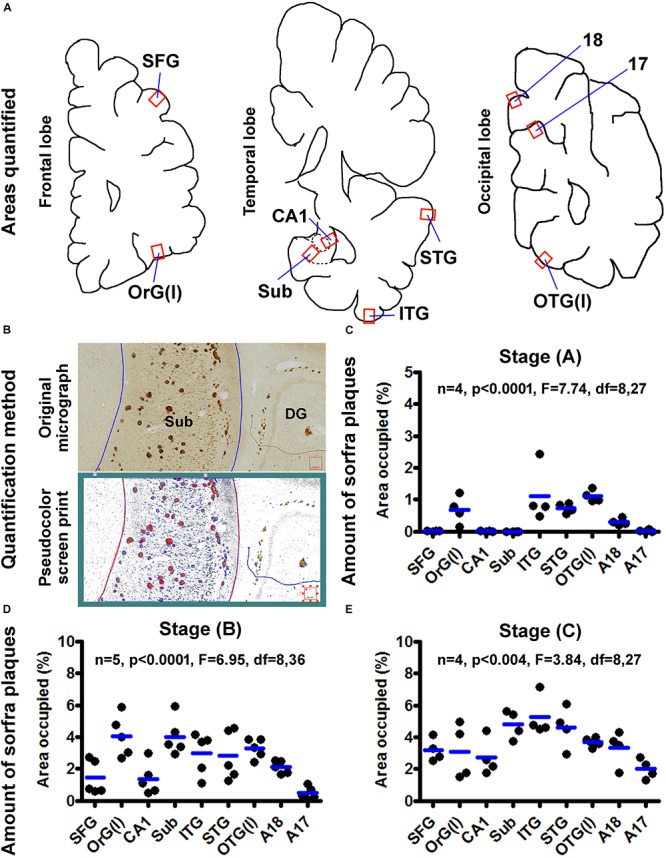
Quantification of sorfra plaques in selected cerebral subregions in support of a three-stage classification of neuropathological progression. **(A)** The neuroanatomical areas subjected to quantitative analysis, including the gyral portions of the lateral orbital gyrus OrG(l) and superior frontal gyrus (SFG) in the frontal lobe; the middle segment of hippocampal CA1, the middle segment of the subiculum and the gyral portions of the inferior (ITG) and superior (STG) temporal gyri in the temporal lobe; and the gyral portion of the lateral occipitotemporal gyrus OTG(l), the middle segment (sulcal portion) of area 17 (A17) and the gyral portion of area 18 (A18) in the cuneate gyrus in the occipital lobe. **(B)** The methodology for quantification of the ratio of area occupied by sorfra plaques relative to the total regional area, expressed as “% area occupied.” The top part is the original micrograph of immunolabeling covering the middle segment of the subiculum (Sub) from a case with stage B sorfra plaque pathology, which is converted into a black and white TIFF file (low part, pseudocolor screen print). The areas of individual sorfra plaques and total regional area were measured with the OptiQuant software using the irregular drawing tool at high resolution, with the % area occupied calculated as the sum of plaque areas divided by total regional area. **(C)** The fractional areal values obtained from four brain cases with stage A sorfra plaque pathology, reflecting the occurrence of the lesions primarily in the temporal neocortex and basal gyri of the frontal and occipital lobes. **(D)** The fractional areal values from five brain cases with stage B sorfra plaque pathology, with the lesions occurred additionally in the subiculum, CA1, superior frontal gyrus, and A18. **(E)** The values from four brain cases with stage C pathology, characterized by an additional occurrence of plaque lesions in the primary visual cortex, that is, A17, besides other areas seen at stage B. Note that the mean values are generally increased with the advance of the pathological stage, and there exists an overall difference in the mean values of measured regions at each of the three stages by one-way analysis of variance test.

Based on cross-region comparison in the same brain and between cases as described above, sorfra plaque formation was recognized to progress in cerebral subregions in a stage-dependent manner. To depict the spatiotemporal progression quantitatively, the fractional areas occupied by sorfra plaques were measured in selected neuroanatomical regions, with the areas of interest (AOIs) including representative basally/inferiorly and superiorly located cerebral regions. Thus, the AOIs were the gyral portions of the OrG(l) and SFG, respectively, in the frontal lobe; the middle portions of hippocampal CA1 and the subiculum, and the gyral portions of the ITG and STG, respectively, in the temporal lobe; and the gyral portion of the OTG(l), the middle segment (sulcal portion) of area 17 (A17), and the gyral portion of area 18 (A18) in the cuneate gyrus, respectively, in the occipital lobe (Figures 13A,B).

In four brains with sorfra plaques localized primarily to the temporal, basal frontal, and basal occipital neocortical regions (Figure 13C), the areas occupied by the plaques, expressed as mean ± SD (same format below), were 0.007 ± 0.006% in SFG, 0.67 ± 0.44% in OrG(l), 0.006 ± 0.005% in CA1, 0.002 ± 0.002% in Sub, 1.120 ± 0.885% in ITG, 0.735 ± 0.155% in STG, 1.103 ± 0.185% in OTG(l), 0.296 ± 0.106% in A18, and 0.019 ± 0.033% in A17, respectively. There was an overall significant difference in the means (*n* = 4, *P* < 0.0001, *F* = 7.74, df = 8,27). Specifically, differences (i.e., *P* < 0.05) existed between the basal and superior cortical areas in the frontal [i.e., OrG(l) vs. SFG] and occipital [i.e., OTG(l) vs. A17] lobe cortices, according to Bonferroni multigroup comparison (*post hoc*) test. Differences also existed for CA1/Sub relative to ITG/STG respectively, but neither between CA1 and Sub, nor between ITG and STG.

In five brains with sorfra plaques occurring in the hippocampal formation and occasionally in area 17 (Figure 13D), the areas occupied by the plaques were 1.442 ± 1.062% in SFG, 4.064 ± 1.294% in OrG(l), 1.366 ± 1.000% in CA1, 4.021 ± 1.182% in Sub, 2.972 ± 1.313% in ITG, 2.819 ± 1.552% in STG, 3.275 ± 0.611% in OTG(l), 2.102 ± 0.405% in A18, and 0.469 ± 0.389% in A17, respectively. There was an overall significant difference of the means (*n* = 4, *P* < 0.0001, *F* = 6.95, df = 8,36). *Post hoc* test indicated a significant difference between the SFG and OrG(l) in the frontal lobe. Between the temporal subregions, only the difference between CA1 and Sub reached statistical significance. The mean of A17 was significantly lower than that of the OrG(l), Sub, ITG, STG, OTG(l), and A18, respectively.

In four brains with sorfra plaques occurring in the primary visual cortex (Figure 13E), the fractional areal values were 3.188 ± 0.719% in SFG, 3.101 ± 1.723% in OrG(l), 2.729 ± 1.165% in CA1, 4.809 ± 0.886% in Sub, 5.248 ± 1.257% in ITG, 4.611 ± 1.290% in STG, 3.686 ± 0.316% in OTG(l), 3.322 ± 1.088% in A18, and 2.003 ± 0.635% in A17, respectively. There was an overall statistically significant difference of the means (*n* = 4, *P* < 0.004, *F* = 3.84, df = 8,27). Intergroup comparisons reported that statistically significant differences existed only for A17 relative to Sub and ITG, respectively.

## Discussion

### Sorfra Plaques Are Primarily a Cerebral Neuropathology

While β-amyloidosis and tauopathy occur prominently in the cerebrum, both lesions can develop in subcortical regions. Thus, Aβ deposition and tauopathy are commonly found in subcortical structures in aged or AD brains exhibiting relatively advanced Thal Aβ and Braak NFT stages of neuropathology (Braak and Braak, 1991; Thal et al., 2002; Serrano-Pozo et al., 2011; Braak and Del Tredici, 2014). Accordingly, pathological examination of various subcortical regions is required for the NIH “ABC” scoring for definitive diagnosis of AD (Montine et al., 2012).

We show here that, in contrast to Aβ and pTau pathologies, sorfra plaques do not develop in subcortical structures even in the cases with end-stage Thal Aβ and Braak NFT lesions. As shown in the frontal cerebral sections at the midthalamic levels, sorfra plaques are essentially absent in the striatum, basal forebrain nucleus, thalamus, and hypothalamus wherein substantial amount of β-amyloidosis and tauopathy is present. On the other hand, sorfra plaques are clearly present in the insular cortex and amygdala in the same sections. As illustrated in the Supplementary Material, no sorfra plaques could be detected in other subcortical structures with Aβ and/or pTau pathologies, including the substantia nigra, superior and inferior colliculi, LC, raphe nuclei, brainstem RF, and cerebellum. Thus, unlike Aβ and pTau, sorfra plaques are essentially a cerebral neuropathology.

In line with the existing literature (Tamaoka et al., 1995; Cataldo et al., 1996; Iwatsubo et al., 1996; Mann et al., 1996; Gearing et al., 1997; Thal et al., 2002; Grothe et al., 2017), we find that Aβ deposition in subcortical structures appears predominantly as diffuse parenchymal plaques, along with vascular and subpial amyloidosis at some locations. Consistent with this overall assessment, dystrophic neuritic clusters are rarely found in subcortical structures in BACE1 immunolabeling. In addition, we see that, in the silver stain preparations, typically neuritic plaques are rarely seen in subcortical structures. Overall, the lack of sorfra plaques in subcortical structures is in agreement with our earlier observation that extracellular sortilin neuropathology occurs preferentially in partnership with senile or neuritic amyloid plaques in the human brain (Hu et al., 2017).

### Sorfra Plaques Develop in the Cerebrum in a Stereotypic Spatiotemporal Pattern

**FIGURE 14:**
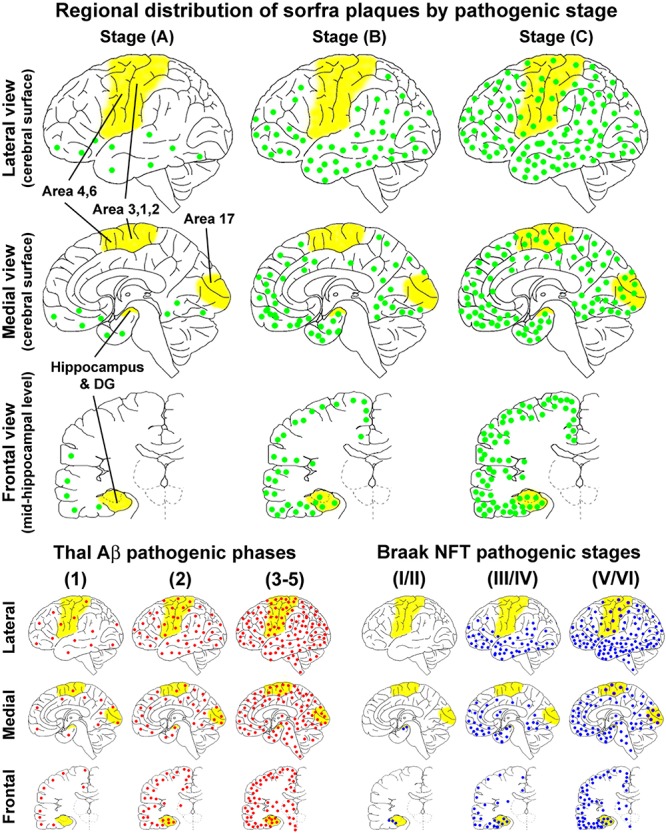
Schematic illustration for a three-staged spatiotemporal progression of sorfra plaques in the cerebrum relative to Thal and Braak staging of β-amyloid and neurofibrillary tangle pathologies. The yellow areas, that is, hippocampal formation, primary motor (areas 4, 6) and somatosensory (areas 3, 1, 2) cortices, and striate cortex (area 17), highlight the neuroanatomical regions wherein the occurrence of neuropathology can define transition between the pathological stages. The upper half of the figure shows the regional distributions of sorfra plaques (represented by green dots) at the three stages, mapped in perspective of the lateral and medial cerebral surfaces, and frontal sectional view at the midhippocampal level, respectively. The low-left group of maps shows the regional distribution of β-amyloidosis (red dots) according to a modified (three-staged) version of the original five-phase Thal Aβ staging (Thal et al., 2002; Serrano-Pozo et al., 2011). The low-right group of maps shows the regional distribution of NFT/tauopathy (blue dots) according to a modified (also three-staged) version of the original Braak six-stage classification (Braak and Braak, 1991; Braak et al., 2006; Montine et al., 2012). The onset of sorfra plaque formation appears to occur along with β-amyloidosis in cerebral neocortex. However, the former is much restricted to the basal associative neocortex, whereas the latter develop over broader neocortical regions in the temporal, frontal, and occipital lobes. From Thal Aβ phase 2 and onward, β-amyloidosis progresses with an increase in the overall amount in the isocortical and allocortical cortex and hippocampal formation and also appears in subcortical structures. On the other hand, sorfra plaques develop in the limbic structures, extend over the associative neocortex, and finally invade the primary neocortical areas, along with the increase of plaque quantity. However, sorfra plaques essentially do not develop in the subcortical structures. In comparison with NFT pathogenesis, sorfra plaques do not develop in the brains with PART. The regional propagation of sorfra plaque formation in the brains with NFT at and above Braak stage III resembles the spatial trajectory of the development of tauopathy. Thus, both lesions extend from inferior to superior gyri in the associative neocortices and expand from the associative to primary neocortical functional areas. However, in temporal order, sorfra plaque pathogenesis precedes tauopathy in the associative and primary neocortical regions. In addition, in contrast to tauopathy, sorfra plaques do not develop in the subcortical regions.

On the basis of the microscopic characterization and quantitative analysis, we propose that the development of sorfra plaques in human cerebrum can be divided into three phases or stages (Figure 14A and Table 1). Thus, stage A involves the formation of the plaques in the temporal, basal frontal, and basal occipital neocortical regions, which could be defined as an early associative isocortical stage. Stage B is featured by the presence of the plaques into the entorhinal allocortex and hippocampal formation, along with an inferior to superior expansion of the lesion in the associative neocortical regions. This stage could be defined as a limbic/allocortical phase. Stage C involves a further progression of the lesion into the primary neocortical functional areas, including the precentral, postcentral, and striatal cortices. This last stage may be termed as a primary isocortical phase of sorfra plaque development. Along with the regional expansion from earlier to later stages, the overall amount of sorfra plaques increases in the cerebral subregions.

### Stages of Sorfra, Aβ, and pTau Pathogenesis Are Partially Overlapped

The clinical course of AD is characterized by progressive memory loss and decline of executive cognitive functions, with neurological dysfunctions occurring at the end disease stage involving failures of sensory, motor, and autonomic functions (McKhann et al., 2011; Long and Holtzman, 2019; Vermunt et al., 2019). Regional vulnerability to neurodegeneration may underlie cerebral malfunction in a domain-specific manner leading to development of cognitive and neurological deficits (Wang et al., 2016; Mrdjen et al., 2019; Veitch et al., 2019). Indeed, cerebral hypometabolism, network disruption, and atrophy occur earlier and more severe in the limbic and associative neocortical regions than in the primary neocortical functional areas (Jagust, 2018; Maass et al., 2019; Mattsson et al., 2019; Pasquini et al., 2019). Studies have also suggested that the extent and regional propagation of NFT/tauopathy, rather than β-amyloidosis, correlate with the advance of AD symptomatology (Price et al., 1991; Arriagada et al., 1992; Hof et al., 1996; Bennett et al., 2006; Aizenstein et al., 2008; Struble et al., 2010; Braak and Del Tredici, 2014; Boluda et al., 2014; Arboleda-Velasquez et al., 2019; Digma et al., 2019; Mrdjen et al., 2019; Panza et al., 2019; Pulina et al., 2019).

The present study indicates that the extent and spatiotemporal progression of sorfra plaque pathology show some similarity to but do not fully parallel those of cerebral β-amyloidosis or tauopathy (Figure 14 and Table 1). Sorfra plaque formation appears at the onset in cerebral neocortex concurrently with β-amyloidosis. However, the former is restricted to the basal associative neocortex, whereas the latter occurs over broader neocortical regions, in the temporal, frontal, and occipital lobes. From Thal Aβ phase 2 and onward, cerebral β-amyloidosis involves largely an increase in the overall amount in the isocortical and allocortical cortex and hippocampal formation, whereas sorfra plaque development proceeds in the cerebral cortex with a distinct spatiotemporal pattern along with the increase of plaque quantity. In comparison with NFT pathogenesis, sorfra plaques do not develop in the brains with PART. The regional propagation of sorfra plaque formation in the brains with NFT at and above Braak stage III resembles the spatial trajectory of the development of tauopathy. Thus, both lesions extend from inferior to superior gyri in the associative neocortices and expand from the associative to primary neocortical functional areas. However, in temporal order, sorfra plaque pathogenesis precedes tauopathy in the associative and primary neocortical regions. As denoted earlier, the spatiotemporal propagation of cerebral NFT pathology appears to concur with the clinical course of AD featured by early cognitive decline and late neurological dysfunction. By the same token, the sequential progression of sorfra plaque pathology would be better related to the advance of AD symptomatology than β-amyloidosis.

### Silver-Stained Neuritic Plaques Are Constituently Heterogeneous

Silver stains (including the Bielschowsky method) are routinely used for postmortem pathological diagnosis of AD, partially because microscopic differentiation of amyloid plaque types based on Aβ immunohistochemistry is not reliable (Alafuzoff et al., 2008). More importantly, only neuritic, but not diffuse, plaques are considered to be of clinical relevance to AD (Serrano-Pozo et al., 2016). According to the NIH guidelines (Hyman et al., 2012; Montine et al., 2012; Jack et al., 2018), silver-stained neuritic plaques are scored based on the protocol established by the Consortium to Establish a Registry for AD (Mirra et al., 1991). The presence of silver-stained diffuse plaques (lacking dystrophic neurites) in a given brain region is scored as “none” or “C0.” The scores of “sparse/C1,” “moderate/C2,” and “frequent/C3” are given when neuritic plaques occur at densities of 1–5, 6–20, and more than 20 profiles per mm^2^, respectively (Mirra et al., 1991). Accordingly, there is less concern whether silver-stained neuritic plaques are actually heterogeneous in context of cellular and molecular constitutions.

Our current multisection and multipathology comparative analyses show that silver-stained neuritic plaques can be constituently variable to a great extent because Aβ, sorfra, and pTau pathologies onset and progress differentially between cerebral structures and cortical subregions. Such a constitutional heterogeneity of neuritic plaques can be clearly recognized in the visual cortex. Thus, before sorfra plaque pathology reaches stage C and NFT pathology reaches stage VI, silver-stained neuritic plaques in area 17 essentially consist of Aβ and BACE1-enriched dystrophic neurites (likely representing sprouting presynaptic terminals) (Yan et al., 2012; Sadleir et al., 2016). Pathologically “full-blossom” neuritic plaques containing Aβ, BACE1, sorfra, and pTau would likely only occur in brains with end-stage AD pathology (i.e., Braak NFT stage VI). As molecular abnormalities become increasingly informative for understanding pathogenic mechanism of diseases and developing mechanism-based biomarkers and therapeutics, the nature of neuritic plaques being constituently heterogeneous would be worth noting in the field of AD research.

## Conclusion

Based on the comparative microscopic examination of brain sections labeled for multiple pathological markers and quantitative analysis, we conclude that (1) sorfra plaques are essentially a cerebral proteopathy; (2) the development of sorfra plaques in cortical and hippocampal subregions proceeds with a stereotypic spatiotemporal order; (3) stages of cerebral sorfra plaque formation partially overlap with that of Aβ and pTau pathologies; (4) silver-stained neuritic plaques are constituently heterogeneous in context of molecular pathology.

## Data Availability Statement

The datasets generated for this study are available on request to the corresponding author.

## Ethics Statement

Use of postmodern human brains was approved by the Ethics Committee for Research and Education at Xiangya School of Medicine, in compliance with the Code of Ethics of the World Medical Association (Declaration of Helsinki).

## Author Contributions

X-XY acquired funding, conceptualized the study, and wrote the manuscript. TT, JJ, Q-LZ, Y-NL, and AP were responsible for study methodology. TT, JJ, LW, and Y-NL curated data. JM reviewed and edited the manuscript. All authors read and approved the final manuscript.

## Conflict of Interest

The authors declare that the research was conducted in the absence of any commercial or financial relationships that could be construed as a potential conflict of interest.
