# Supplementary material for: Extracellular Sortilin Proteopathy Relative to β-Amyloid and Tau in Aged and Alzheimer’s Disease Human Brains
Source: Front Aging Neurosci. 2020 May 12;12:93. doi: 10.3389/fnagi.2020.00093 (PMC7236809; doi:10.3389/fnagi.2020.00093)
Supplement: Supplementary file 1 [file Data_Sheet_1.pdf]

Supplemental Figures

Extracellular Sortilin Proteopathy Relative to β-Amyloid and Tau in Aged and Alzheimer's Disease Human Brains

Tian Tu[1], Juan Jiang[1], Qi-Lei Zhang[1], Lily Wan[1], Ya-Nan Li[1], Aihua Pan[1,2], Jim Manavis[3], Xiao-Xin Yan[1*]

Suppl. Fig. 1. BACE1 IR in the temporal structures of an age brain with primary age-related tauopathy.

Suppl. Fig. 2.1-2.4. Sortilin, Aβ, p-Tau and BACE1 IR in temporal lobe structures from a case with Thal Aβ phase 1 and Braak NFT stage III pathologies.

Suppl. Fig. 3.1-3.3. Sortilin, Aβ and p-Tau IR in frontal lobe structures from a case with Thal Aβ phase 1 and Braak NFT stage III pathologies.

Suppl. Fig. 4.1-4.4. Sortilin, Aβ, BACE1 and p-Tau IR in occipital lobe structures from a case with Thal Aβ phase 1 and Braak NFT stage III pathologies.

Suppl. Fig. 5.1-5.5. Sortilin, Aβ, p-Tau, BACE IR and silver stain in temporal lobe structures from a case with Thal Aβ phase 4 and Braak NFT stage IV pathologies.

Suppl. Fig. 6.1-6.5. Sortilin, Aβ, p-Tau, BACE1 IR and silver stain in frontal lobe structures from a case with Thal Aβ phase 4 and Braak NFT stage IV pathologies.

Suppl. Fig. 7.1-7.5. Sortilin, Aβ, BACE1, p-Tau IR and silver stain in occipital lobe structures from a case with Thal Aβ phase 4 and Braak NFT stage IV pathologies.

Suppl. Fig. 8.1-8.5. Sortilin, Aβ, BACE1, p-Tau IR and silver stain in occipital lobe structures from a case with Thal Aβ phase 5 and Braak NFT stage VI pathologies.

Suppl. Fig. 9.1-9.2. BACE1 IR and silver stain in the insula, amygdala, striatum and diencephalon from a case with Thal Aβ phase 5 and Braak NFT stage V pathologies.

Suppl. Fig. 10.1-10.2. Sortilin, Aβ, BACE1, p-Tau IR and silver stain in the midbrain from a case with Thal Aβ phase 5 and Braak NFT stage VI pathologies.

Suppl. Fig. 11.1-11.2. Sortilin, Aβ, BACE1, p-Tau IR and silver stain in the pons of a case with Thal Aβ phase 5 and Braak NFT stage VI pathologies.

Suppl. Fig. 12.1-12.2. Sortilin, Aβ, BACE1, p-Tau IR and silver stain in the cerebellum of a case with Thal Aβ phase 5 and Braak NFT stage VI pathologies.

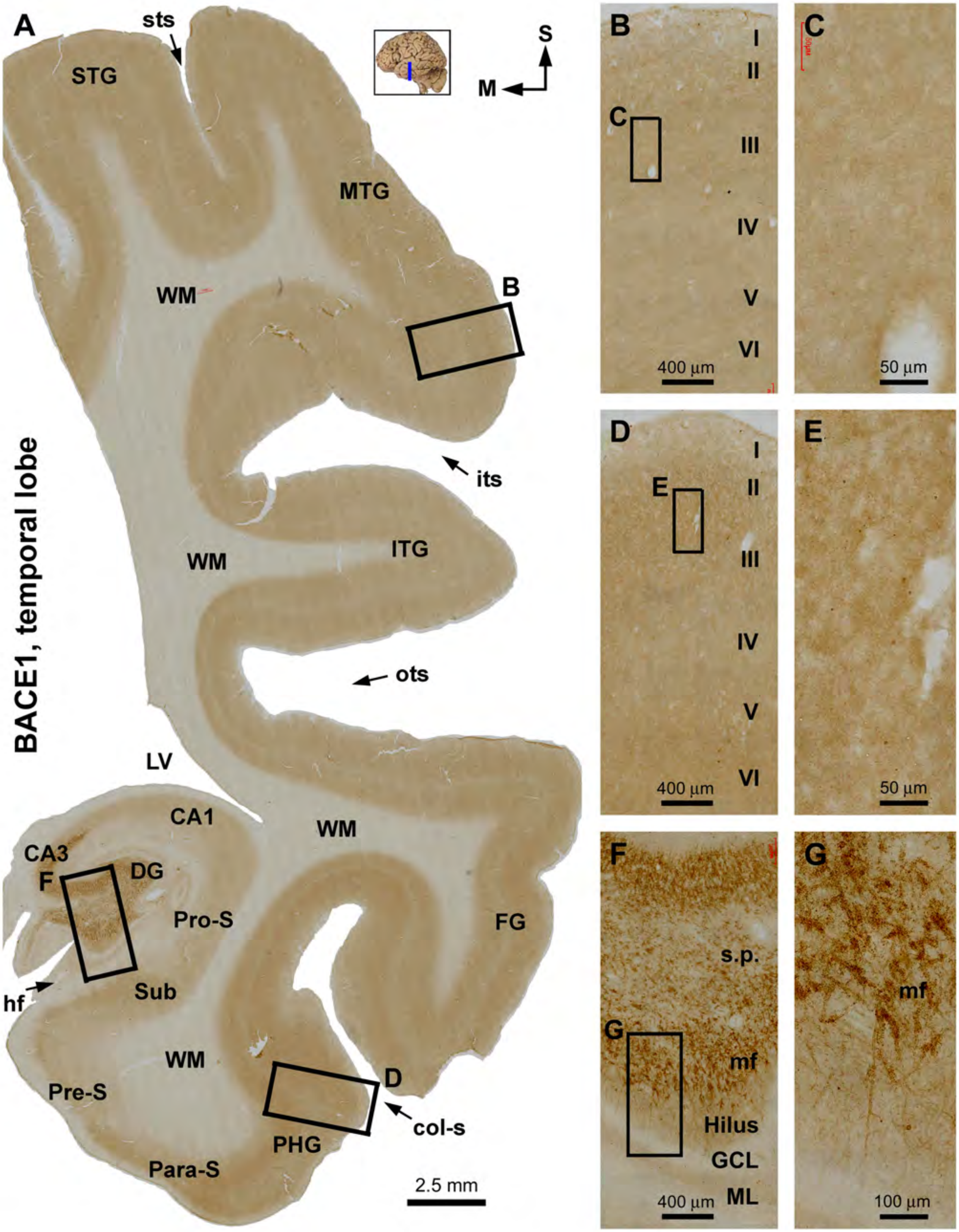

Supplemental Figure 1. β-Secretase 1 **(**BACE1) immunoreactivity (IR) in the temporal lobe structures of an aged brain exhibiting primary age-related tauopathy (PART) at Braak stage III without cerebral amyloidosis (i.e., Thal Aβ phase 0) (referring to Figure 1 in the article). Section orientation (A, top right, with M pointing to medial and S pointing to superior direction in reference to anatomical position), image panel arrangement, neuroanatomical structures (gyri and sulci/fissures), cortical lamination (I-VI: layers I to VI), and scale bars are as labeled. BACE IR occurs as diffuse neuropil-type labeling over the cortical gray matter and hippocampal cellular layers (A-E). The mossy fiber (mf) terminals in the dentate gyrus (DG) and CA3 are distinctly labeled (A, F, G). Abbreviations: STG, MTG and ITG: superior, middle and inferior temporal gyri; FG: fusiform gyrus; PHG: parahippocampal gyrus; sts, its and ots: superior, inferior and occipitotemporal sulci; col-s: collateral sulcus; hf: hippocampal fissure; Sub, Pro-S, Pre-S and Para-S: subicular subregions. GCL: granule cell layer; ML: molecular layer, s.p.: strata pyramidale; LV: lateral ventricle; WM: white matter.

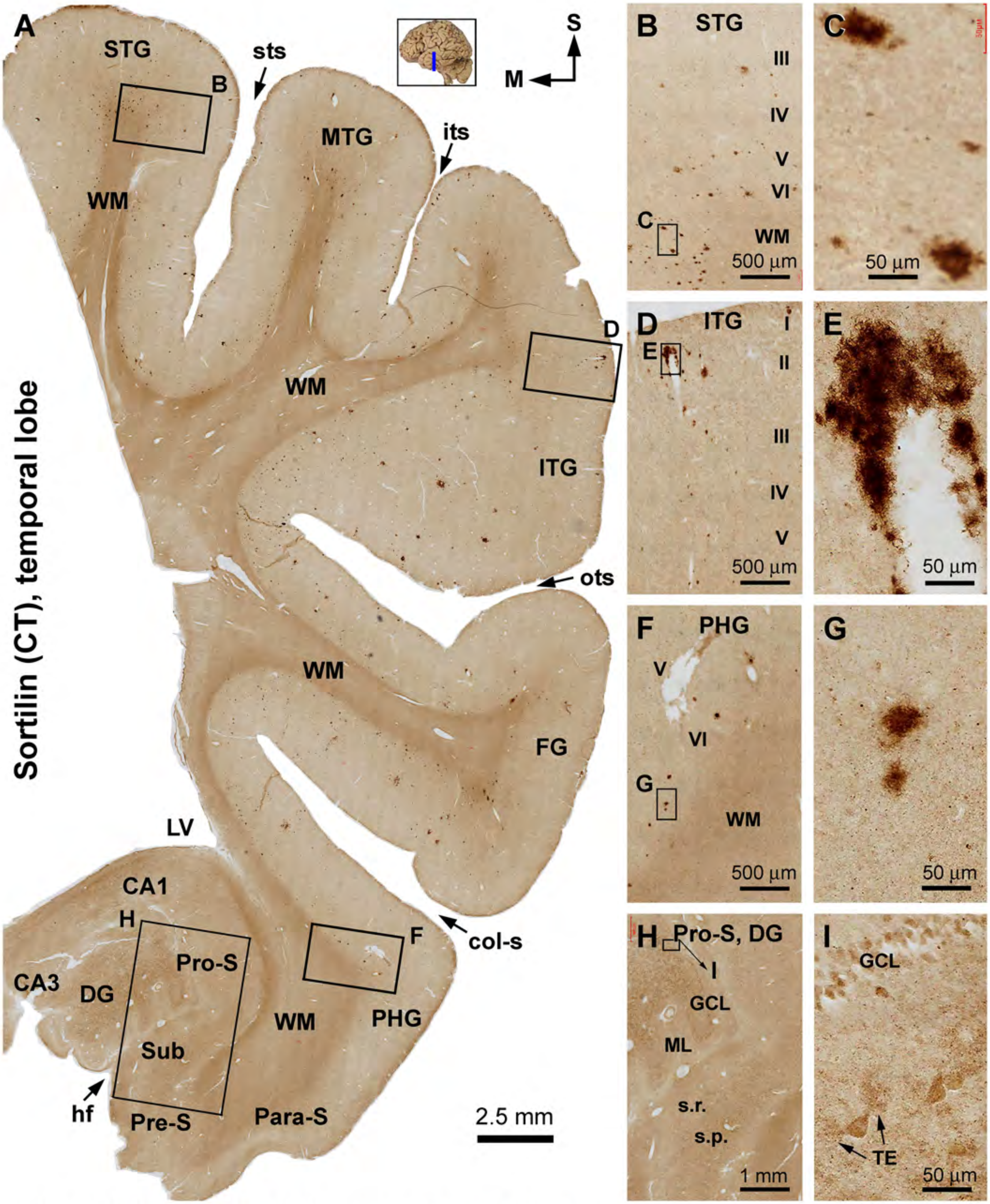

Supplemental Figure 2.1. Sortilin C-terminal antibody labeling in the temporal lobe structures from a case with Thal Aβ phase 1 and Braak neurofibrillary tangle (NFT) stage III pathologies (referring to Figure 2 in the article). Section orientation (M pointing to medial and S pointing to superior in reference to anatomical position), image panel arrangement, neuroanatomical structures, cortical lamination and scale bars are as indicated. Sortilin fragment enriched plaques (defined as "sorfra plaques") occur in an overall low density in the gray matter across the temporal neocortical regions (A-E), sometimes around blood vessels but not in the vascular wall (D, E). The amount of sorfra plaques is reduced as moving from the neocortex into the parahippocampal gyrus (PHG), and becomes absent in the subicular subregions, hippocampal CA sectors and dentate gyrus (DG) (A, F-I). Overall, the plaques are noticeably denser in the deep than superficial layers of the neocortex (A). Light stained neuronal somata are seen in the background, including the mossy cells in the DG with complex dendritic spine formation on these cells, i.e., thorny excrescences (TE), also visualized (H, I). Additional abbreviations are as defined in Figure 1 in the article.

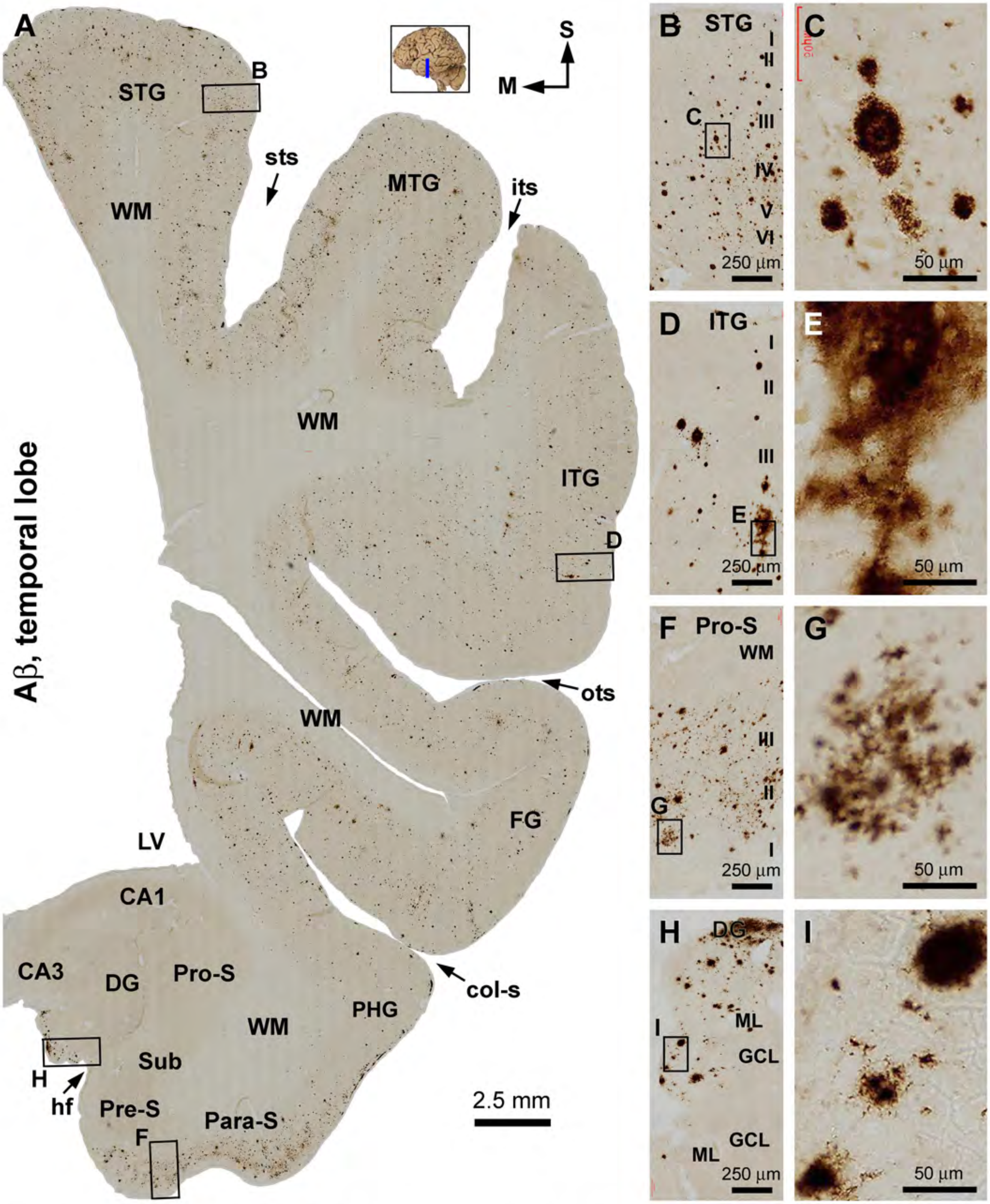

Supplemental Figure 2.2. β-Amyloid (Aβ) immunoreactivity (IR) in the temporal lobe structures from a case with Thal Aβ phase 1 and Braak neurofibrillary tangle (NFT) stage III pathologies (referring to Figure 2 in the article). Section orientation (M: medial; S: superior), image panel arrangement, neuroanatomical structures, cortical lamination and scale bars are as indicated. Aβ IR appears as compact and diffuse plaques as well as meningeal and vascular deposition across the temporal neocortical regions and in the parasubiculum (Para-S) and presubiculum (Pre-S) (A-F). No labeling is present in the subiculum, prosubiculum (Pro-S) and CA1-3 sectors (A). In the dentate gyrus (DG), Aβ deposition likely representing meningeal amyloidosis is present along ventricular surface (A, H, I). Additional abbreviations: STG, MTG and ITG: superior, middle and inferior temporal gyri; FG: fusiform gyrus; sts, its and ots: superior, inferior and occipitotemporal sulci; col-s: collateral sulcus; hf: hippocampal fissure; LV: lateral ventricle; WM: white matter; s.o.: strata oriens; s.p.: strata pyramidale; s.r.: strata radiatum; s.l.m.: stratum lacunosum-moleculare.

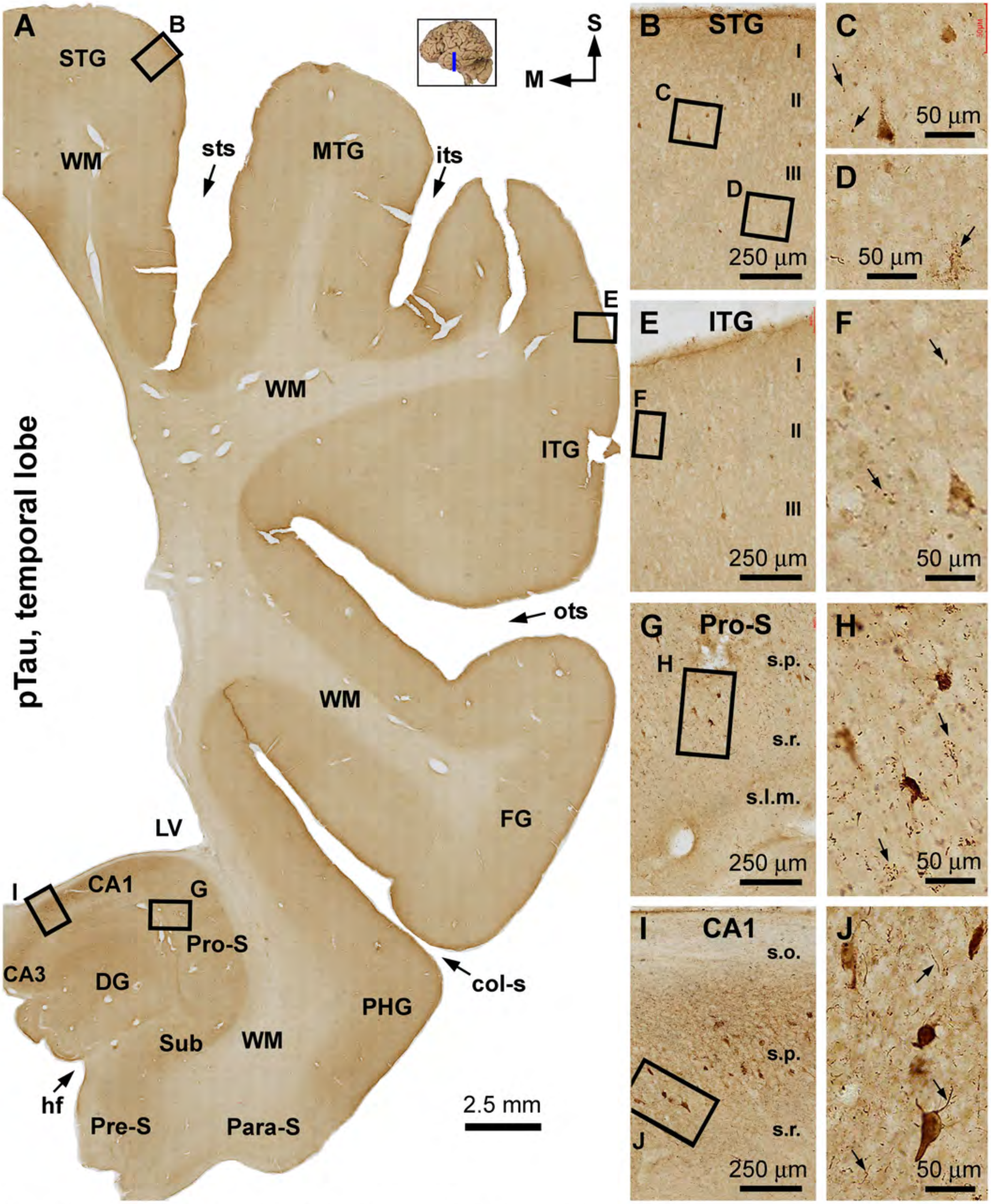

Supplemental Figure 2.3. Phosphorylated tau (pTau) immunoreactivity (IR) in the temporal lobe structures from a case with Thal Aβ phase 1 and Braak neurofibrillary tangle (NFT) stage III pathologies (referring to Figure 2 in the article). Section orientation (M: medial; S: superior), image panel arrangement, neuroanatomical structures, cortical lamination and scale bars are as indicated. pTau IR appears as neuronal and neuritic profiles (pointed by arrows), which are sparsely seen in the temporal neocortical regions (A-F). The amount of labeling is increased as moving from the parahippocampal gyrus (PHG) to subicular subregions (A, G, H), with labeled neuronal somata and processes occurred across the CA1 sector (A, I, J). Some labeled neurons appear tangled, with IR unevenly distributed in the somata, or in dendritic processes appeared to be truncated (F, H, J). Additional abbreviations: STG, MTG and ITG: superior, middle and inferior temporal gyri; FG: fusiform gyrus; sts, its and ots: superior, inferior and occipitotemporal sulci; col-s: collateral sulcus; hf: hippocampal fissure; LV: lateral ventricle; WM: white matter; s.o.: strata oriens; s.p.: strata pyramidale; s.r.: strata radiatum; s.l.m.: stratum lacunosum-moleculare.

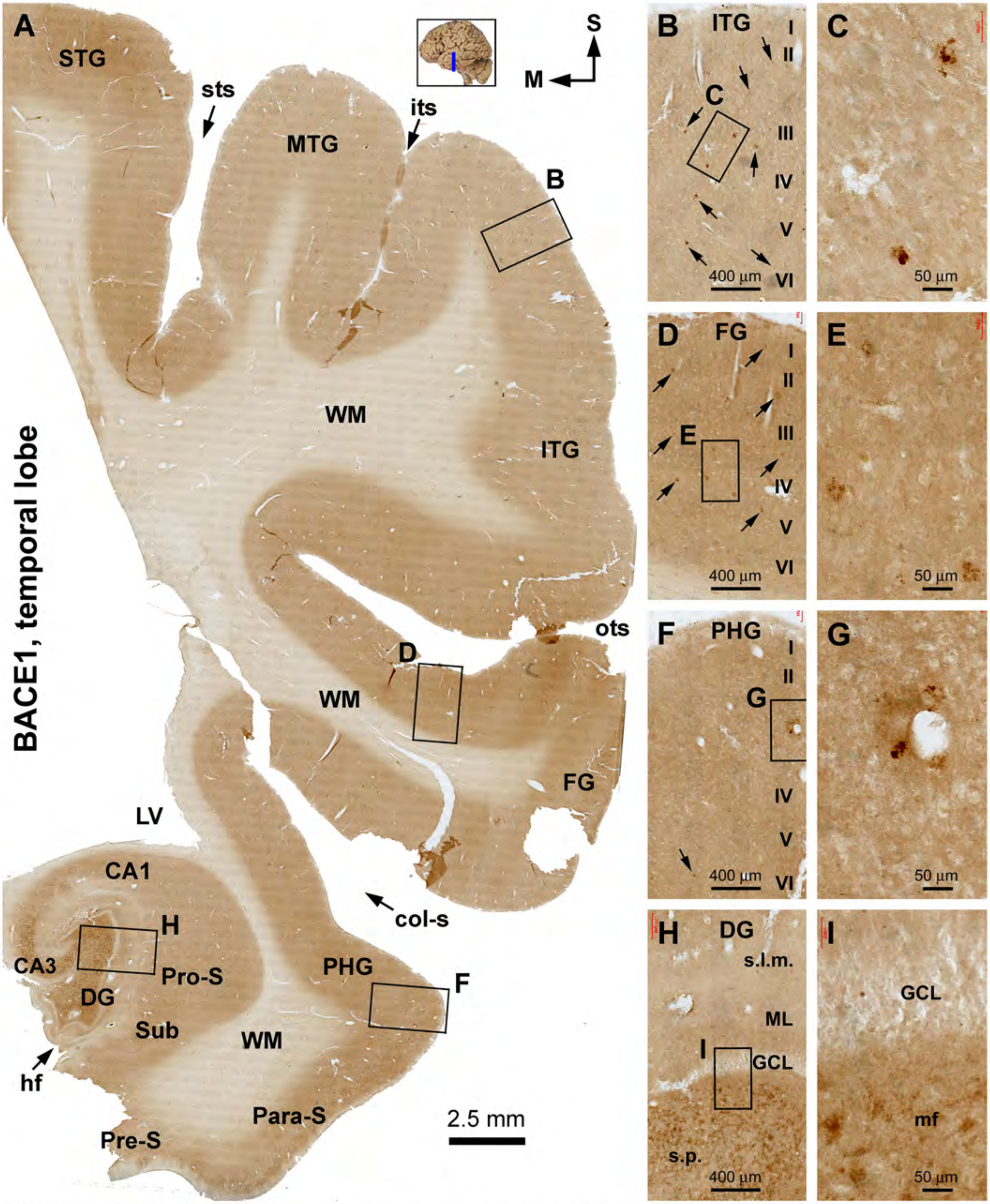

Supplemental Figure 2.4. β-Secretase 1 (BACE1) immunoreactivity (IR) in the temporal lobe structures from a case with Thal Aβ phase 1 and Braak neurofibrillary tangle (NFT) stage III pathologies (in supporting Figure 2 in the article). Section orientation (M: medial; S: superior), image panel arrangement, neuroanatomical structures, cortical lamination and scale bars are as indicated. BACE IR occurs largely as diffuse neuropil-like labeling over the cortical gray matter and the hippocampal cellular layers, except for a distinct punctuate labeling at the terminal field, i.e., CA3 and hilus of the dentate gyrus (DG), of the mossy fibers (A, H, I). However, at high magnifications, there are some heavily stained profiles (as pointed by arrows) across all temporal neocortical areas including the fusiform gyrus (B, D, F, H). These profiles are clusters of dystrophic neurites by close examination, appearing rosette-like in shape and often distributing near microvasculature (C, E, G). Additional abbreviations: STG, MTG and ITG: superior, middle and inferior temporal gyri; FG: fusiform gyrus; sts, its and ots: superior, inferior and occipitotemporal sulci; col-s: collateral sulcus; hf: hippocampal fissure; LV: lateral ventricle; WM: white matter; s.o.: strata oriens; s.p.: strata pyramidale; s.r.: strata radiatum; s.l.m.: stratum lacunosum-moleculare.

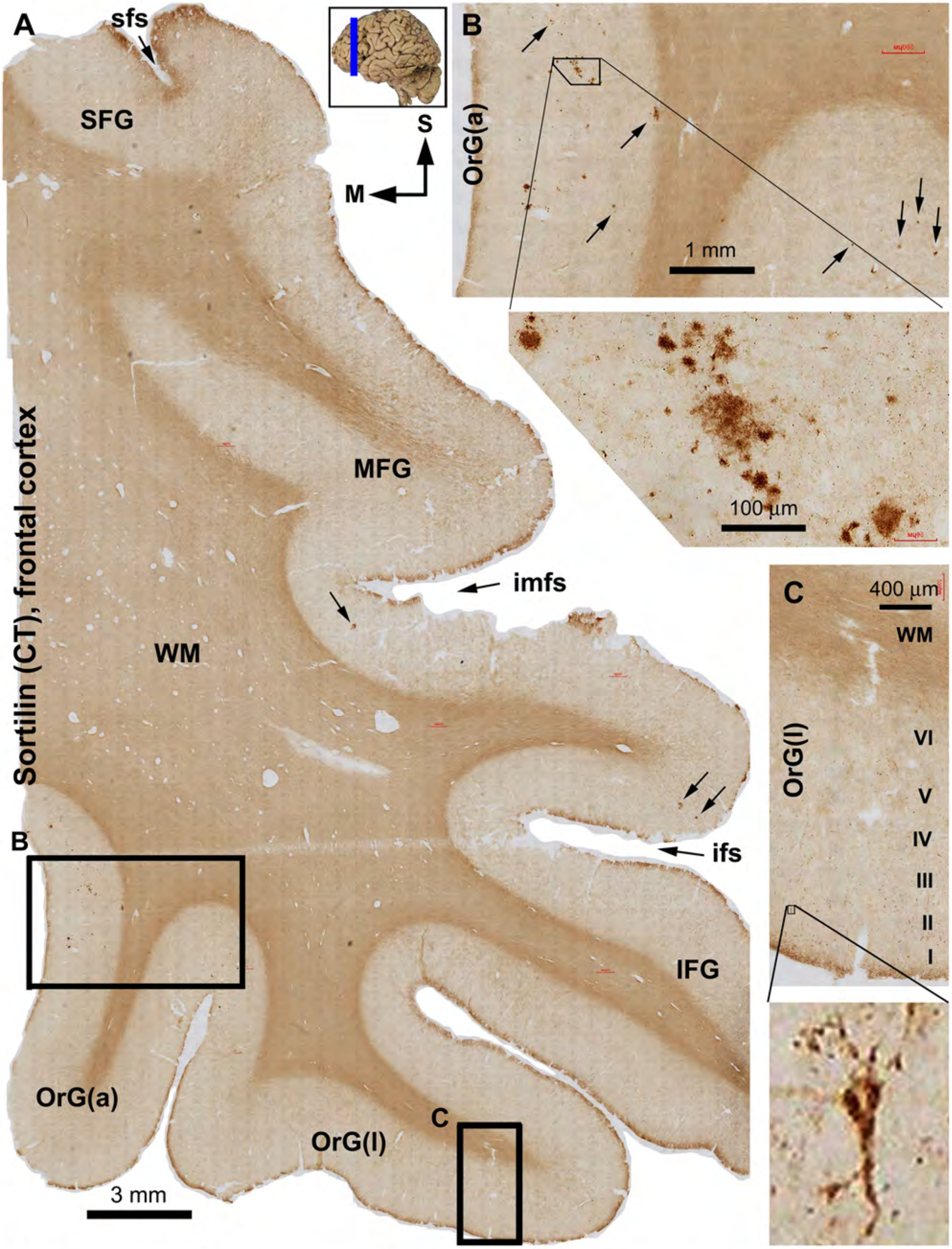

Supplemental Figure 3.1. Sortilin C-terminal (CT) antibody labeling in the frontal lobe from a case with Thal Aβ phase 1 and Braak neurofibrillary tangle (NFT) stage III pathologies (referring to Figure 3 in the article). Section orientation (M: medial; S: superior, in reference to anatomical position), image panel arrangement (enlargements and inserts), neuroanatomical structures and scale bars are as indicated. Labeled extracellular plaques (pointed by arrows) are present mostly and clearly in the cortex of the orbit gyri, especially the anterior orbit gyrus (A, B). In contrast, only one or two plaques can be identified in the middle or inferior frontal gyri (MFG, IFG), and no plaques are found in the superior frontal gyrus (SFG) (A). At high magnification, some pyramidal neuronal somata are labeled in all regions, with immunoreactivity seen in both the somata and dendritic processes (C and enlarged insert). Other abbreviations: sfs: superior frontal sulcus; imfs: intermediate frontal sulcus; ifs: inferior frontal sulcus; WM: white matter.

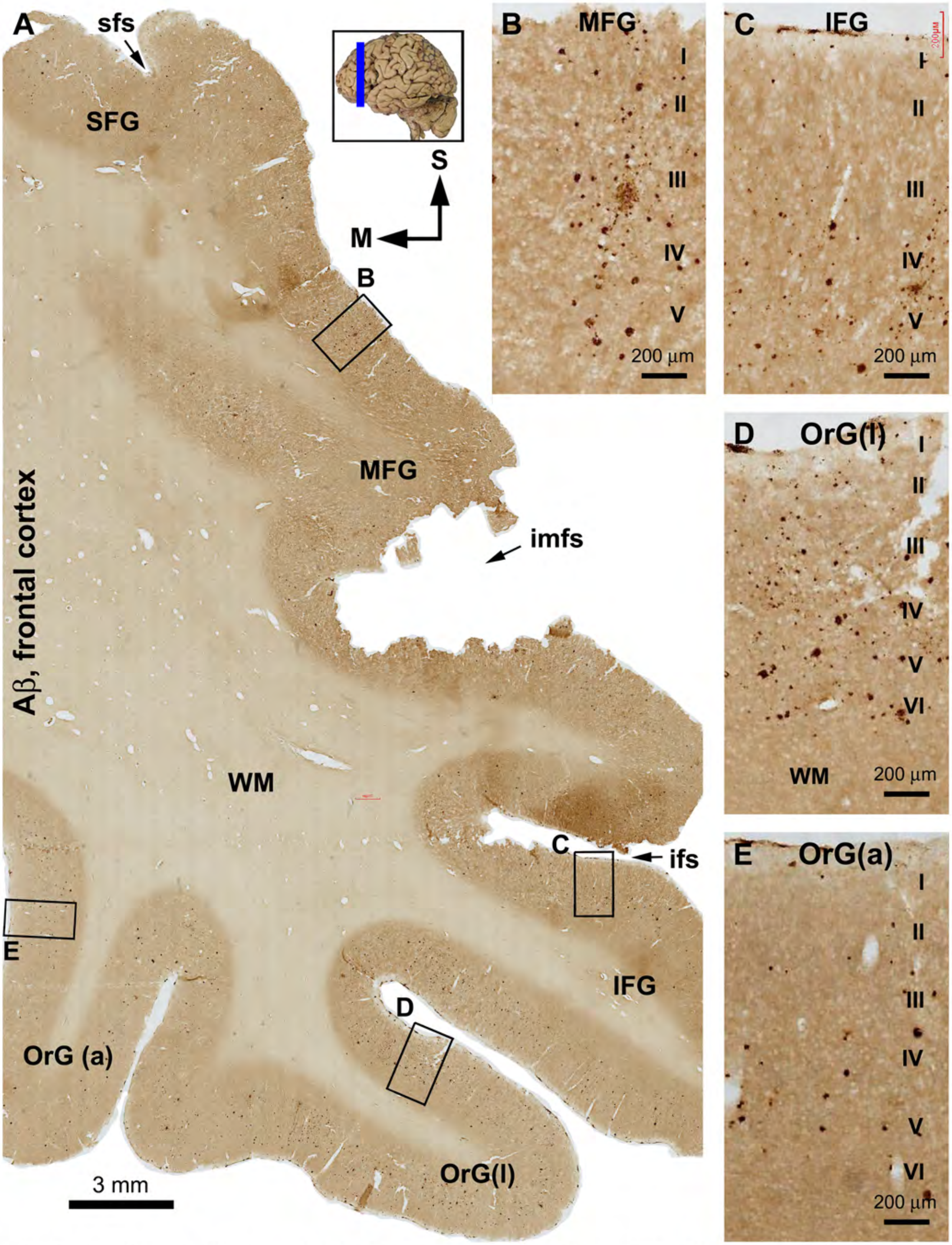

Supplemental Figure 3.2. β-Amyloid (Aβ) immunoreactivity (IR) (stained with the 6E10 antibody) in the frontal lobe structures from a case with Thal Aβ phase 1 and Braak neurofibrillary tangle (NFT) stage III pathologies (referring to Figure 3 in the article). Section orientation (M: medial; S: superior, in reference to anatomical position), image panel arrangement, neuroanatomical structures, cortical lamination and scale bars are as indicated. Aβ deposition appears largely as compact-like plaques, with a relatively low overall density across the basal and lateral frontal cortical areas, including the anterior and lateral orbit gyri [OrG (A) and OrG (L)], the inferior, middle and superior frontal gyri (IFG, MFG and SFG, respectively). The plaques appear slightly denser in the basal (e.g., OrG) than the lateral/superior (i.e., SFG, MFG and SFG) gyri. Other abbreviations are as defined in Supplemental Figure 3.1.

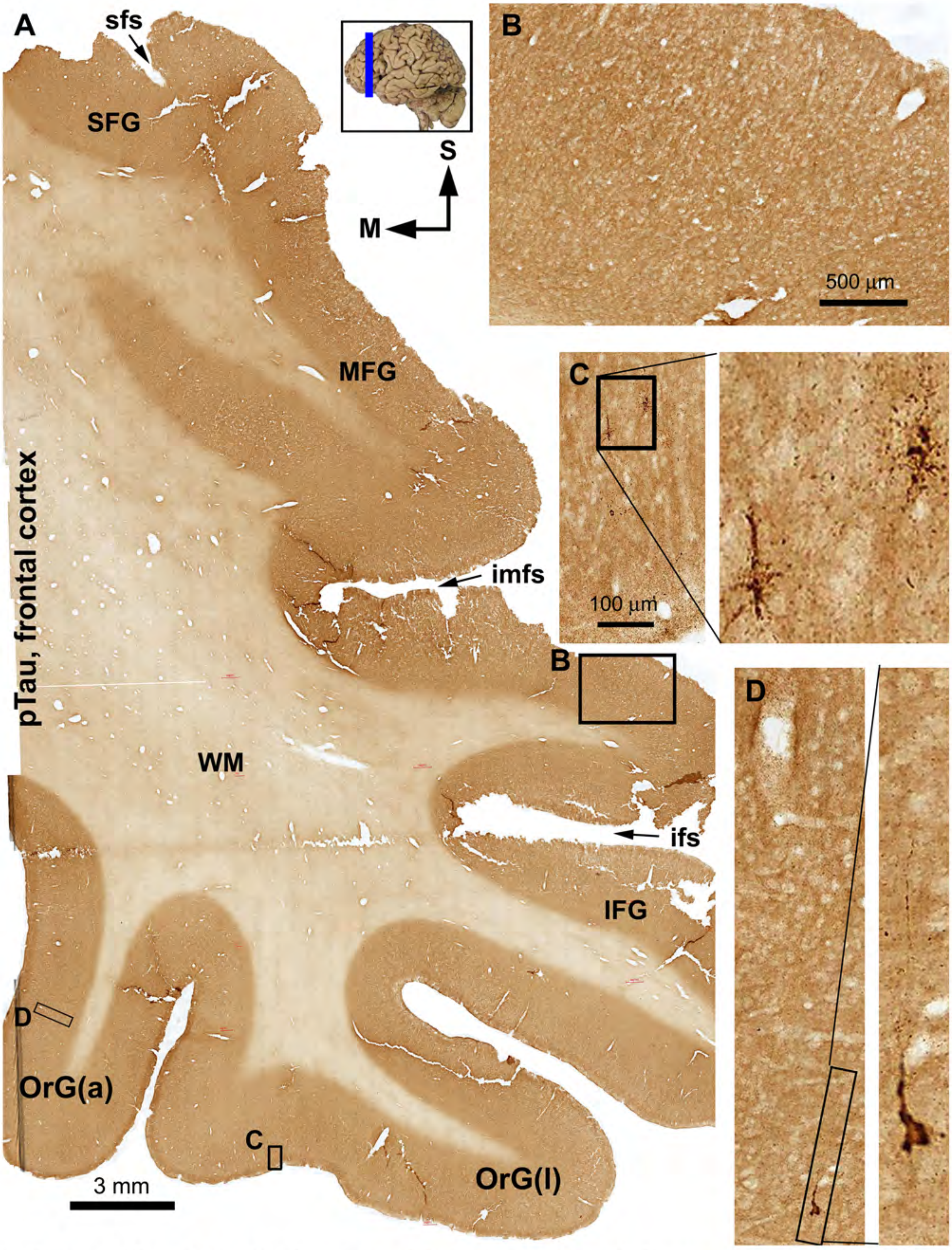

Supplemental Figure 3.3. p-Tau immunoreactivity in the frontal cortical regions from a case with Thal Aβ phase 1 and Braak neurofibrillary tangle (NFT) stage III pathologies (referring to Figure 3 in the article). Section orientation (M: medial; S: superior, in reference to anatomical position), image panel arrangement (enlargements and inserts), neuroanatomical structures (gyri and sulci) and scale bars are as indicated in the low and high magnification images. Overall, p-Tau immunolabeled neurons are rarely observed while scanning over the entire region of the frontal lobe, with only a few labeled neurons are found by close examination. These individually labeled neurons are seen in the basal (A, C, D) but not the lateral (B) cortical regions. Specially, they are present in the anterior and lateral orbit gyri but not in the middle and superior frontal gyri. The somata as well as the dendritic tree of these neurons are well illustrated. However, these neurons are not tangled by close examination. Abbreviations are as defined in Supplemental Figure 3.1.

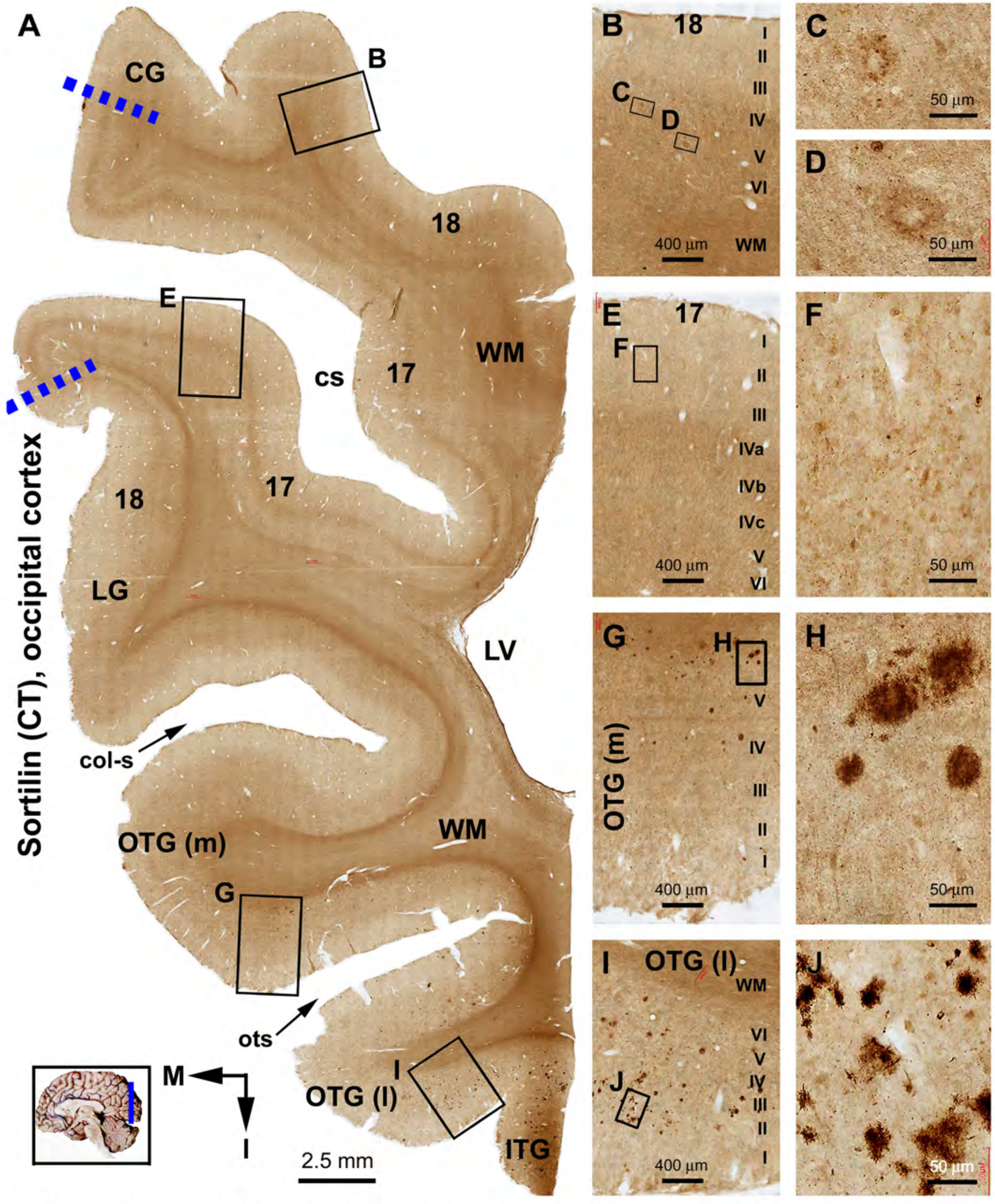

Supplemental Figure 4.1. Occurrence of extracellular sortilin pathology in an occipital lobe section from a case with Thal Aβ phase 1 and Braak neurofibrillary tangle (NFT) stage III pathologies assessed in the brain (referring to Figure 4 in the article). Section orientation (M: medial; I: inferior, in reference to anatomical position), image panel arrangement (enlargements and inserts), neuroanatomical structures, cortical lamination and scale bars are as indicated. The broken blue lines mark the border between areas 17 and 18. Plaque-like lesions are occasionally observed in the upper parts of section (A), with a few lightly stained profiles seen in the area 18 (B, C), while no plaques are seen in area 17 (E, F). However, towards the basal parts of the section (A), the amount of plaques increases in the medial occipitotemporal [OTG (m)] (G, H) and further in the lateral OTG [OTG (l)] (I, J). Lightly stained neuronal somata are also observed at higher magnifications (F, H, J). Additional abbreviations: CG: cuneate gyrus; LG: lingual gyrus; OTG (m): medial occipitotemporal gyrus; OTG (l): lateral occipitotemporal gyrus; ITG: inferior temporal gyrus; col-s: collateral sulcus; ots: occipitotemporal sulcus; LV: lateral ventricle.

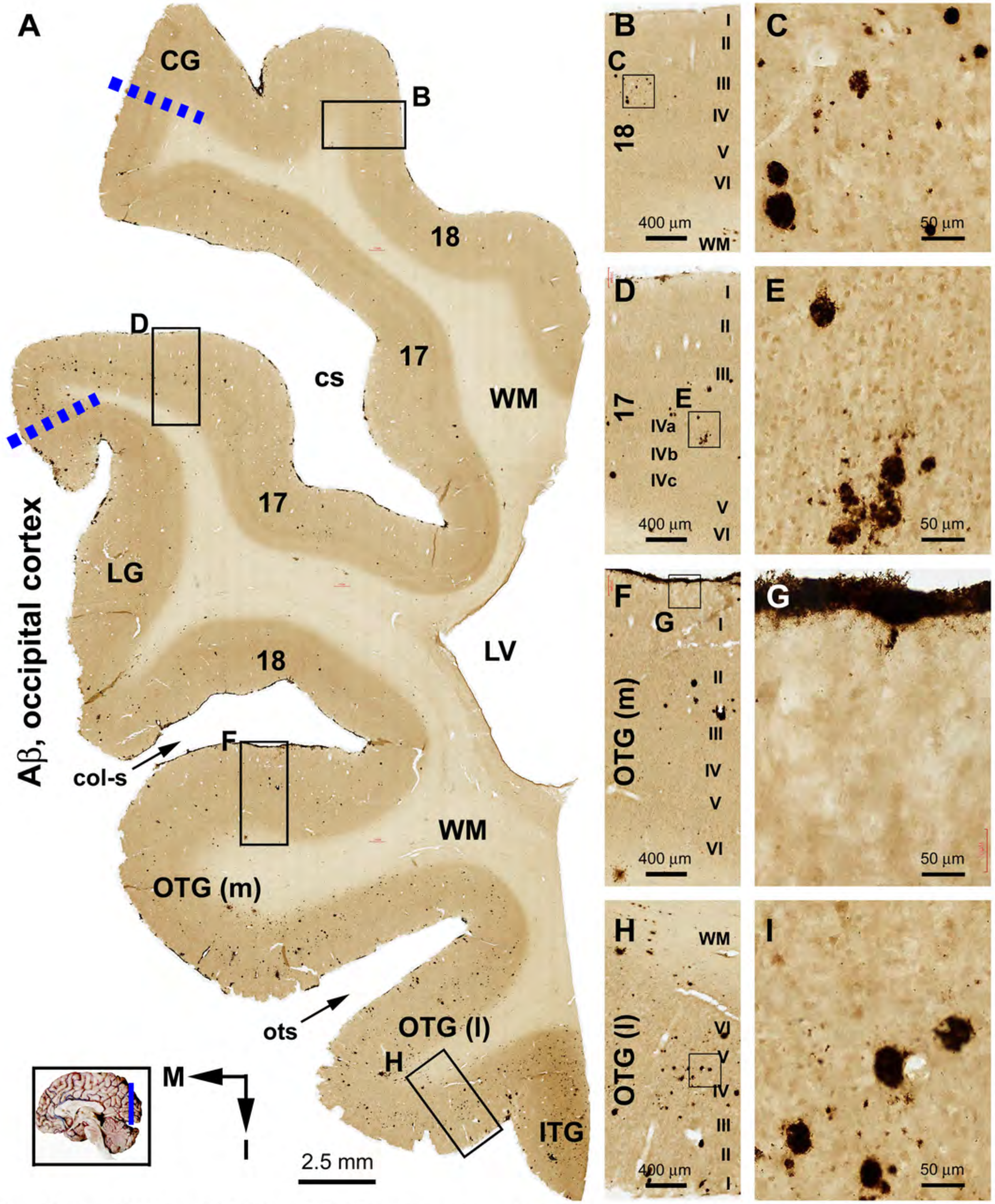

Supplemental Figure 4.2. β-Amyloid (Aβ) immunoreactivity in an occipital lobe section from a case with Thal Aβ phase 1 and Braak neurofibrillary tangle (NFT) stage III pathologies as assessed in the brain (referring to Figure 4 in the article). Section orientation (M: medial; I: inferior, in reference to anatomical position), image panel arrangement (enlargements and inserts), neuroanatomical structures (gyri and sulci), cortical lamination (layers I to VI) and scale bars are as indicated. The broken blue lines mark the border between areas 17 and 18. Aβ deposition appears largely as compact-like plaques located predominantly in the gray matter and occasionally in the white matter (WM) in all gyri (A, B, F). Meningeal amyloidosis is present in most gyri (A, F, G). The density of the plaques is generally low across all the temporal and occipital neocortical areas (A, B, D, F). The amount of plaques appears to be slightly greater in area 17 along the calcarine sulcus (cs) than in area 18 including the superior as well as the inferior parts of latter. Additional abbreviations: CG: cuneate gyrus; LG: lingual gyrus; OTG (m): medial occipitotemporal gyrus; OTG (l): lateral occipitotemporal gyrus; ITG: inferior temporal gyrus; col-s: collateral sulcus; ots: occipitotemporal sulcus; LV: lateral ventricle.

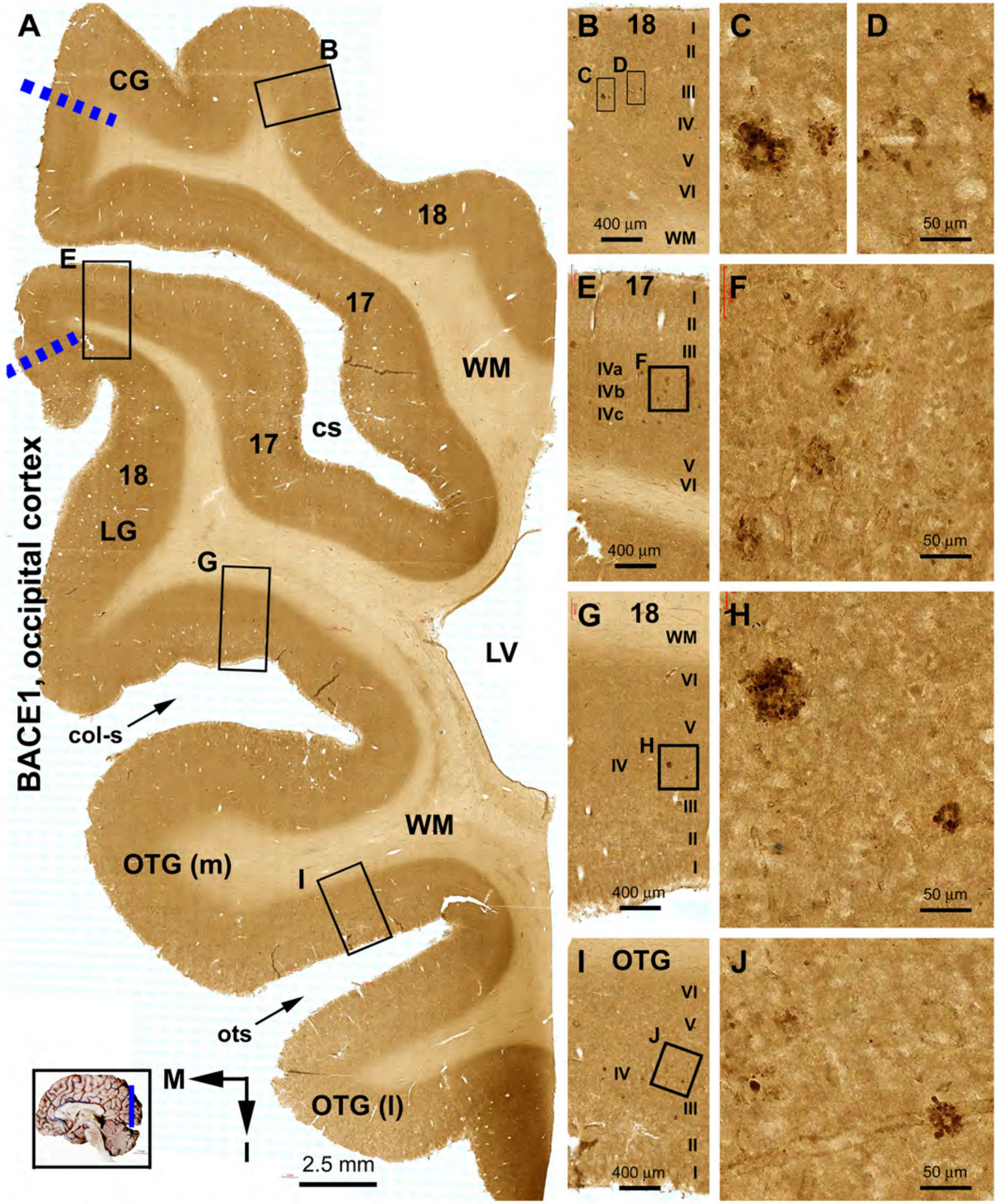

Supplemental Figure 4.3. β-Secretase (BACE1) immunoreactivity in the occipital lobe cortical regions from a case with Thal Aβ phase 1 and Braak neurofibrillary tangle (NFT) stage III pathologies as assessed in the brain (in supporting Figure 4 in the article). Section orientation (M: medial; I: inferior, in reference to anatomical position), image panel arrangement (enlargements and inserts), neuroanatomical structures (gyri and sulci), cortical lamination and scale bars are as indicated. The broken blue lines mark the border between areas 17 and 18 (A). BACE1 labeled dystrophic neurites are observed across the entire section, with a relatively low density (A-J). However, area 17 exhibits a noticeable greater amount of the neuritic clusters in comparison with area 18 while scanning over the regions at higher resolution. The labeled clusters vary in size and in staining intensity. Some of them show a rosette-like appearance with an empty center, while individual and isolated swollen neurites are also observed (C, D, F, H, J). Abbreviations are as defined in Supplemental Figure 4.1.

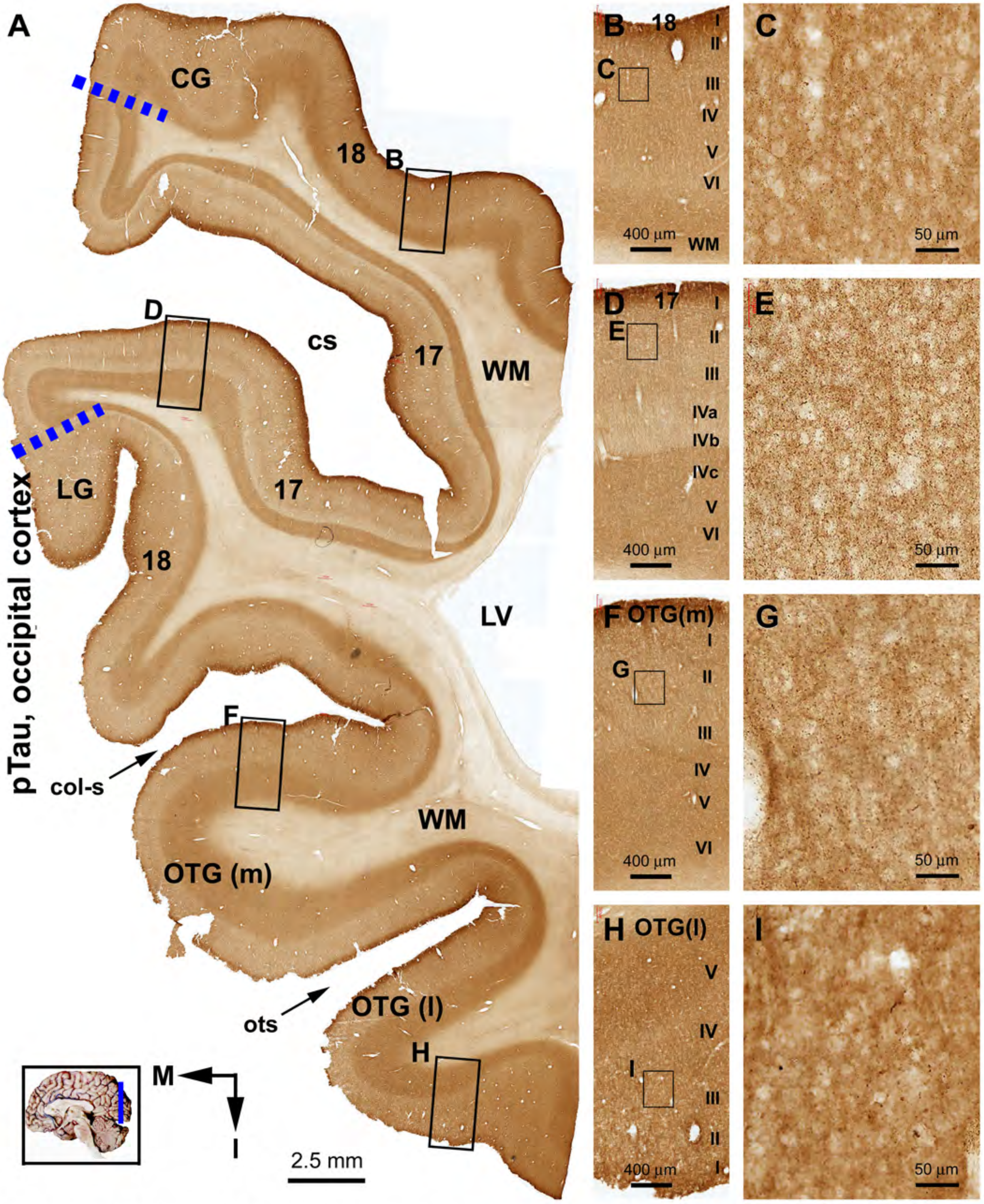

Supplemental Figure 4.4. p-Tau immunoreactivity in the occipital lobe cortical regions from a case with Thal Aβ phase 1 and Braak neurofibrillary tangle (NFT) stage III pathologies as assessed in the brain (in supporting Figure 4 in the article). Section orientation (M: medial; I: inferior, in reference to anatomical position), image panel arrangement (enlargements and inserts), neuroanatomical structures (gyri and sulci), cortical lamination and scale bars are as indicated. The broken blue lines mark the border between areas 17 and 18 (A). Overall, p-Tau IR appears as neuropil like labeling in the cortical gray matter over the entire occipital region, with a band with comparatively heavier reactivity present in layers IVc to VI than other layers in area 17. In contrast to the frontal (Supplemental Fig. 2.3) and temporal (Supplemental Fig. 3.3) lobe regions of the same brain, no labeled somatic or neuritic profiles are detectable in the occipital cortex (B-I). Abbreviations are as defined in Supplemental Figure 4.1.

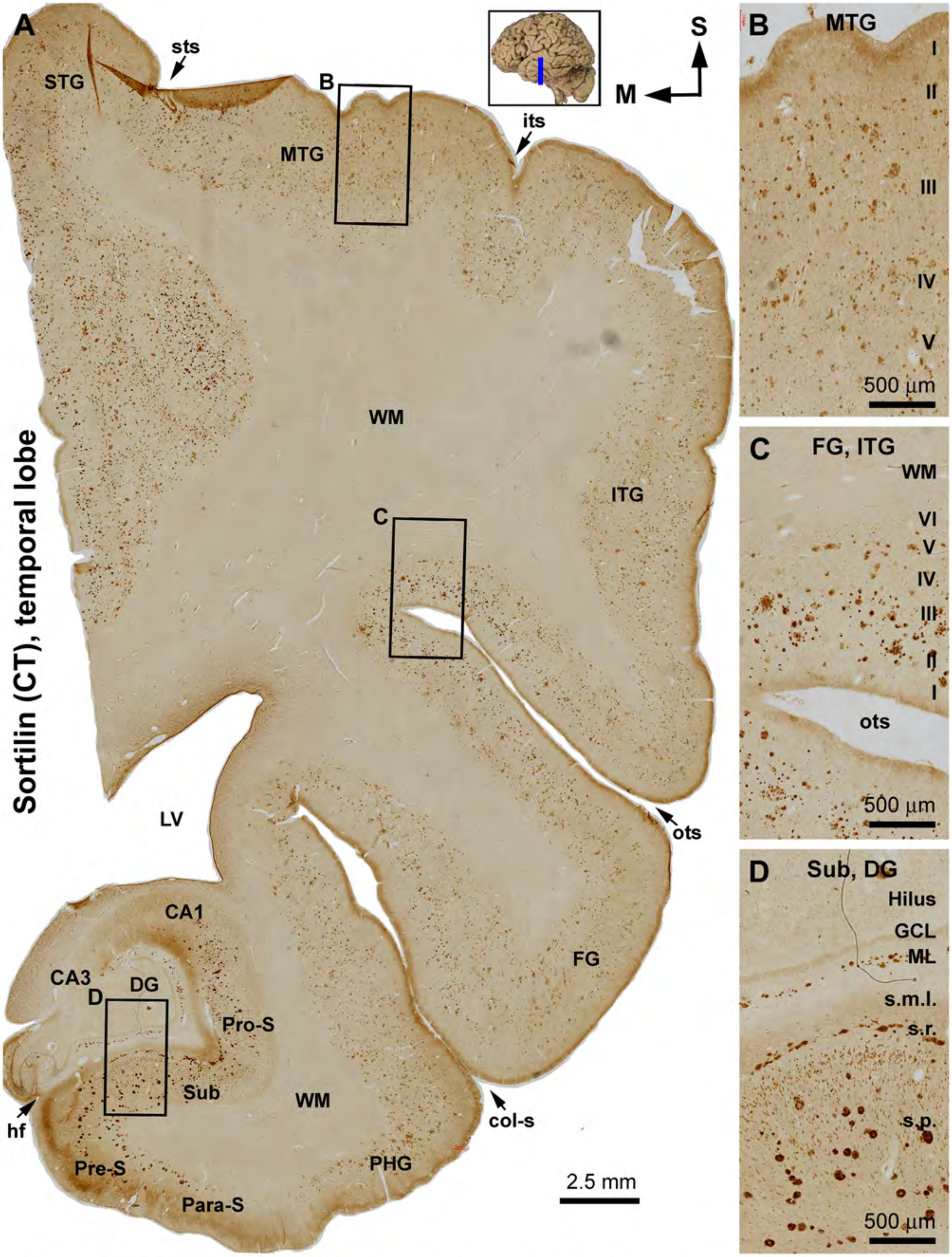

Supplemental Figure 5.1. Distribution of sortilin fragment (shorted as "sorfra") enriched plaques in the temporal lobe structures from a case with Thal Aβ phase 4 and Braak neurofibrillary tangle (NFT) stage IV pathologies (referring to Figure 5 in the article). Section orientation (M: medial; S: superior, in relative to anatomical position), image panel arrangement, neuroanatomical structures, cortical lamination and scale bars are as indicated. A large amount of sorfra plaques occurs in the gray matter across the temporal neocortical areas (A-E) as well as all subregions of the hippocampal formation (A-D). The lesion is not seen in the white matter , vascular wall or the layer II islands of the entorhinal cortex. A row of plaques is present along the molecular layer (ML) of the dentate gyrus (DG) and the strata radiatum (s.r.) of the subiculum (Sub) (A, D), respectively. Abbreviations are as defined in Figures 1, 2 in the article, or in Supplemental Figures 1, 2.1 and 2.2.

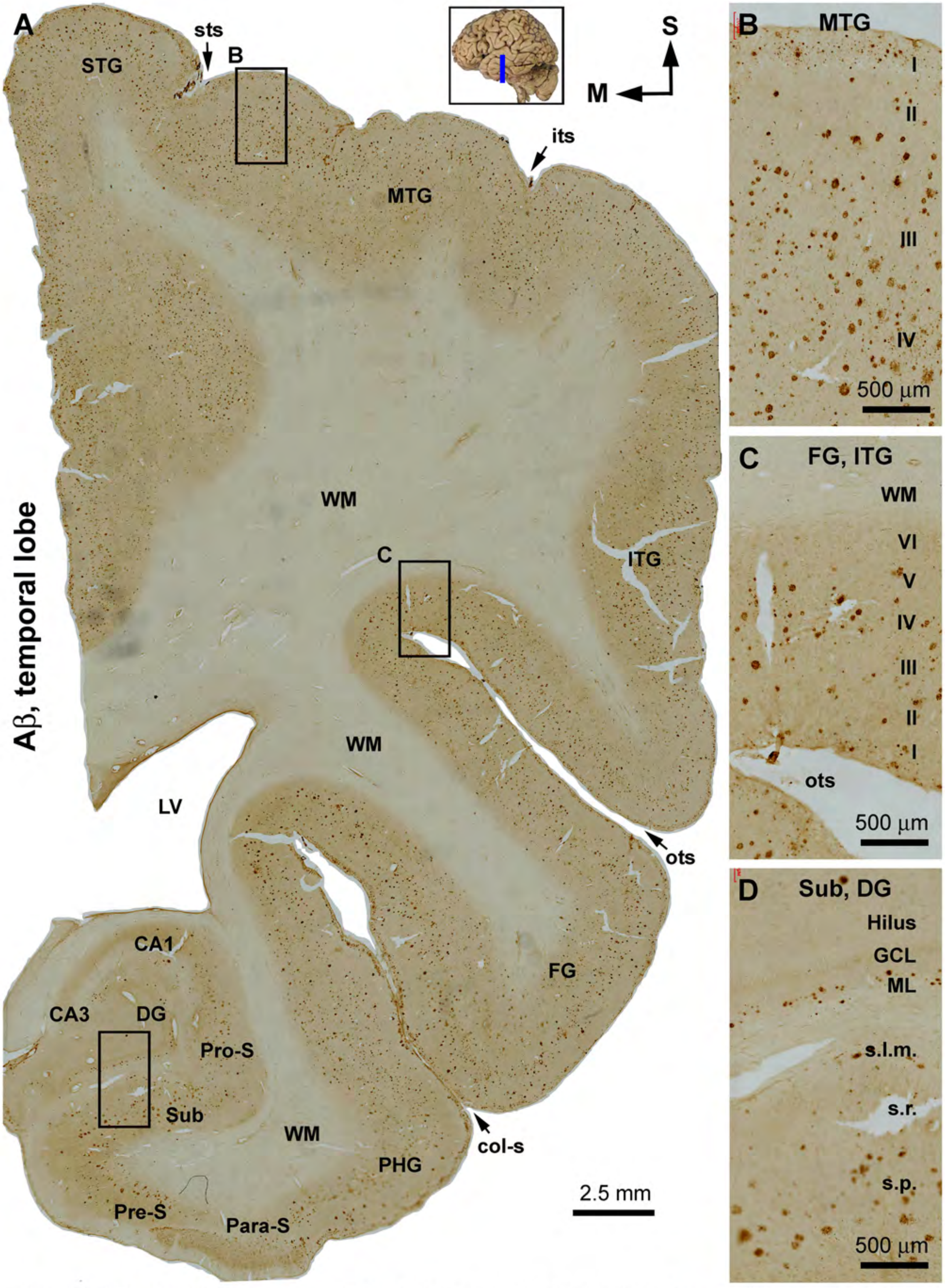

Supplemental Figure 5.2. Aβ immunolabeling in the temporal lobe structures from an AD patient with Thal Aβ phase 4 and Braak NFT stage IV pathologies (referring to Figure 5 in the article). Section orientation (M: medial; S: superior, in reference to anatomical position), image panel arrangement, neuroanatomical structures, cortical lamination and scale bars are as indicated. Large amounts of compact and diffuse plaques occur across the temporal neocortical areas and the hippocampal formation (A-D), including in the DG along the ML (D). Diffuse-type labeling spreads along cortical layer I (B, C) and in layer II cell islands in the parasubiculum (A). A small amount of diffuse deposition also exists in the white matter. Abbreviations are as defined in Figures 1 and 2 in the article as well as Supplemental Figures 1, 2.1. and 2.2.

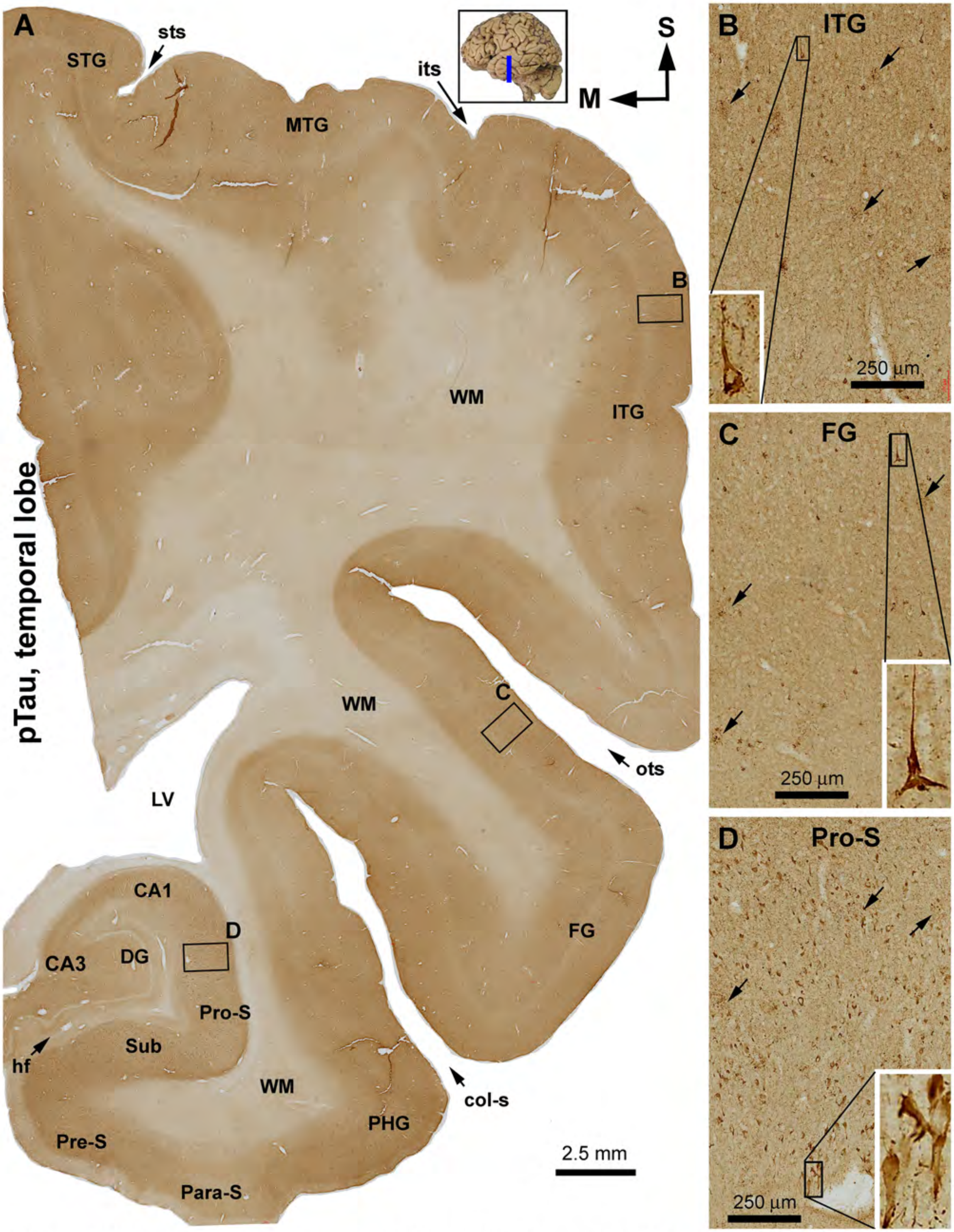

Supplemental Figure 5.3. p-Tau immunoreactivity (IR) in the temporal lobe structures from an AD subject exhibiting Thal Aβ phase 4 and Braak NFT stage IV pathologies as assessed in the whole brain (referring to Figure 5 in the article). Section orientation (M: medial; S: superior, relative to anatomical position), image panel arrangement (enlarged views and inserts), neuroanatomical structures, cortical lamination and scale bars are as indicated. p-Tau IR appearing as neuronal and neuritic profiles (pointed by arrows) occurs extensively in the temporal neocortical regions as well as the hippocampal formation (A-D). The subicular subregions show heavy immunolabeling, with CA1-3 and DG also involved (A, D). A large number of labeled neurons appear to be tangled, as characterized by an uneven or reduced cytoplasmic labeling as well as truncated or rigid-appearing dendritic morphology (inserts). p-Tau positive dystrophic neurites arranged as cluster-like structures (pointed by arrows) are frequently preset in all anatomical parts of the lobe. Abbreviations are as defined in Figures 1 and 2 in the article, and Supplemental Figures 1, 2.1. and 2.2.

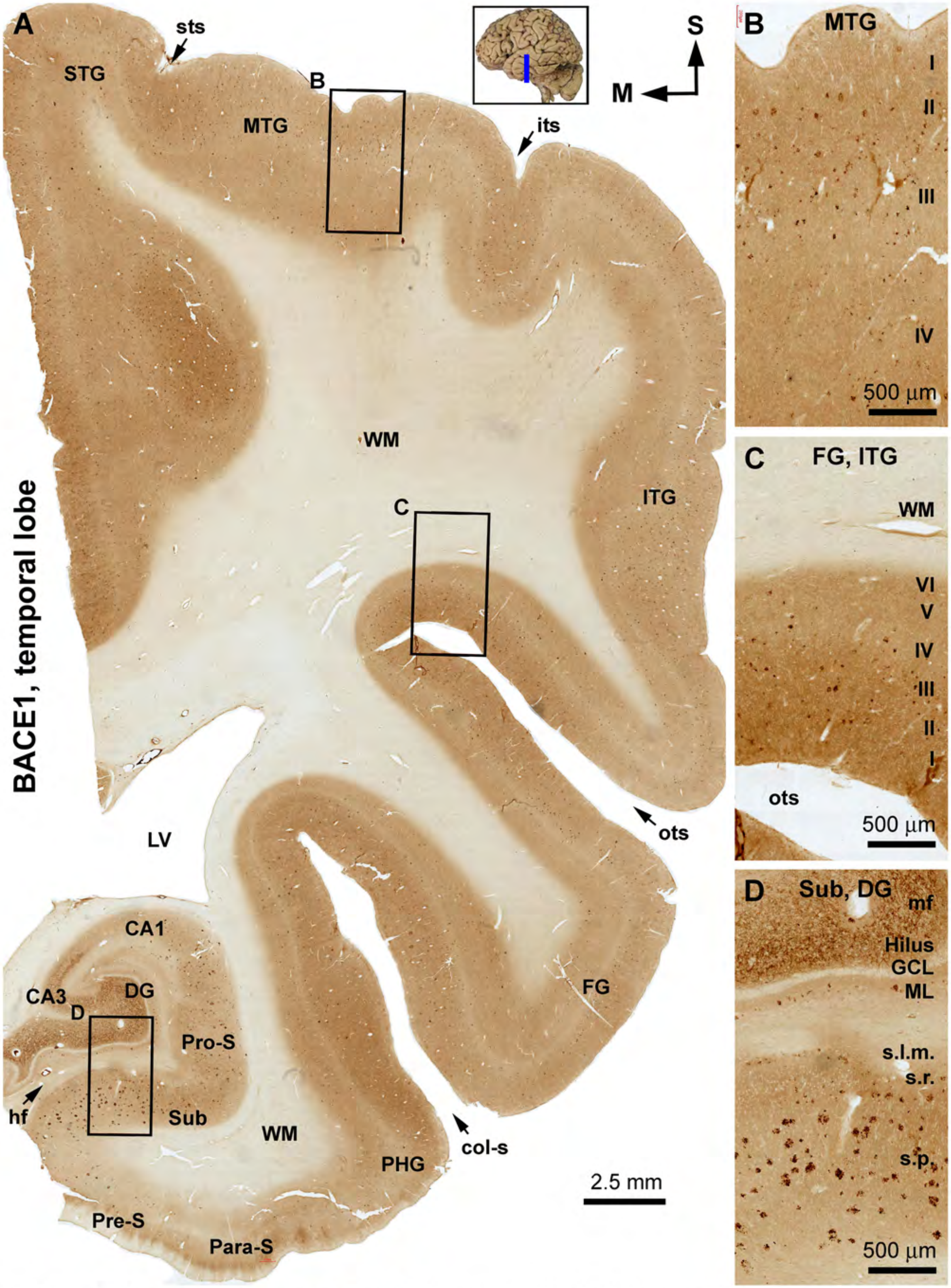

Supplemental Figure 5.4. β-secretase (BACE1) immunolabeling in the temporal lobe structures from a case with Thal Aβ phase 4 and Braak NFT stage IV pathologies (in support of Figure 5 in the article). Section orientation (M: medial; S: superior, relative to anatomical position), image panel arrangement, neuroanatomical structures, cortical lamination and scale bars are as indicated. Clusters of dystrophic axonal terminals occur in fairly large amounts in the grey matter across the temporal neocortical areas and subregions of the hippocampal formation (A-D). Some lightly stained neurites are seen in cortical layer I (B, C). Clusters and isolated dystrophic neuritic are also present in the ML of the DG and s.l.m. and s.r. of CA1, prosubiculum and subiculum (A, D). Abbreviations are as defined in Figures 1 and 2 in the article, Supplemental Figures 1, 2.1. and 2.2.

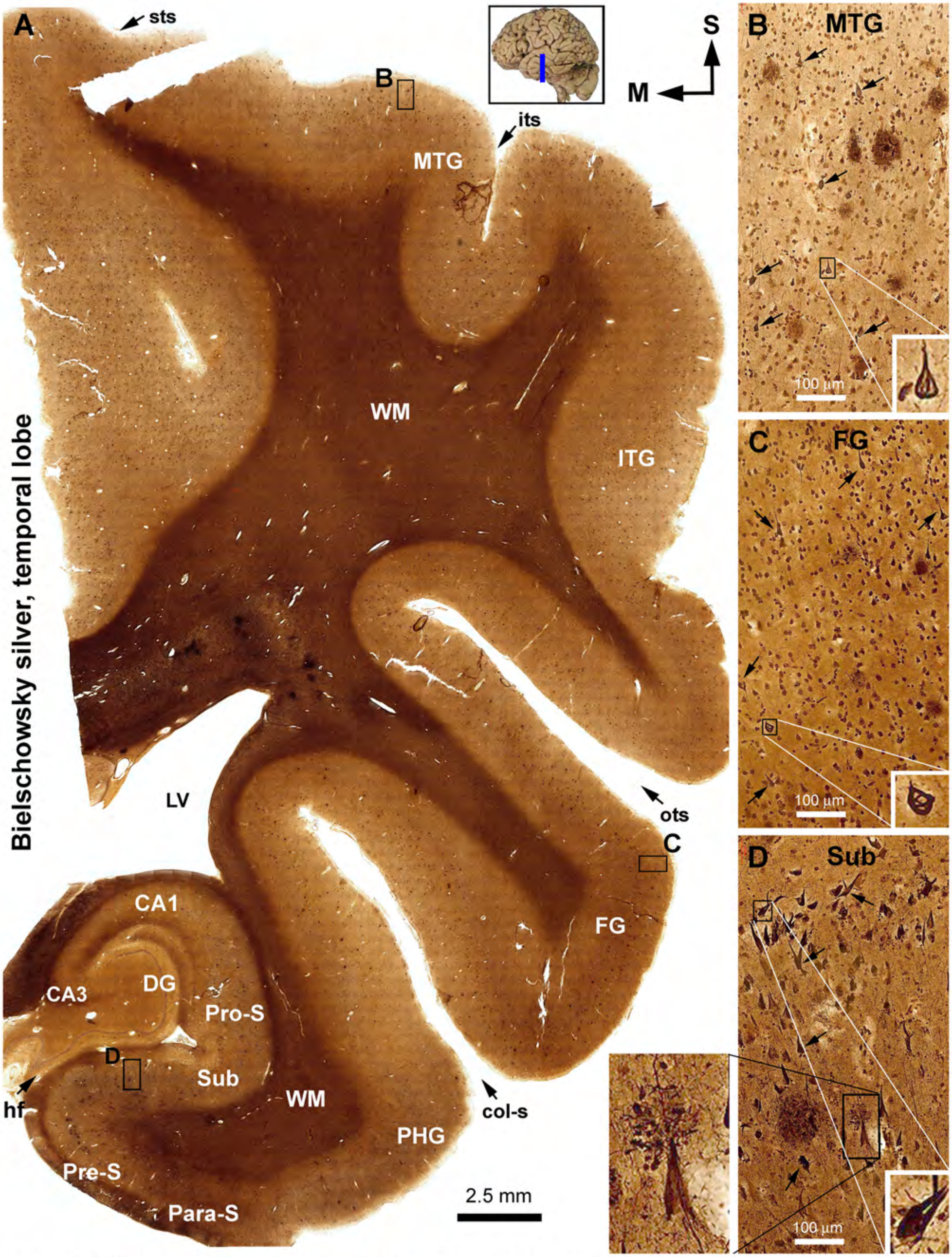

Supplemental Figure 5.5. Bielschowsky silver stain in the temporal lobe structures from an AD patient with Thal Aβ phase 4 and Braak NFT stage IV pathologies (in support of Figure 5 in the article). Section orientation, image panel arrangement, neuroanatomical structures, cortical lamination and scale bars are as indicated. Labeled neuritic plaques and tangled-like neurons (examples are pointed by arrows and also shown as inserts in B-D) are present in fairly large amount in the gray matter across the temporal neocortical areas and subregions of the hippocampal formation (A-D). Some neuritic plaques have a heavily stained dense core (B). Tangled neurons show uneven or reduced silver stain or thread-like elements in the somata, as well as dysmorphic somal and dendritic appearance including a "truncated" dendritic tree (enlarged views shown as inserts in B-D). Abbreviations are as defined in Figures 1 and 2 in the article, or Supplemental Figures 1, 2.1. and 2.2.

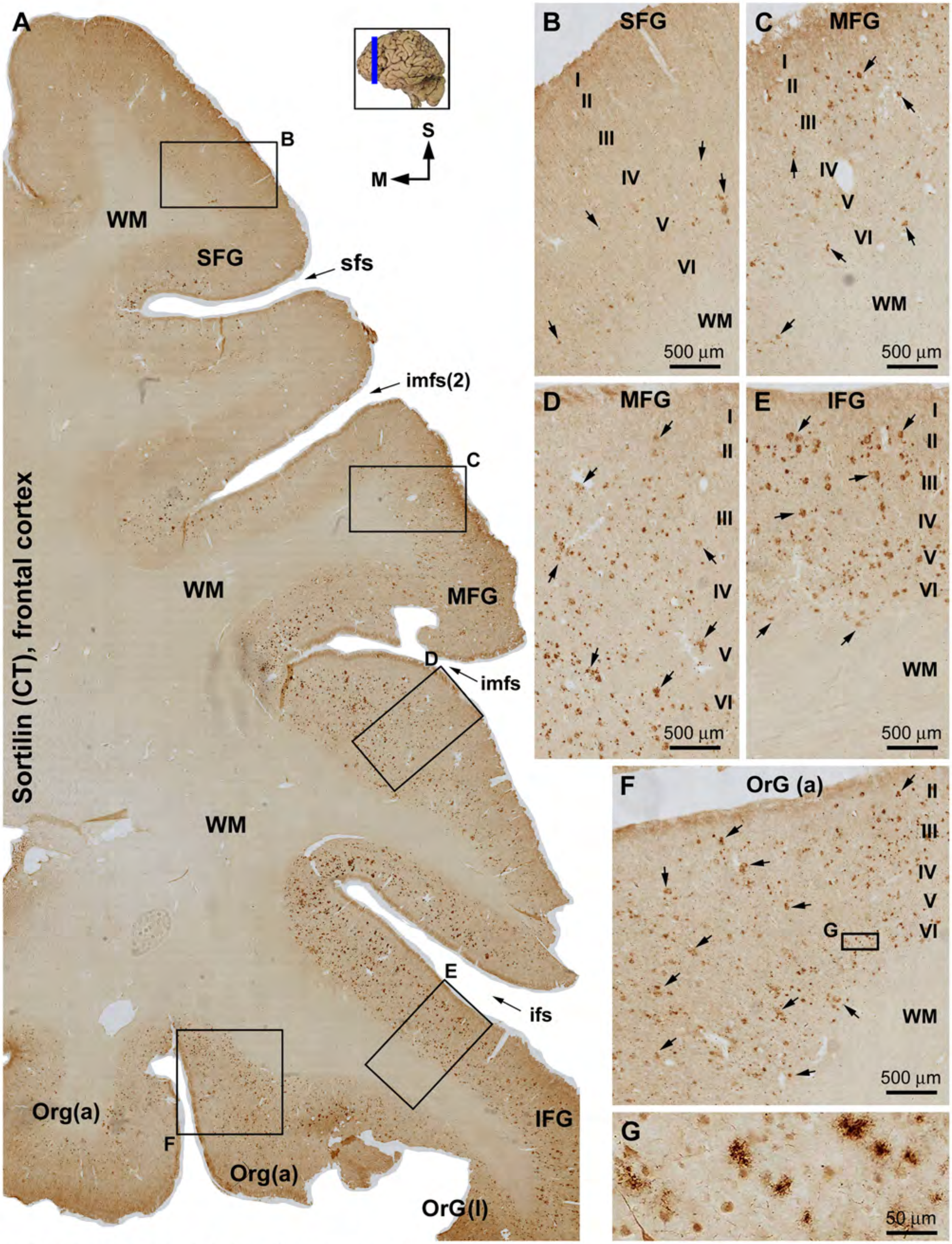

Supplemental Figure 6.1. Distribution of sorfra plaques (examples are pointed by arrows) in a frontal lobe section at the level of the anterior horn from a case of Alzheimer's dementia with Thal Aβ phase 4 and Braak NFT stage IV pathologies (referring to Figure 6 in the article). Section orientation (M: medial; S: superior), image panel arrangement, neuroanatomical structures, cortical lamination and scale bars are as indicated. The overall density of plaques shows a low-to-high gradient from superior to inferior/basal gyri of the frontal lobe (A), as examined while moving from the SFG, MFG, IFG, to the orbit gyri (B-F). In the SFG, plaques appear denser in the deep than superficial cortical layers (B), while in the remaining areas, the plaques are distributed over layers II to VI. No plaques are found in the white matter (A, E, F). At higher magnification, neuronal somata are lightly labeled relative to plaque lesions (G). Abbreviations: AH: anterior horn of the lateral ventricle; SFG, MFG and IFG: superior, middle and inferior frontal gyri; OrG (L) and OrG (A): lateral and anterior orbit gyri; sfs and ifs: superior and inferior frontal sulci; imfs and imfs (2): intermediate frontal sulci.

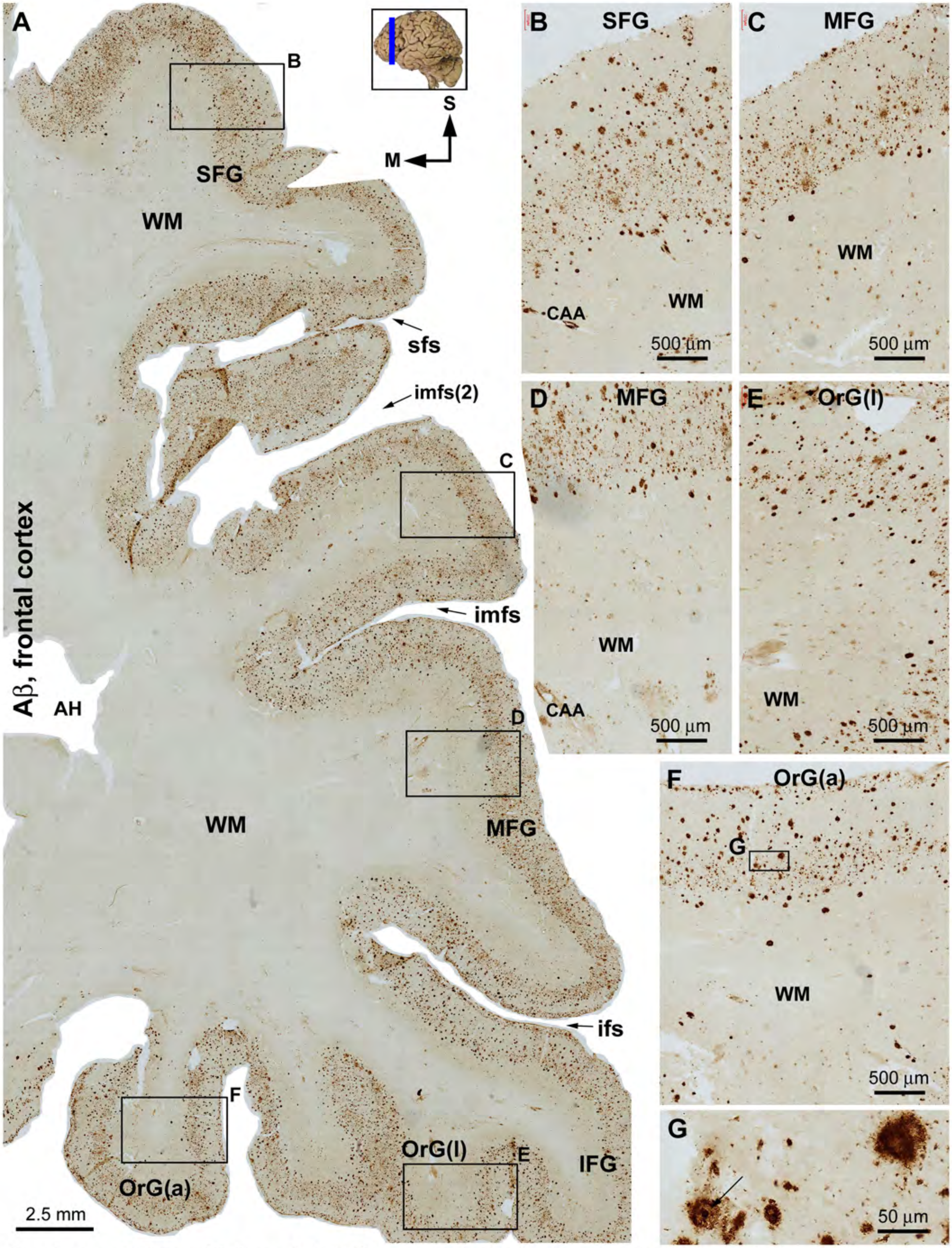

Supplemental Figure 6.2. β-amyloid (Aβ) immunolabeling in a frontal lobe section at the level of the anterior horn of the lateral ventricle from an AD patient with Thal Aβ phase 4 and Braak NFT stage IV pathologies as assessed in the whole brain (referring to Figure 6 in the article). Section orientation (M: medial; S: superior), panel arrangement, neuroanatomical structures, cortical lamination and scale bars are as indicated. Large amounts of compact and diffuse plaques occur with a similar overall density across the lateral and inferior/basal regions of frontal lobe (A-G). Aβ deposition also occurs as meningeal amyloidosis (C, E, F) and cerebral amyloid angiopathy (CAA) (B, D). Lightly stained diffuse lesions are seen in the white matter (C-F). Abbreviations are as defined in Figure 6 and Supplemental Fig. 6.1.

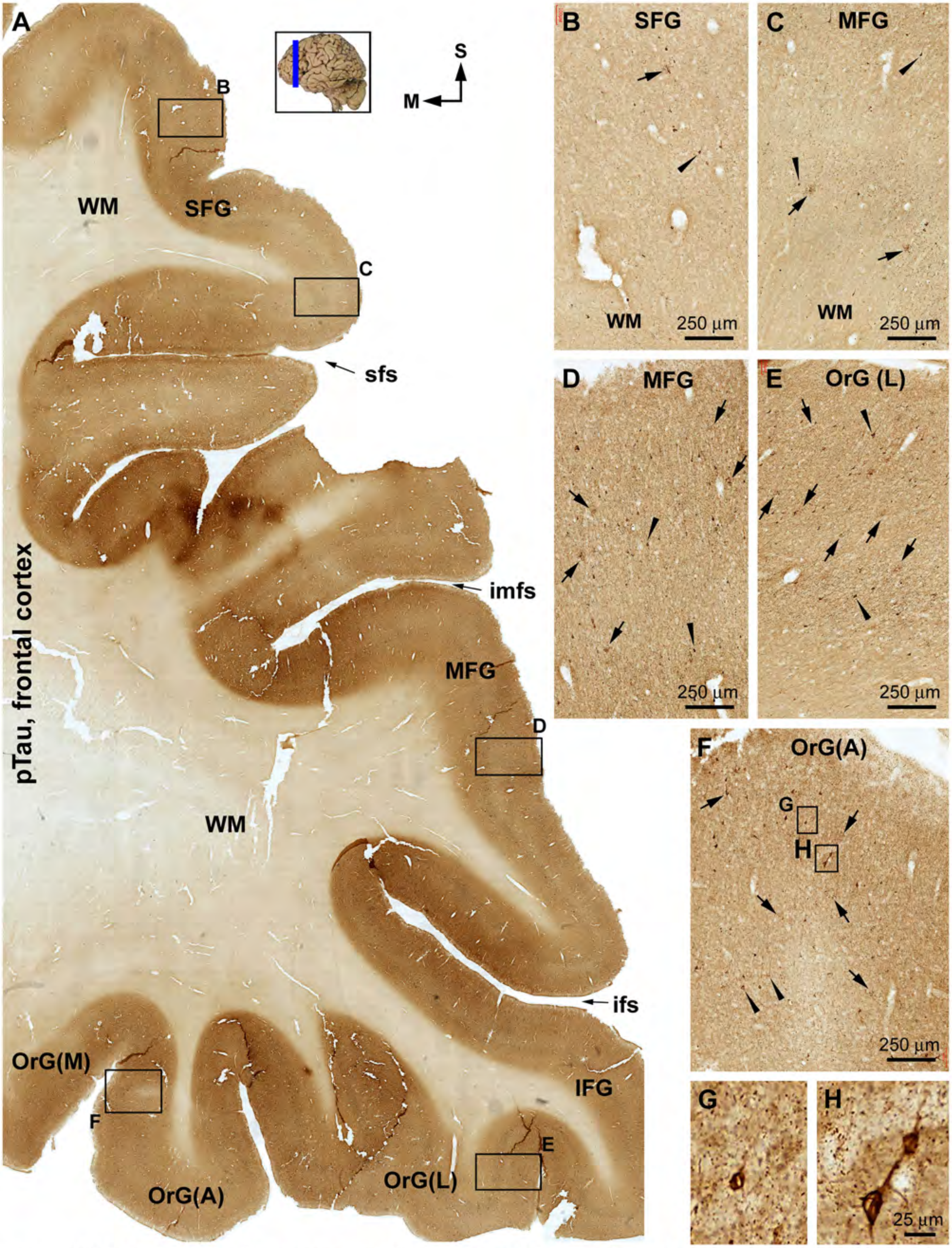

Supplemental Figure 6.3. p-Tau immunolabeling in a frontal lobe section at the level of the anterior horn from a case of Alzheimer's dementia with Thal Aβ phase 4 and Braak NFT stage IV pathologies (referring to Figure 6 in the article). Section orientation (M: medial; S: superior, related to anatomical position), image panel arrangement (enlarged views), neuroanatomical structures, cortical lamination and scale bars are as indicated. The overall density or the amount of p-Tau labeled neurons and neurites appears greater in the basal/inferior than in the lateral/superior gyri of frontal lobe (A-G). Thus, p-Tau labeled neuronal somata and processes are increased as moving superior-inferiorly from the SFG (B), MFG (C, D), IFG (A) to the orbit gyri (F). Labeled neurites occur as clusters likely in association with neuritic plaques (pointed by arrows). Many labeled somata (pointed by arrowheads in B-F) are tangled as examined at high magnifications (as enlarged in G, H). Abbreviations are as defined in Figure 6 in the article and in Supplemental Figure 6.1.

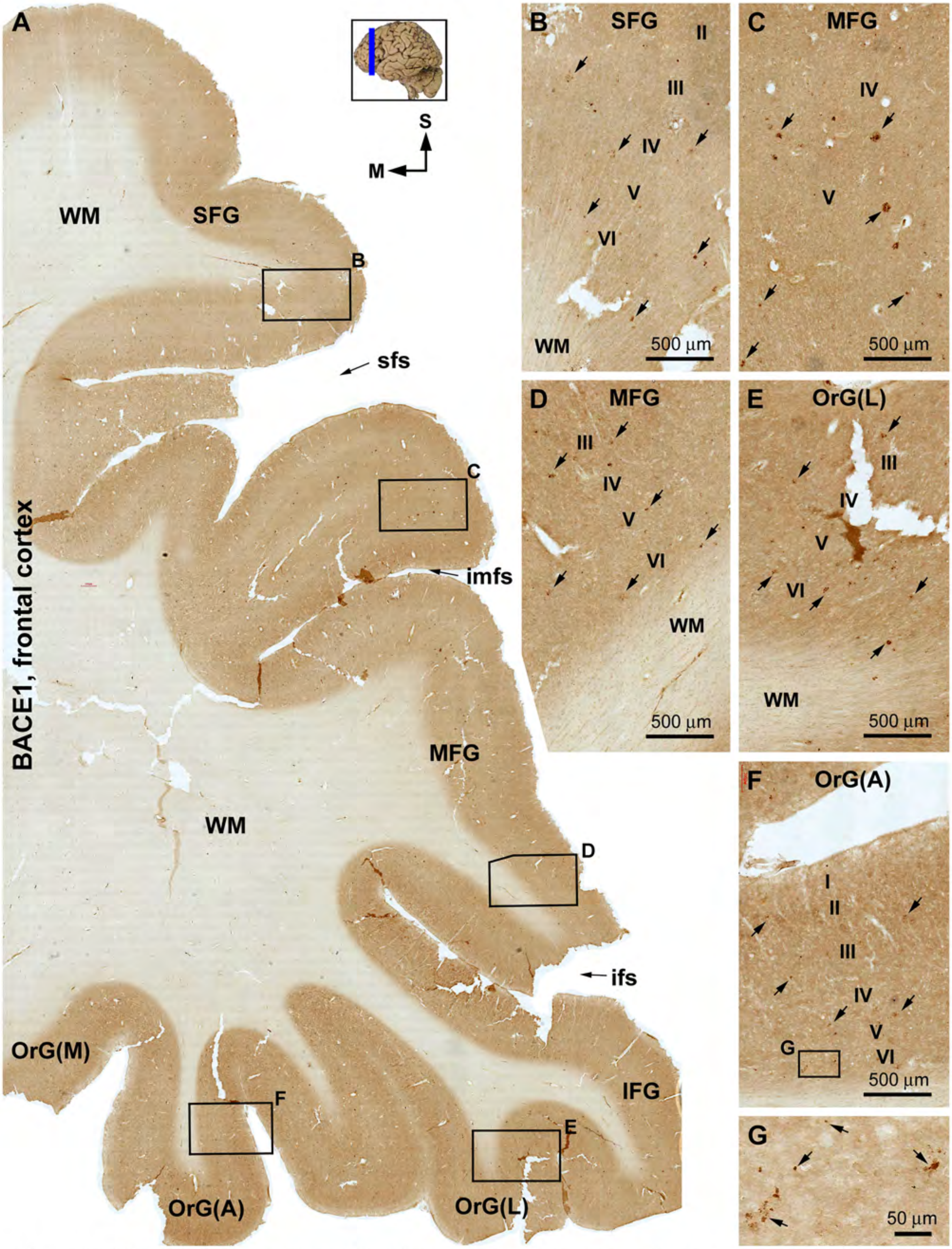

Supplemental Figure 6.4. Distribution of BACE1 immunoreactive dystrophic neurites in a frontal lobe section at the level of the anterior horn from a case of Alzheimer's dementia with Thal Aβ phase 4 and Braak NFT stage IV pathologies (in support of Figure 6 in the article). Section orientation (with M directing to medial and S directing to superior in reference to anatomical position), image panel arrangement, neuroanatomical structures, cortical lamination and scale bars are as indicated. The overall density of the labeled neuritic clusters (as pointed by arrows for example) appear comparable as moving from the SFG, MFG, IFG and orbit gyri (A-F). At higher magnification, the labeled neurites are swollen and sprouting neuronal terminals that arrange in rosette-like clusters, or as isolated individual spherical profiles (G). Abbreviations are as defined in Figure 6 in the article and in Supplemental Figure 6.1.

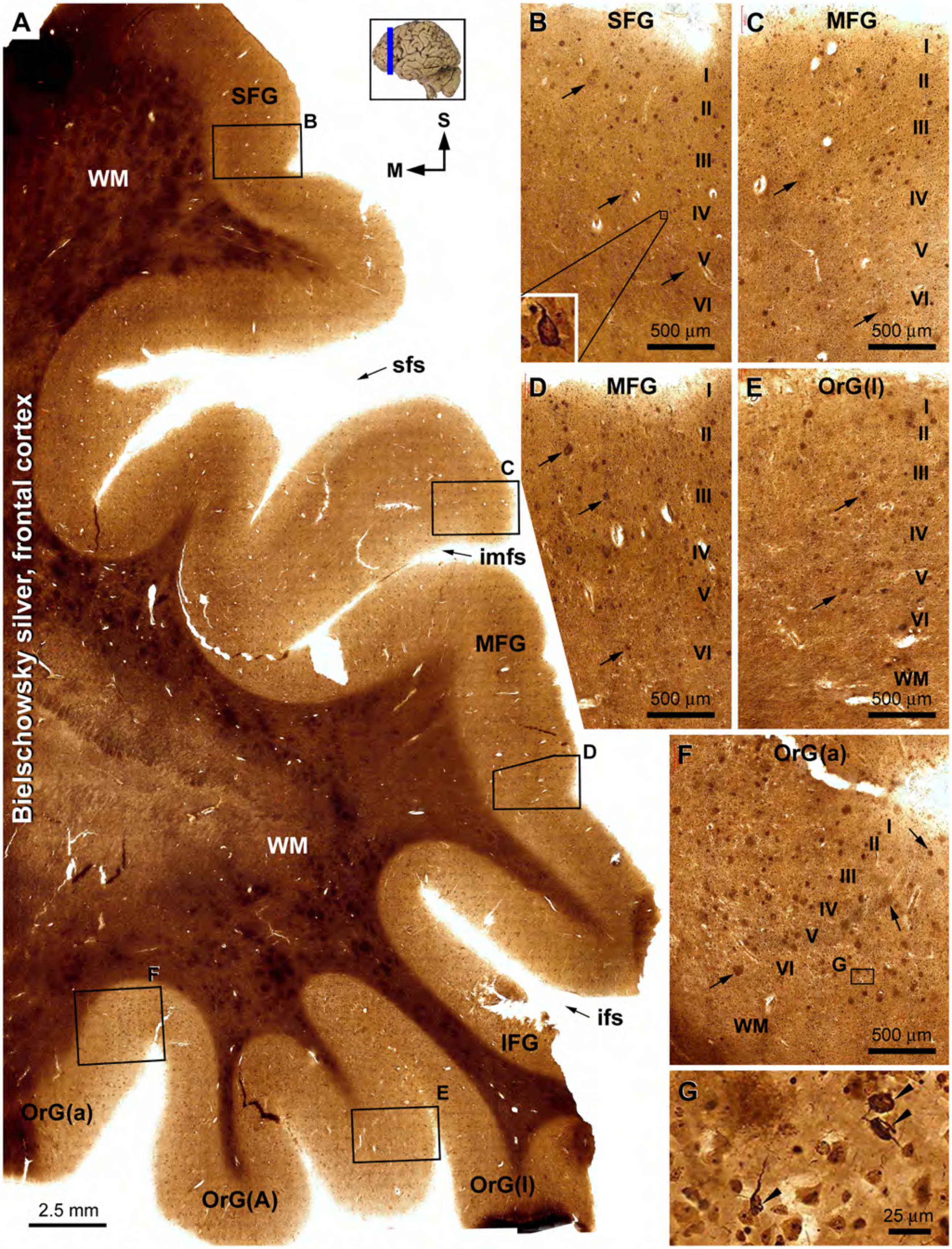

Supplemental Figure 6.5. Distribution of Bielschowsky silver stained neuritic plaques and neurons in a frontal lobe section at the level of the anterior horn from a case of Alzheimer's dementia with Thal Aβ phase 4 and Braak NFT stage IV pathologies (in support of Figure 6 in the article). Section orientation, image panel arrangement, neuroanatomical structures, cortical lamination and scale bars are as indicated (same as in supplemental Figure 6.1). The overall density of neuritic plaques (as pointed by arrows for example) appears to be comparable as moving from the SFG, MFG, IFG and orbit gyri (A-F). At higher magnification, many darkly stained neurons appear tangled (insert in B, and arrow-pointed profiles in G, shown for example). Abbreviations are as defined in Figure 6 in the article and in Supplemental Figure 6.1.

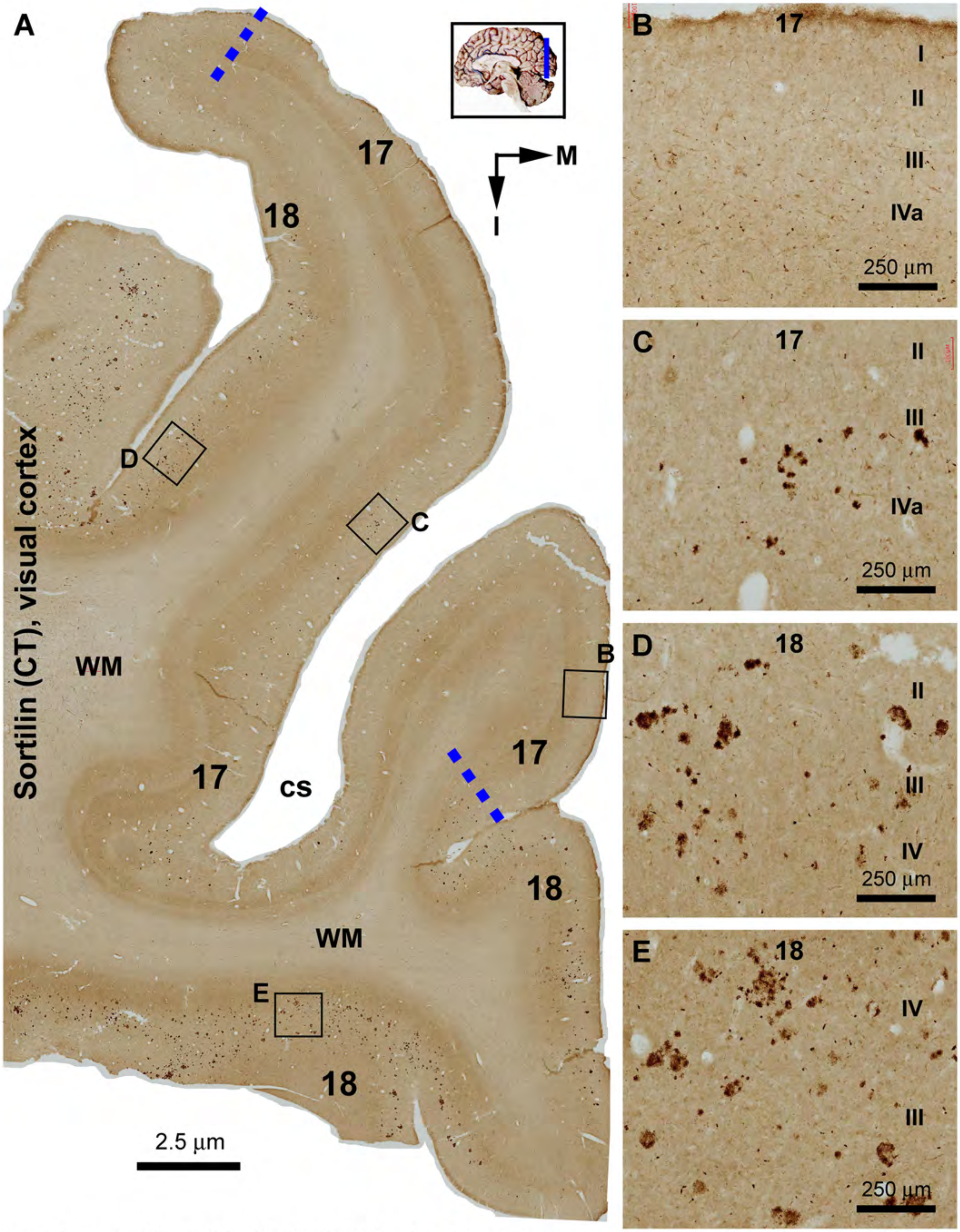

Supplemental Figure 7.1. Distribution of sorfra plaques in the visual cortex from a case with Alzheimer's dementia exhibiting Thal Aβ phase 4 and Braak NFT stage IV pathologies (referring to Figure 7 in the article). Section orientation (with M pointing to the medial and I pointing to the inferior directions in reference to anatomical position), image panel arrangement, neuroanatomical structures, cortical lamination and scale bars are as indicated. The broken blue lines mark the border between areas 17 and 18 (A). Starting from the border to area 18 and extending along the upper and low banks of calcarine sulcus (cs), sorfra plaques are infrequently seen in area 17 (A-C). In contrast, a fairly large amount of sorfra plaques occurs in the cortical gray matter in area 18 (A, D, E). Thus, there is a clear transitional change in the quantity of sorfra plaques around the border of areas 17 and 18 (A). No plaque is seen in the white matter (WM).

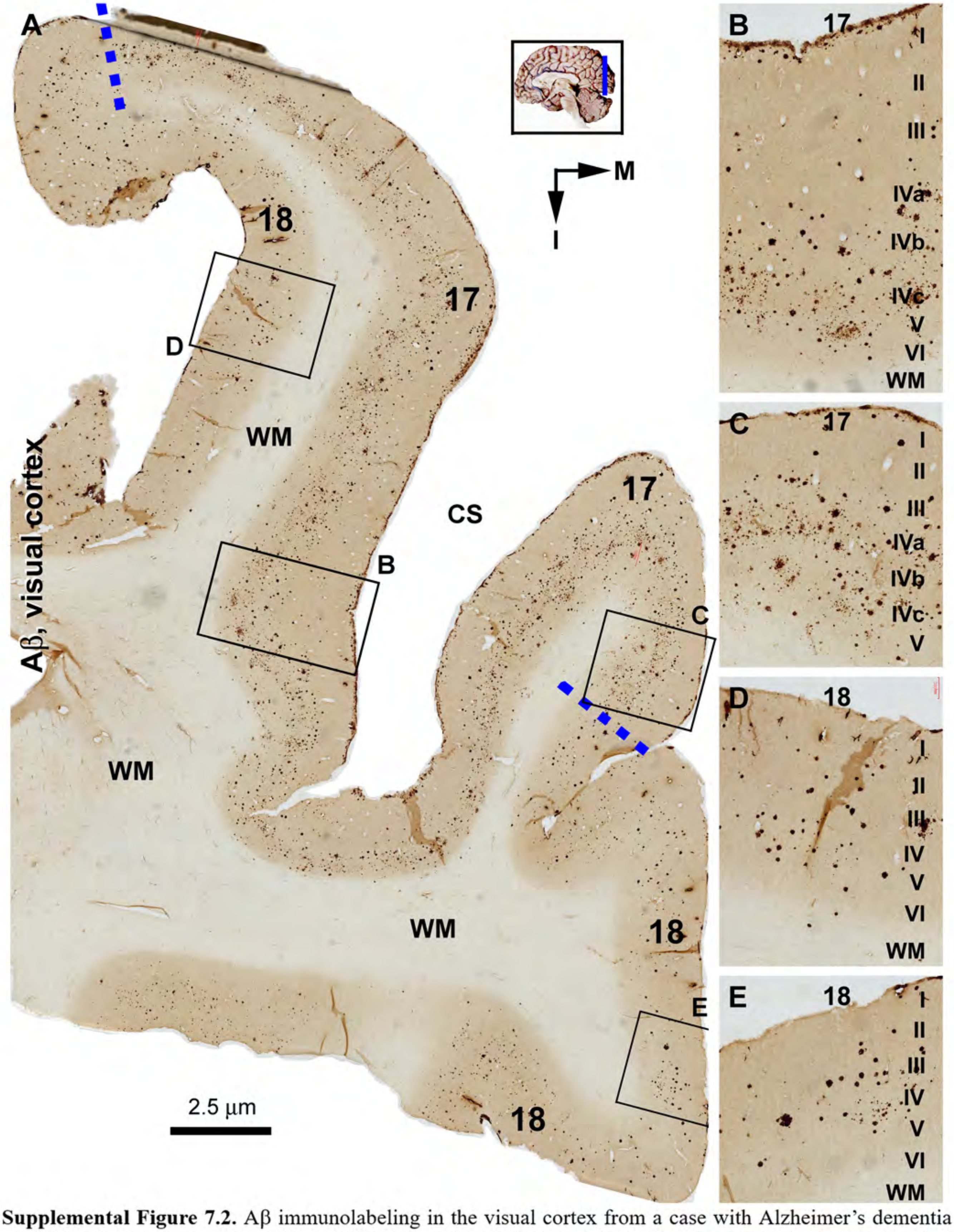

Supplemental Figure 7.2. Aβ immunolabeling in the visual cortex from a case with Alzheimer's dementia exhibiting Thal Aβ phase 4 and Braak NFT stage IV pathologies (referring to Figure 7 in the article). Section orientation, image panel arrangement (with M pointing to medial and I pointing to inferior directions in reference to anatomical position), neuroanatomical structures, cortical lamination and scale bars are as indicated. The broken blue lines mark the border between areas 17 and 18 (A). The overall amount of Aβ labeling is greater in area 17 than in area 18 (A). In area 17, Aβ labeling appears as compact and diffuse plaques, and occurs mostly in layers IVa to IVc (B, C). A patch-like distribution pattern of the plaques is noticeable in layer IVc (A-C). In area 18, the labeling appears largely as compact plaques localized to layers III-V (D, E). Meningeal and vascular Aβ deposition can be found in both visual areas (A-C). Abbreviations: cs: calcarine sulcus; WM: white matter.

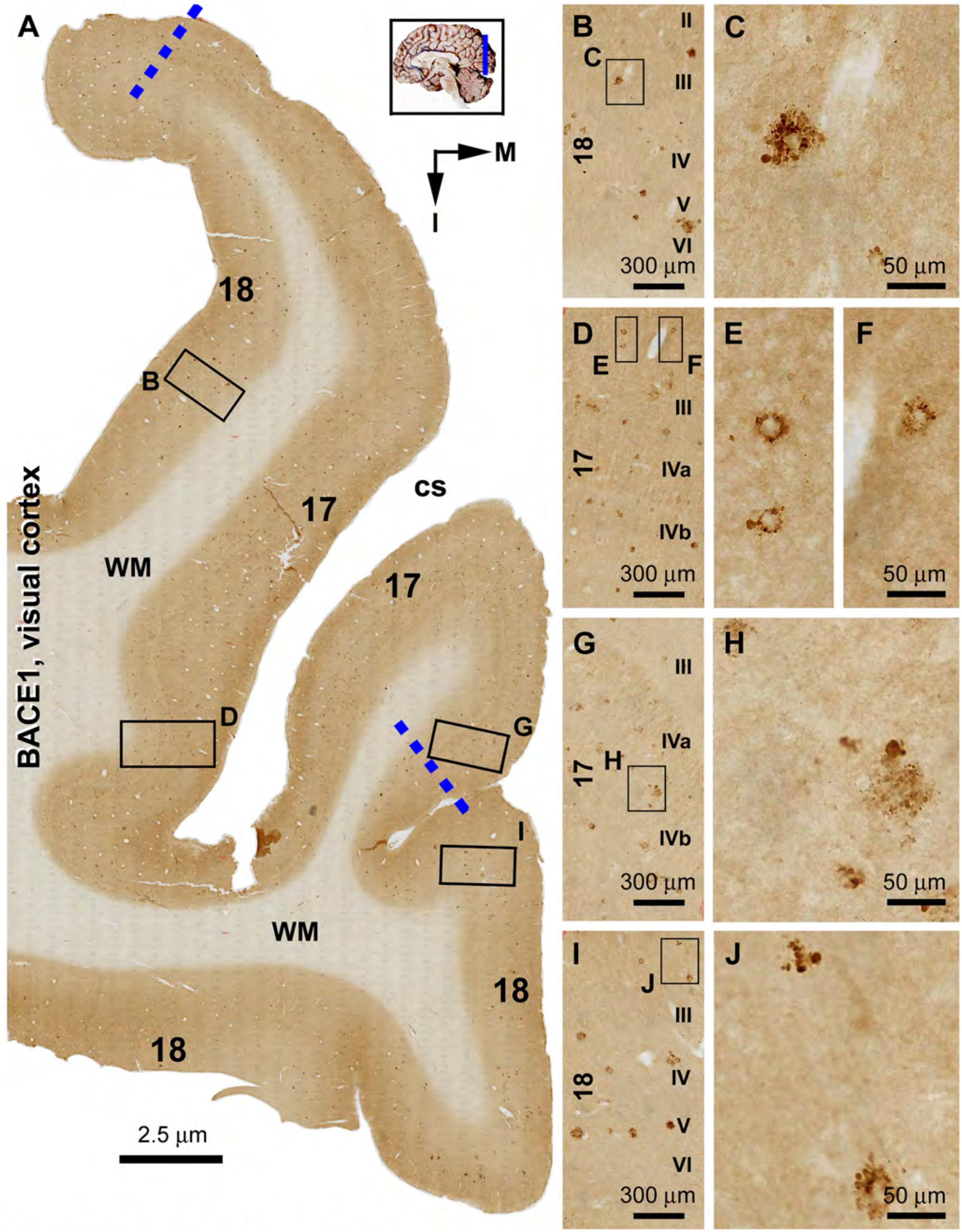

Supplemental Figure 7.3. BACE1 immunolabeling in the visual cortex of a case with Alzheimer's dementia exhibiting Thal Aβ phase 4 and Braak NFT stage IV neuropathologies (in support of Figure 7 in the article). Section orientation (same as in Supplemental Figure 7.1, image panel arrangement (with M pointing to medial and I pointing to inferior in reference to anatomical position), neuroanatomical structures, cortical lamination and scale bars are as indicated. The broken blue lines mark the border between areas 17 and 18 (A). The labeled profiles are swollen and sprouting neurites in labeling intensity, which can arrange as rosette-like clusters in varying size, with some having a pale or unstained center (C, E, F, J). The swollen neurites can also occur as isolated spherical elements (E). The overall amount of these dystrophic neurites is noticeably greater in area 17 than in area 18 while scanning across the section at higher resolution (A). Abbreviations: cs: calcarine sulcus; WM: white matter.

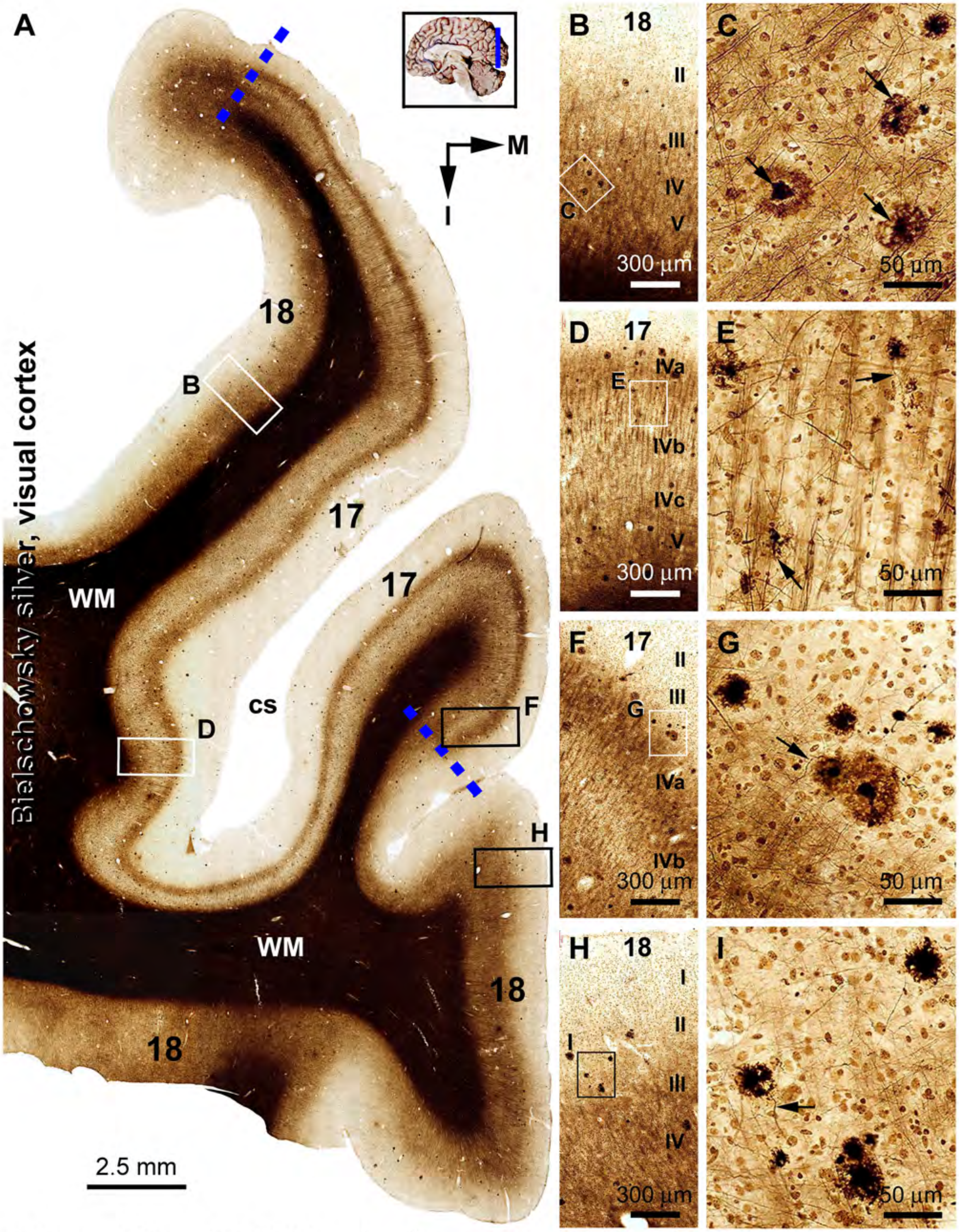

Supplemental Figure 7.4. Bielschowsky silver stain in a visual cortical section from a case with Alzheimer's dementia exhibiting Thal Aβ phase 4 and Braak NFT stage IV pathologies (referring to Figure 7 in the article). Section orientation, image panel arrangement (with M pointing to medial and I pointing to inferior directions in reference to anatomical position), neuroanatomical structures, cortical lamination and scale bars are as indicated. The broken blue lines mark the border between areas 17 and 18, with the former containing a heavily labeled band along layer IVa (A). The overall amount of neuritic plaques is noticeably greater in area 17 than in area 18, with some plaques also occurred in the layers deeper to the IVa band in area 17 (B, D, F, H). Many of the neuritic plaques contain a heavily stained core (as pointed by arrows in C). Swollen neurites at the plaque sites are seen in connection with silver-stained processes extending away from the plaques (pointed by arrows in (E,G,I). There are no tangle-like neuronal somata found in both the primary and secondary visual cortical areas. The white matter (WM) is stained darkly.

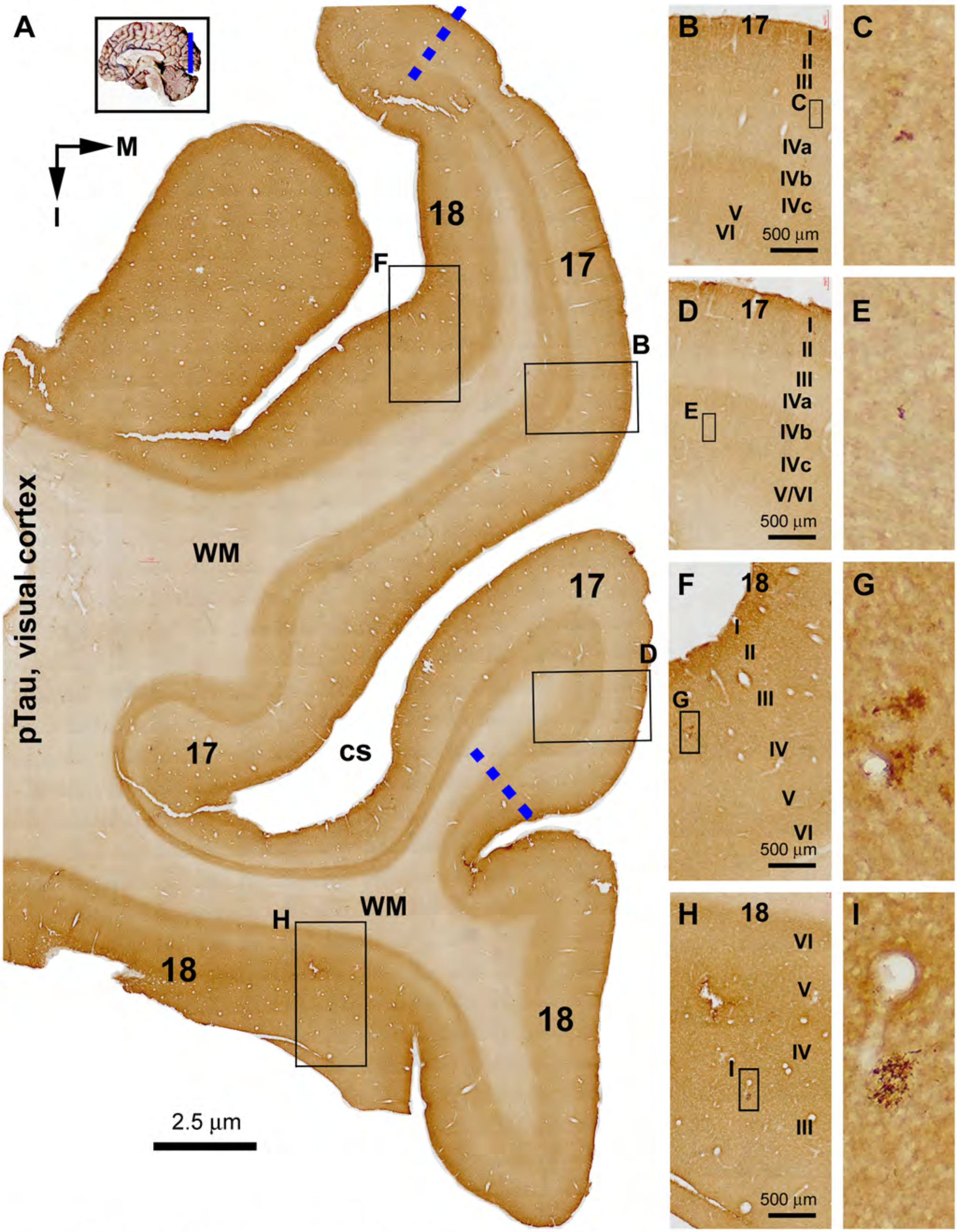

Supplemental Figure 7.5. pTau immunolabeling in the visual cortex of a case with Alzheimer's dementia exhibiting Thal Aβ phase 4 and Braak NFT stage IV pathologies (referring to Figure 7 in the article). Section orientation (same as indicated in Supplemental Figure 7.1), image panel arrangement, neuroanatomical structures, cortical lamination and scale bars are as indicated. The broken blue lines mark the border between areas 17 and 18, with the former exhibiting a stronger neuropil-like labeling in layers IVb to VI relative to the supra-granular layers (A). In both areas, pTau immunoreactive neurons and neurites can be occasionally found by searching across the entire section at high magnifications, as shown as enlarged views (C, E, G, I) from the boxed areas in (B, D, G, F). The labeled neuritic elements can also occasionally occur as small and isolated profiles (C, E), or may arrange as cluster-like structures (G, I). Abbreviations: cs: calcarine sulcus; WM: white matter.

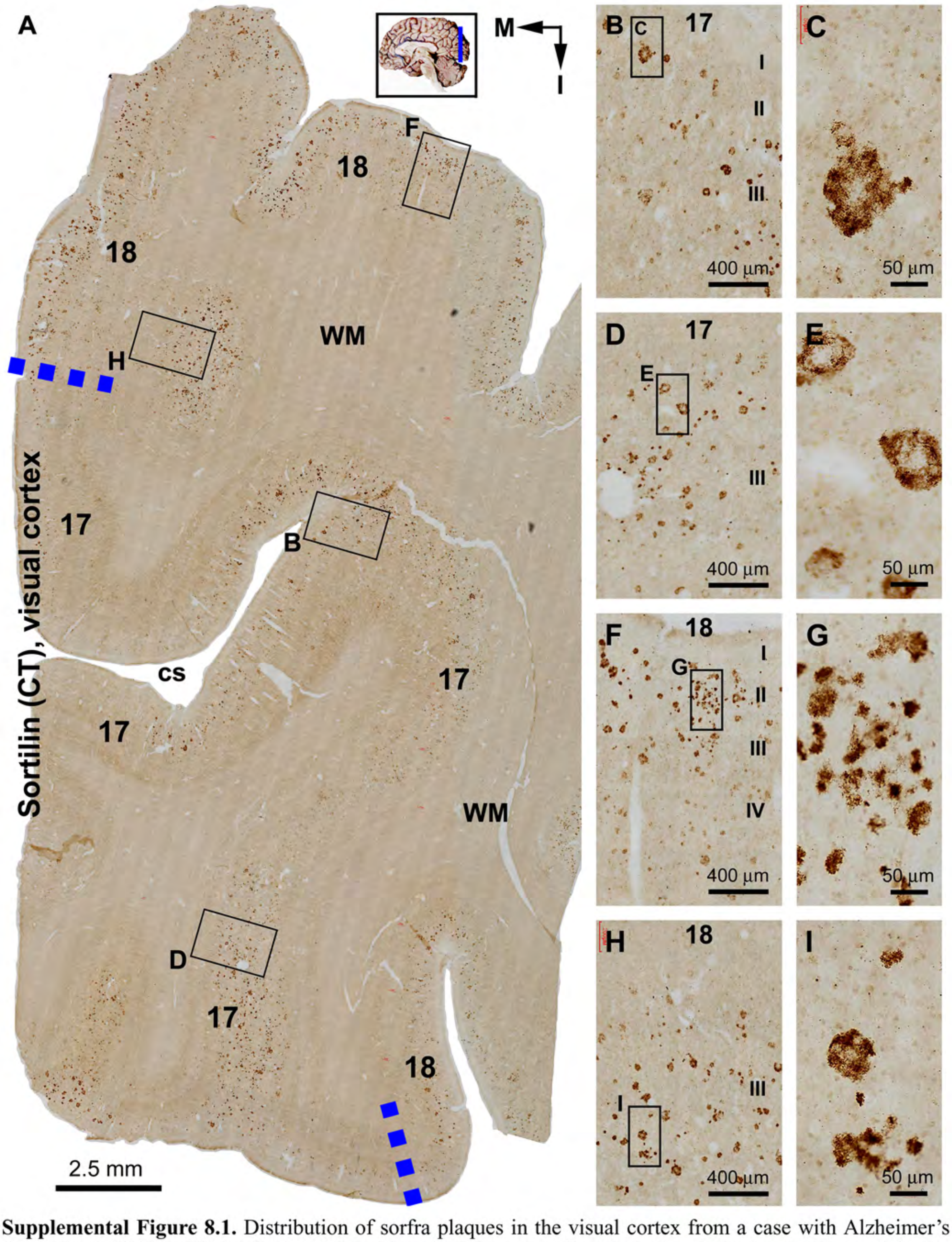

Supplemental Figure 8.1. Distribution of sorfra plaques in the visual cortex from a case with Alzheimer's dementia exhibiting Thal Aβ phase 5 and Braak NFT stage VI pathologies (referring to Figure 8 in the article). Section orientation ("M" towards medial and "I" towards inferior relative to anatomical position), image panel arrangement, neuroanatomical structures, cortical lamination and scale bars are as indicated. The broken blue lines mark the border between areas 17 and 18 (A). Sorfra plaques are present in layers II-IV, but not in the white matter (WM), in both areas 17 and 18, with the overall amount appeared greater in the secondary visual cortex (A). There exists a certain degree of variability in plaque density across area 17 along the calcarine sulcus (cs). The plaques are in varying sizes and shapes, with the large ones appeared circular in shape. Unlike Aβ plaques, sorfra plaques never contain a heavily stained core.

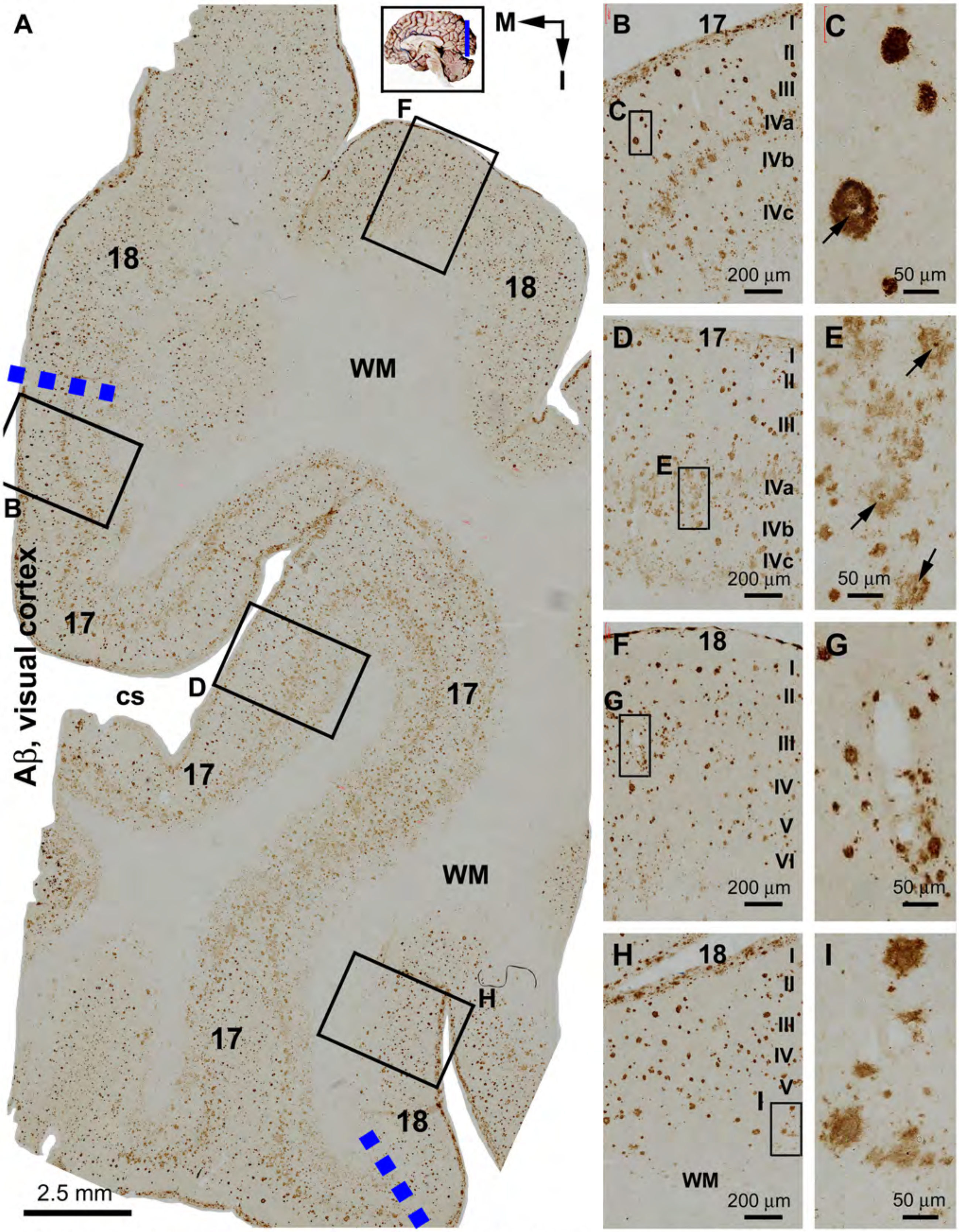

Supplemental Figure 8.2. Aβ immunolabeling in the visual cortex from a case with Alzheimer's dementia exhibiting Thal Aβ phase 5 and Braak NFT stage VI pathologies (referring to Figure 8 in the article). Section orientation (with M pointing to medial and I pointing to inferior in reference to anatomical position), image panel arrangement, neuroanatomical structures, cortical lamination and scale bars are as indicated. The broken blue lines mark the border between areas 17 and 18 (A). A great amount of Aβ labeling is present in both areas 17 and 18, more abundant in the former (A). In area 17, plaques in layers II/III appear as compact like (B-D), while those in layer IVa-c appear diffuse-like, with some also had an intensely labeled core (pointed by arrows in E). In area 18, the plaques appear largely in compact-type (F-I). Meningeal and vascular Aβ deposition are also seen in both areas (B, D, F, G, H).

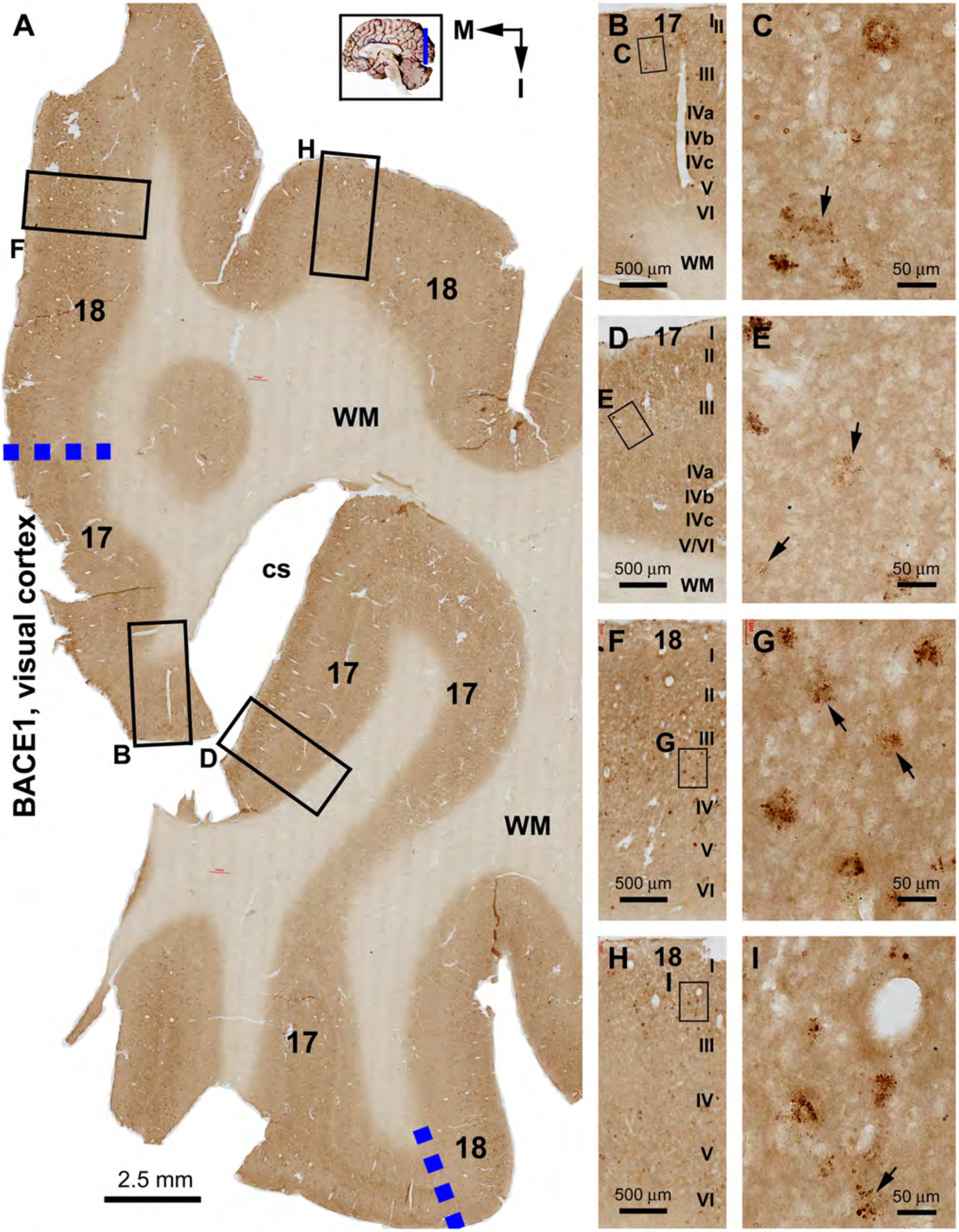

Supplemental Figure 8.3. Distribution of BACE1 immunoreactive dystrophic neurites in the visual cortex of a case with Alzheimer's dementia exhibiting Thal Aβ phase 5 and Braak NFT stage VI pathologies (referring to Figure 8 in the article). Section orientation (M: medial, I: inferior, in reference to anatomical position), image panel arrangement, neuroanatomical structures, cortical lamination and scale bars are as indicated. The broken blue lines indicate the border between areas 17 and 18, which is identifiable based on a change in the laminar pattern of labeling (A). Clusters of BACE1 labeled dystrophic neurites are seen in areas 17 and 18 over cortical layers I to VI, but not in the white matter (WM) (B-I). The overall amount neuritic clusters do not show remarkable difference between areas 17 and 18, as examined by enlarging the image to higher magnifications (A).

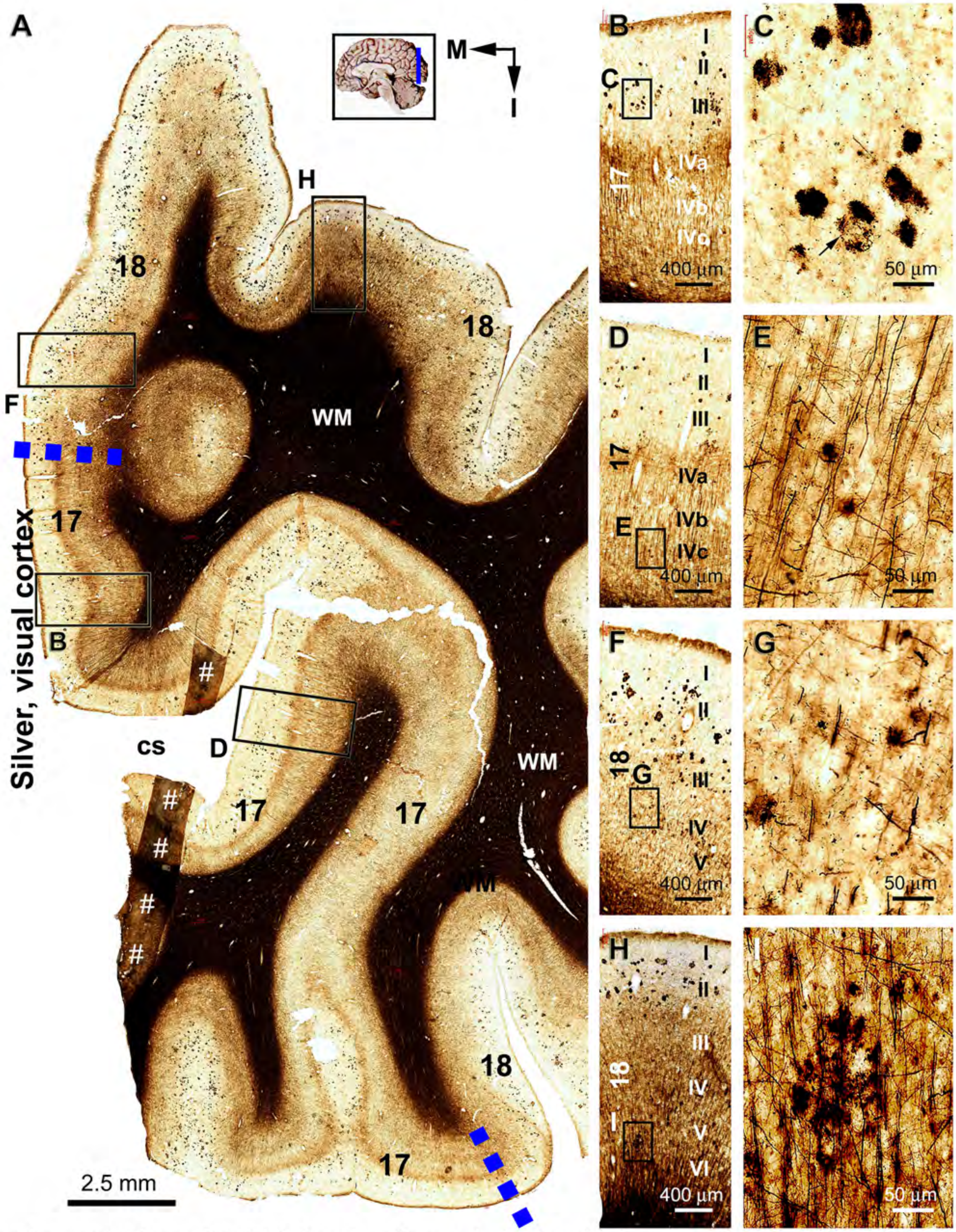

Supplemental Figure 8.4. Bielschowsky silver stain in the visual cortex of a case with Alzheimer's dementia exhibiting Thal Aβ phase 5 and Braak NFT stage VI pathologies (referring to Figure 8 in the article). Section orientation, image panel arrangement, neuroanatomical structures, cortical lamination and scale bars are as indicated. The broken blue lines indicate the border between areas 17 and 18, which is noticeable based on the presence of a silver stained band at layer IVa in area 17. It should be noted that cortex of area 17 located in the middle region of the low half in (A) is sectioned tangentially at a level deeper to the IVa band, which contains few plaques. In comparison, the cortex surrounding the calcarine sulcus (cs) is cut perpendicular to the pial surface and contains a large amount of plaques over layers II-III (A). This difference in cutting plane also exists in other figures (Supplemental Figures 8.1-8.3, 8.5) illustrating Aβ, p-Tau, sortilin and BACE1 labelings. Silver stained neuritic plaques are present in both visual cortical centers (A, B, D, F, H). At high magnification, silver-stained axon-like processes are seen entering the plaques, some of which extend vertically in the cortex, therefore likely being derived from the white matter (WM) (E, G, I). Area labeled with ### involve an artifact because of tissue folding.

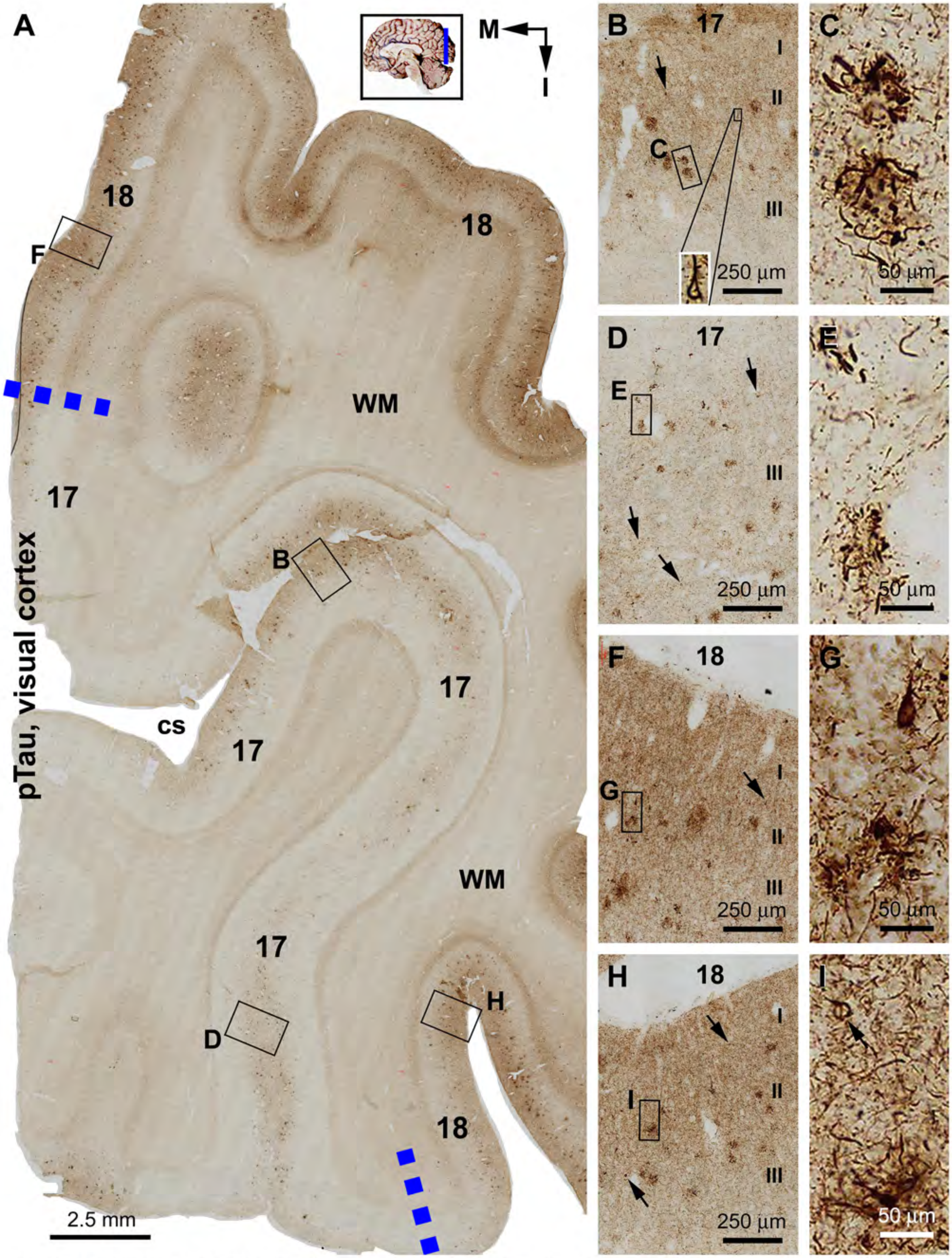

Supplemental Figure 8.5. Distribution of tauopathy in the visual cortex from a cases with AD exhibiting Thal Aβ phase 5 and Braak NFT stage VI pathologies in the brain (referring to Figure 8 in the article). Section orientation (M: medial; I: inferior, relative to anatomical position), image panel arrangement, neuroanatomical structures, cortical lamination and scale bars are as indicated. The broken blue lines mark the border of areas 17 and 18, which is identifiable based on a laminar pattern change of the immunolabeling (A). pTau labeling occurs more intense in layers I-III than in the deeper layers in both areas 17 and 18. The overall amount of labeling appears greater in area 18 than in area 17, while the amount of labeling is also variable across area 17 due to a difference in sectioning plane as noted in Supplemental Figure 8.4. At high magnifications, pTau positive processes are densely packed in the same configuration as seen for dystrophic neurites of neuritic plaques (B-I). Tangled neuronal somata are observed in both areas (as seen in G for example). Abbreviations: cs: calcarine sulcus; WM: white matter.

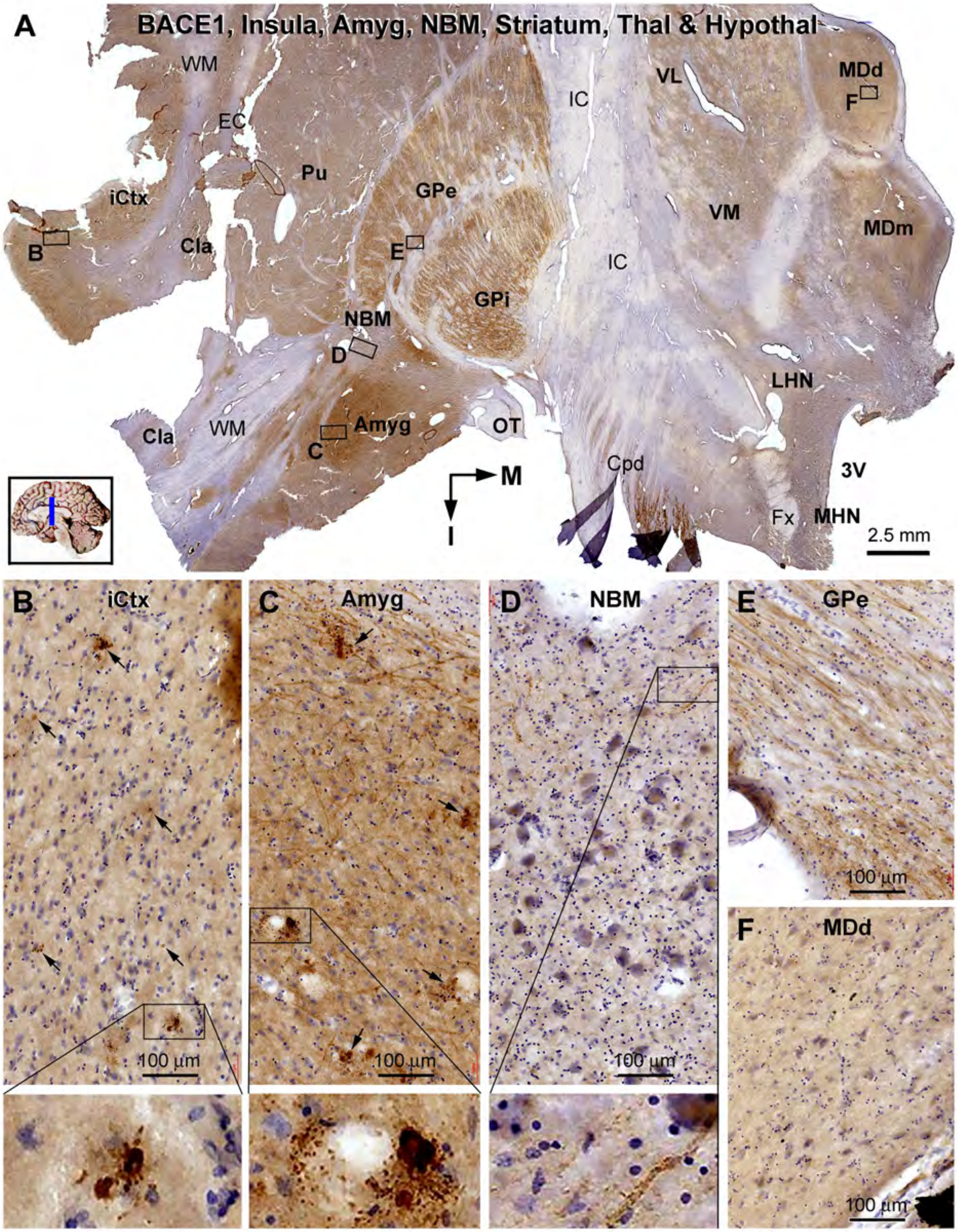

Supplemental Figure 9.1. BACE1 immunolabeling (counterstained with toluidine blue) in the insular cortex, amygdala, basal forebrain nucleus, striatum and diencephalon in an AD brain exhibited Thal phase 5 and Braak V stage neuropathologies. Section orientation, anatomical regions, figure panel arrangement and scale bars are as indicated in individual panels. Panel (A) is a low magnification image of the immunolabeling over the entire section covering multiple telecephalic and diencephalic structures. Framed areas in (A) are enlarged and shown as panels (B-F). BACE1 labeled dystrophic neurites occurring clusters or isolated spherical profiles are only found in the insular cortex (iCtx) (B) and the amygdaloid complex (Amyg) (C). Normal looking neuronal processes are found in the Amyg (C) and the nucleus basalis of Meynert (NBM) (D), and especially prominent in the external (GPe) and internal (GPi) divisions of the globus pallidus (GP) (A, E). No somatic labeling is seen in any of the above telecephalic and diencephalic structures. Light, background-like, neuropil immunoreactivity is seen in the in the claustrum (Cla), putamen (Pu), and thalamic and hypothalamic subdivisions (A, F). Additional abbreviations: the dorsal (MDd) and medial (MDm) divisions of the mediodorsal nucleus (MD) of thalamus (A, H) and the lateral (LHN) and medal (MHN) hypothalamic nucleus (HN) (A, I). Additional abbreviations: EC: external capsule; Fx: fornix; IC: internal capsule; VL: ventrolateral thalamic nucleus; VM: ventromedial thalamic nucleus; Cpd: cerebral peduncle; OT: optic tract; 3V: third ventricle; WM: white matter.

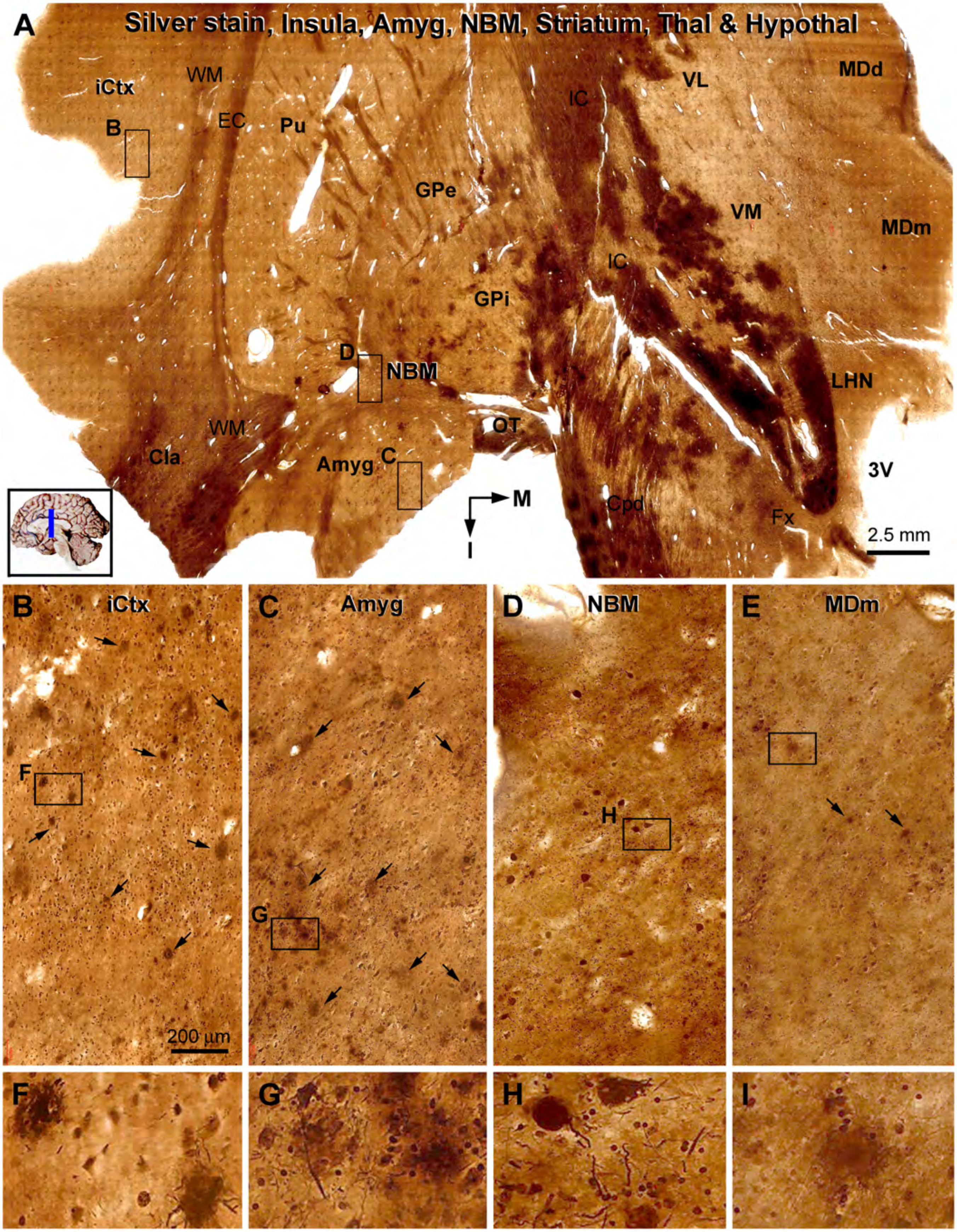

Supplemental Figure 9.2. Bielschowsky silver stain in the insular cortex, amygdala, basal forebrain nucleus, striatum and diencephalon in an AD brain exhibited Thal phase 5 and Braak V stage neuropathologies. Section orientation, anatomical regions, figure panel arrangement and scale bars are as indicated in individual panels. Panel (A) is a low magnification image of the silver stain across the telecephalic and diencephalic structures, with the framed areas enlarged as panels (B-I). Neuritic plaques are evident in the insular cortex (iCtx) (B, F) and the amygdaloid complex (Amyg) (C, G). In the nucleus basalis of Meynert (NBM), silver stained neuronal somata are present, whereas no neuritic plaques are found (D, H). Some small plaques are seen in the thalamic nuclei, which are not apparently associated with dystrophic neurites (E, I). Additional abbreviations are as defined in Supplemental Figure 9.1.

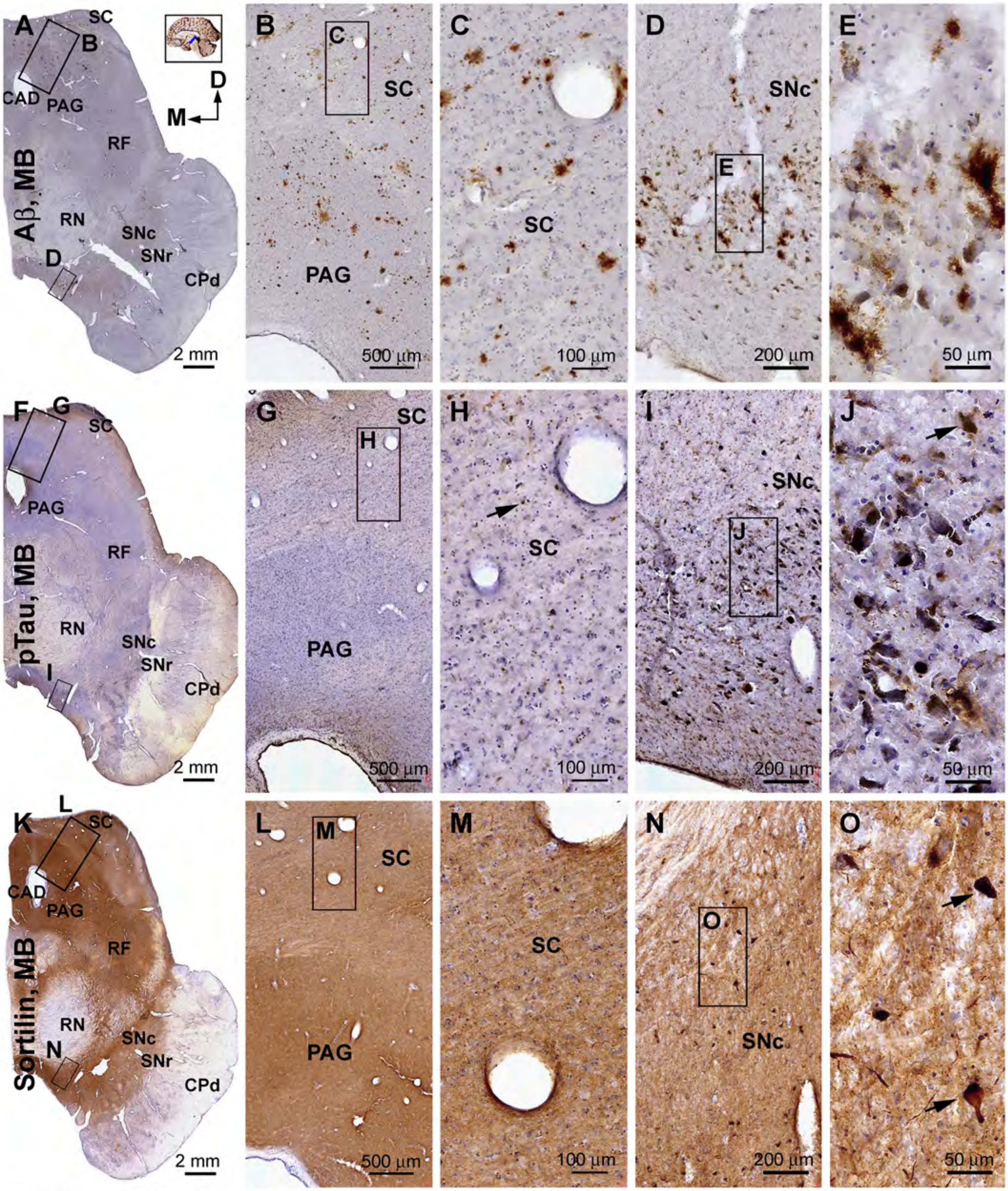

Supplemental Figure 10.1. Comparative assessment of Aβ, pTau and sortilin C-terminal antibody immunolabeling (counterstained with toluidine blue) in adjacent sections of the midbrain (MB) from an AD brain with Thal Aβ phase 5 and Braak NTF stage VI neuropathologies. Section orientation, immunolabeling markers, image panel arrangement, neuroanatomical designation and scale bars are as indicated. Panel (A) shows Aβ labeling that is present widely across the section except for the white matter regions. At high magnification, a large number of small-sized diffuse-like plaques are present in the periaqueductal gray (PAG) and superior colliculi (SC) (B, C). These plaques spread ventrally over the reticular formation (RF) (A) into the substantia nigra pars compact (SNc) (A, D, E). No sorfra plaques occur in the above midbrain areas, while the nigral dopaminergic neurons exhibited sortilin immunoreactivity (F-J). pTau labeled neurons are only present in the SNc (K-O). Additional abbreviations: CAD: cerebral aqueduct; RN: red nucleus; SNr: substantia nigra pars reticulate; Cpd: cerebral peduncle.

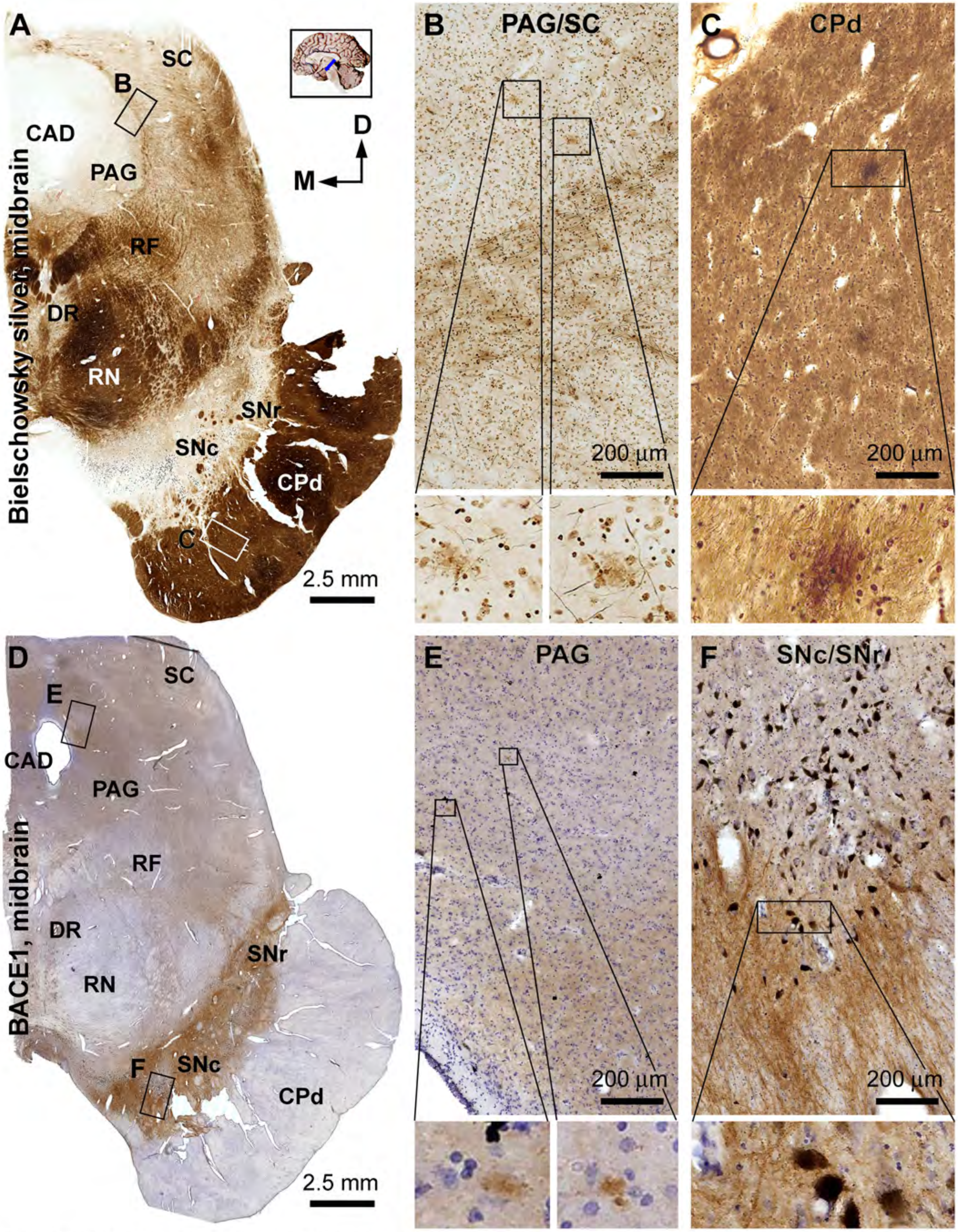

Supplemental Figure 10.2. Bielschowsky silver stain and BACE1 immunolabeling (with toluidine blue counterstain) in adjacent sections of the midbrain (MB) from an AD brain with Thal Aβ phase 5 and Braak NTF stage VI neuropathologies. Section orientation, image panel arrangement, neuroanatomical designation and scale bars are as indicated. In silver stain, faintly stained small plaques could be recognized at high magnifications in the gray and white matter areas, which occur as amorphous silver staining not associated with dystrophic neurites (A-C). BACE1 immunolabeling appears as neuropil-like labeling over the areas of gray matter regions and RF. Clearly labeled fibrous profiles are present SNr and SNc (D-F). Abbreviations are as defined in Supplemental Figure 10.1.

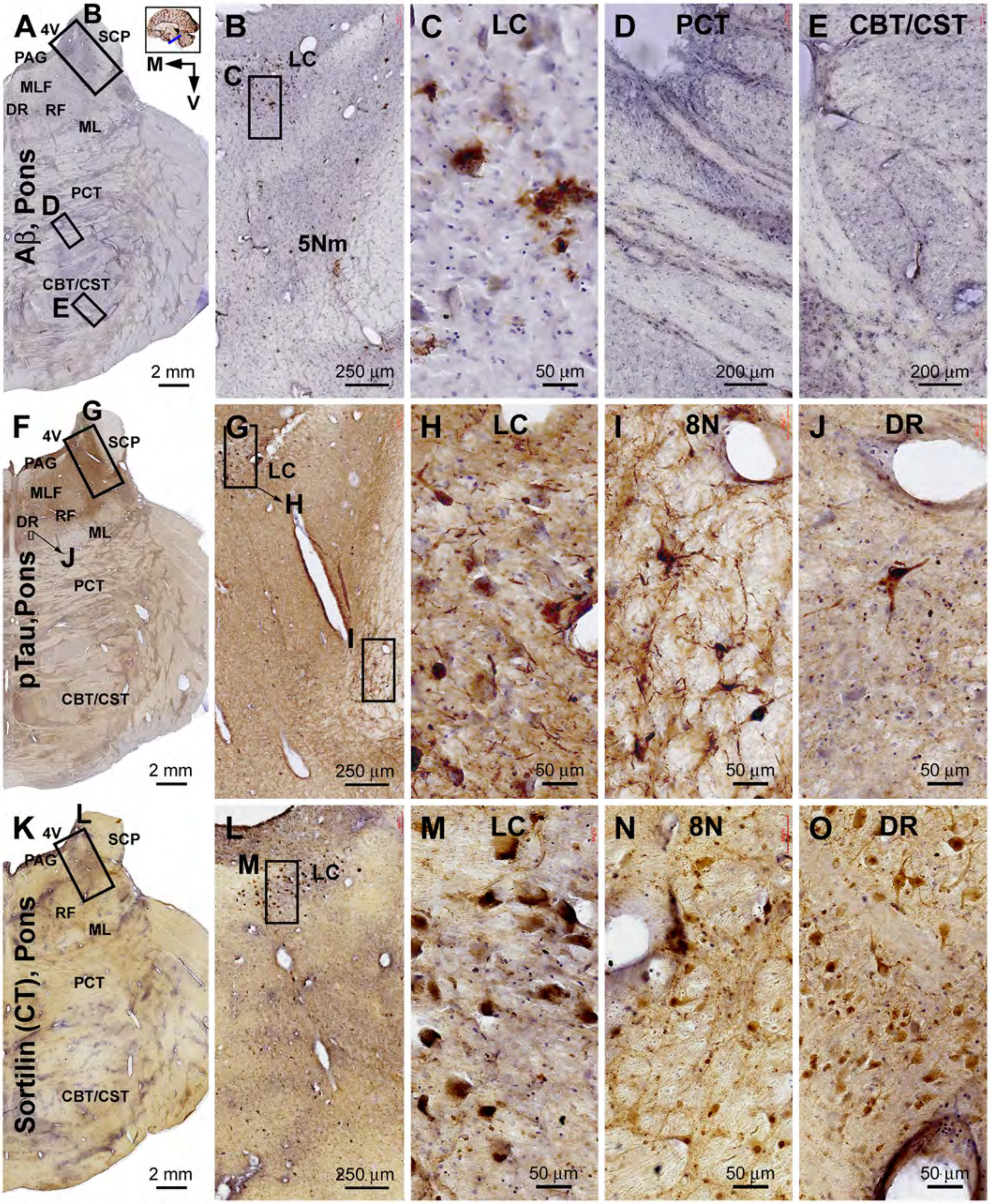

Supplemental Figure 11.1. Comparative assessment of Aβ, pTau and sortilin C-terminal antibody immunolabeling (with toluidine blue counterstain) in adjacent sections of the pons from an AD brain with Thal Aβ phase 5 and Braak NTF stage VI neuropathologies. Section orientation, immunolabeling markers, image panel arrangement, neuroanatomical designation and scale bars are as indicated. Aβ labeling appearing as diffuse plaques occurs largely in the locus coeruleus (LC), reticular formation (RF) and dorsal raphe (DR) (A-C), but not in the white matter regions (A, D, E). The sortilin antibody labels neuronal somata in the LC, DR, RF and the cranial nerve nuclei, whereas no extracellular plaque profiles are found (F-J). pTau immunoreactive neuronal profiles also occur in these same locations (K-O). Additional abbreviations: 5Nm: motor nucleus of the trigeminal nerve; 8N: vestibular nerve nuclei; 4V: fourth ventricle; PAG: periaqueductal gray substance; SCP: superior cerebellar peduncle; CBT and CST: corticobulbar and corticospinal tracts; ML: medial lemniscus (ML); MLF: median longitudinal fasciculus (MLF).

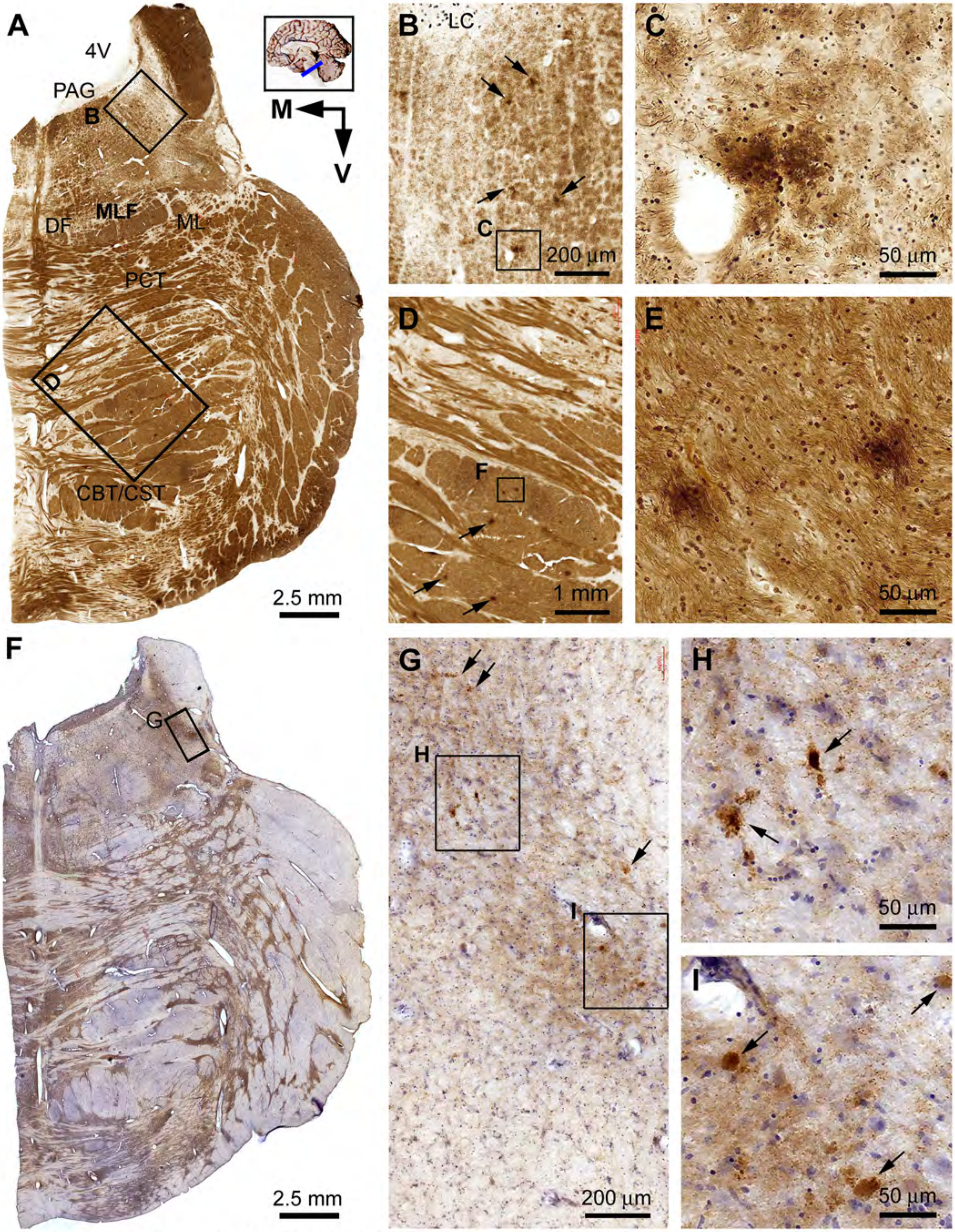

Supplemental Figure 11.2. Bielschowsky silver stain and BACE1 immunolabeling (toluidine blue counterstain) in adjacent sections of the pons from an AD brain with Thal Aβ phase 5 and Braak NTF stage VI neuropathologies. Section orientation, image panel arrangement, neuroanatomical designation and scale bars are as indicated. Small areas (pointed by arrows) with enhanced amorphous silver staining are identifiable in both the gray and white matter parts. However, no typical swollen dystrophic neurites are present in the vicinity of these plaque-like structures (A-E). In BACE1 immunolabeling, small-sized neuritic clusters or isolated swollen neurites (pointed by arrows) are occasionally identifiable by close examination (F-I). Abbreviations are as defined in Supplemental Figure 11.1.

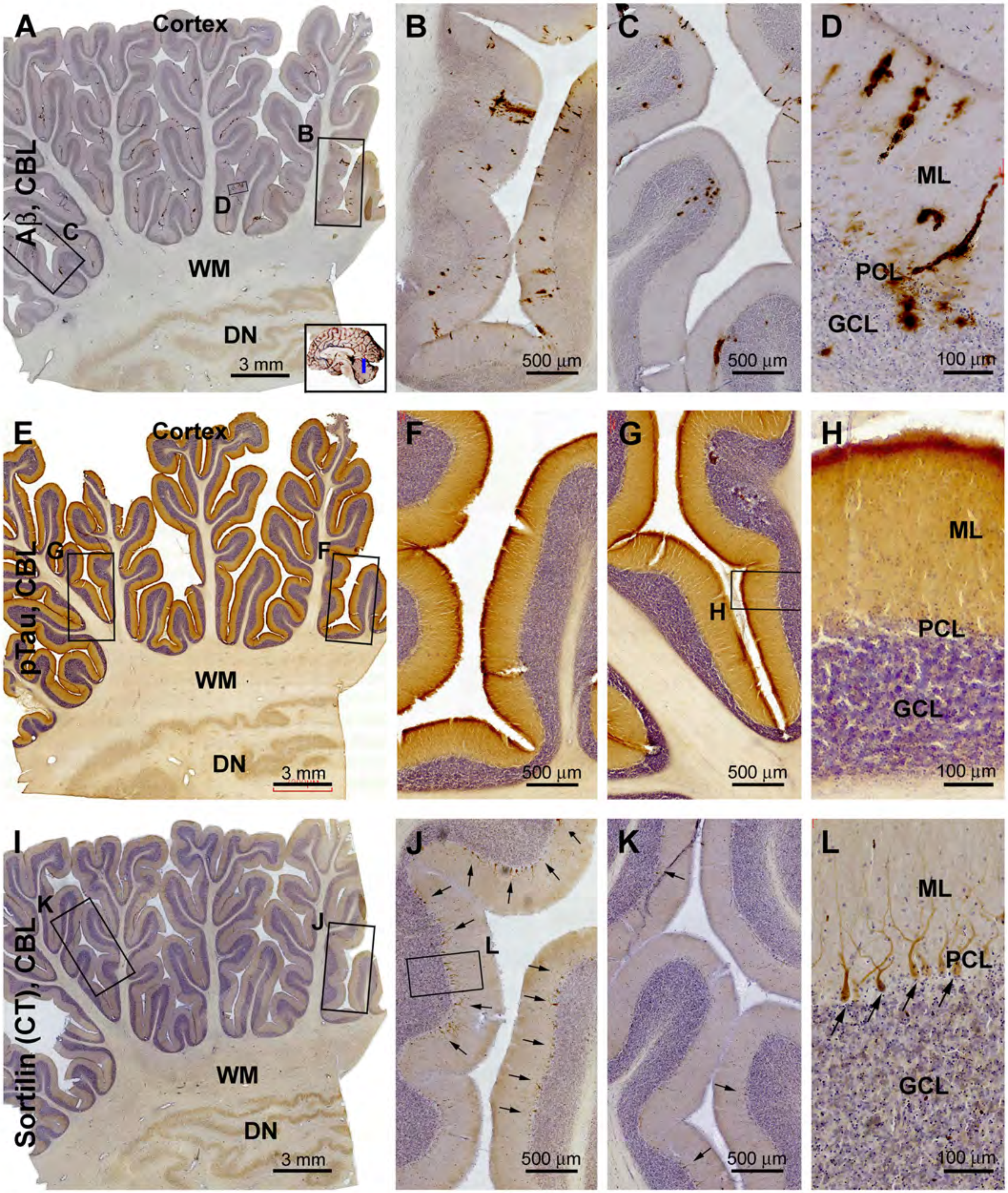

Supplemental Figure 12.1. Aβ, pTau and sortilin C-terminal (CT) antibody immunolabeling (with toluidine blue counterstain) in adjacent sections of the cerebellum (CLB) from an AD brain with Thal Aβ phase 5 and Braak NTF stage VI neuropathologies. Panel arrangement, lamination and scale bars are as indicated. Diffuse Aβ deposition is present in the molecular layer (ML) and granule cell layer (GCL) in some folia, vascular and meningeal deposition also seen in some regions (A-D). pTau immunolabeling appears as even neuropil reactivity over the ML, with no labeled neuronal somata or neuritic profiles detectable (E-H). The sortilin antibody visualizes light immunolabeling in the ML and dentate nucleus (DN), but no extracellular lesions could be identified (I-K). Notably, a subpopulation of Purkinje cells (pointed by arrows) in the folia with Aβ deposition show enhanced sortilin immunoreactivity in the somata and dendritic processes (J-L). Additional abbreviations: WM: white matter; PCL: Purkinje cell layer.

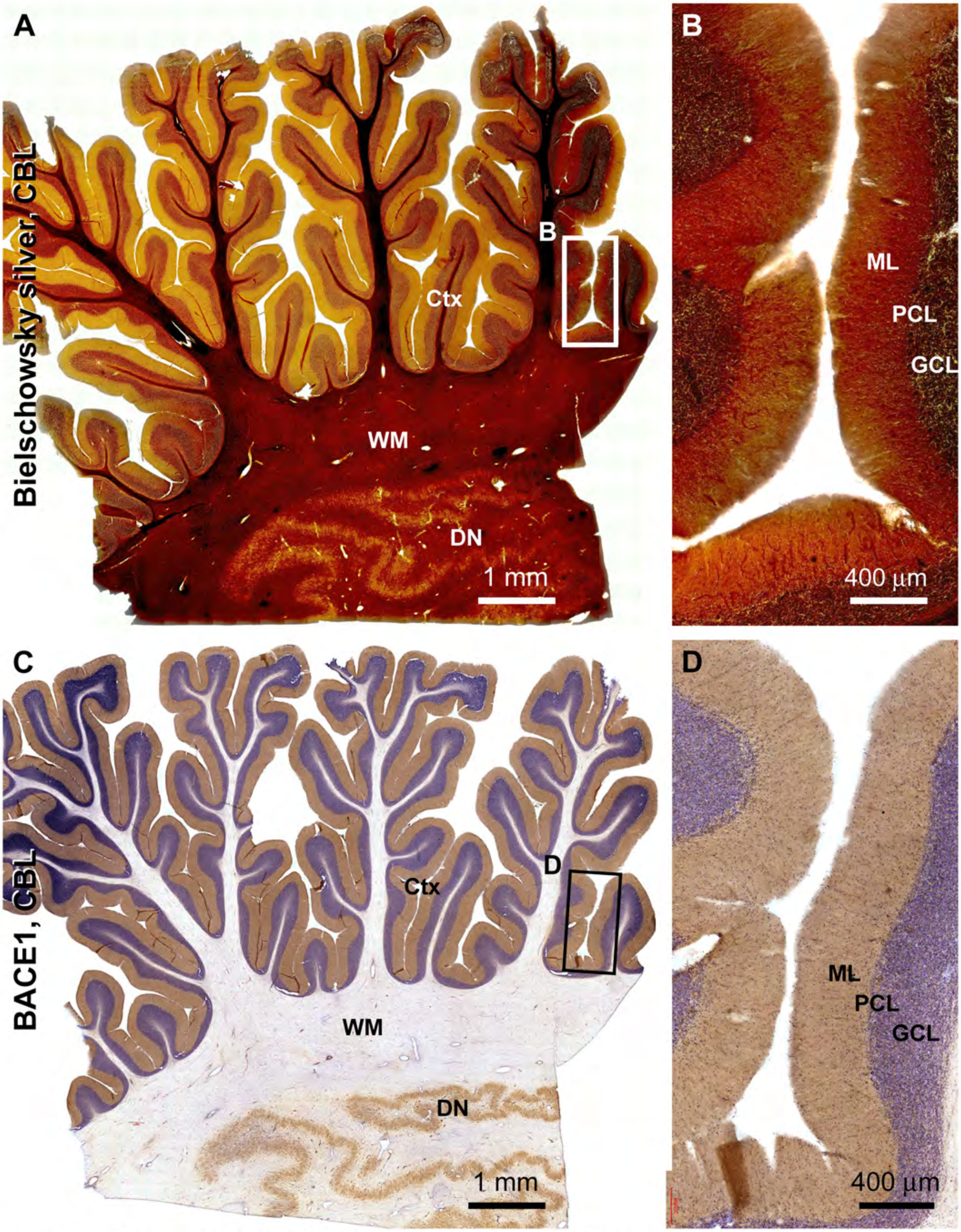

Supplemental Figure 12.2. Bielschowsky silver stain (A, B) and BACE1 immunolabeling (C, D) in adjacent sections of the cerebellum（CBL）from an AD brain with Thal Aβ phase 5 and Braak NTF stage VI neuropathologies. Section orientation, image panel arrangement, neuroanatomical designation and scale bars are as indicated. In silver stain, no neuronal somata or neuritic plaques could be identified, including in the folia with Aβ deposition (referring to Supplemental Figure 12.1). BACE1 immunolabeling (counterstained with toluidine blue) occurs as neuropil-like reactivity present evenly over the ML, with little reactivity in the Purkinje cell and granule cell layers (PCL, GCL). No dystrophic neuritic profiles are identifiable in the cortex and white matter (WM) and the dentate nucleus (DN).
